# A Case of Painful Visual Loss - Managing Orbital Compartment Syndrome in the Emergency Department

**DOI:** 10.21980/J8N35D

**Published:** 2024-10-31

**Authors:** Jessica Pelletier, Alexander Croft, Michael Pajor, Matthew Santos, Douglas Char, Marc Mendelsohn, Ernesto Romo

**Affiliations:** * Department of Emergency Medicine, Washington University School of Medicine, St. Louis, MO; ^ Wills Eye Hospital, Philadelphia, PA

## Abstract

**Audience:**

Emergency medicine (EM) residents. This simulation curriculum may also be utilized for senior medical students conducting EM rotations.

**Background:**

Ophthalmologic education represents only a small portion of medical school curriculums and continues to decrease over time, leaving physicians poorly equipped to diagnose and manage eye complaints.^1^ Of emergency physicians (EPs) surveyed, 72.5% felt that they could diagnose orbital compartment syndrome (OCS), yet only 40.3% felt comfortable performing a necessary lateral canthotomy and cantholysis (LCC).^2^ These survey results demonstrate the urgent need for improved ophthalmology education in EM residency to help us diagnose and manage potentially vision-threatening pathology.

**Educational Objectives:**

By the end of this simulation, learners will be able to: 1) demonstrate the major components and a systematic approach to the emergency ophthalmologic examination, 2) develop a differential diagnosis of sight-threatening etiologies that could cause eye pain or vision loss, 3) demonstrate proficiency in performing potentially vision-saving procedures within the scope of EM practice.

**Educational Methods:**

Low-fidelity simulation was conducted using a novel model adapted from that used by Phillips et al. during their ophthalmology day in the Department of Emergency Medicine at Vanderbilt University.^3^ The simulation case was developed by an interdepartmental team of ophthalmologists and EPs at our institution.

**Research Objectives:**

To evaluate for statistically significant changes in self-efficacy, knowledge, and performance after an educational intervention. Our primary outcome was defined as a checklist-based performance on a simulated case of orbital compartment syndrome necessitating LCC.

**Research Methods:**

We conducted a single-center prospective pre- and post-interventional study evaluating the impact of an educational intervention on EM resident management of a simulated case of OCS. Our two-part study intervention consisted of a lecture on OCS followed by a four and a half hour ophthalmology education day (OED). Residents were evaluated using self-efficacy scales (SES), multiple-choice questions (MCQ), and a performance checklist (developed via a modified Delphi process) at three timepoints: Pre-intervention, immediate post-intervention, and three months post-intervention. Post-graduate year (PGY)-1 through PGY-4 EM residents at an Urban Level 1 Trauma Center participated.

**Results:**

Initial recruitment consisted of 18 residents (PGY-1 through PGY-4), and 16 residents (PGY-1 through PGY-3) completed the study. Nine residents participated in the OED and seven residents did not. There were no pre-existing differences in median checklist-based performance, MCQ, or SES scores prior to the intervention. At three months post-OED, the OED attendees scored statistically significantly higher on checklist-based performance than non-attendees (lecture only).

**Discussion:**

Ophthalmology education in physician training is limited, and EP comfort with performing vision-saving procedures is poor. We developed a simulation case involving such a vision-saving procedure as well as an ophthalmology curriculum that increased skill retention surrounding management of ophthalmologic emergencies.

**Topics:**

Emergency medicine (EM), ophthalmology, orbital compartment syndrome (OCS), retrobulbar hematoma, vision loss, eye pain.

## USER GUIDE

[Table t2-jetem-9-4-s1]

**Table t2-jetem-9-4-s1:** 

List of Resources:	
Abstract	1
User Guide	3
Instructor Materials	11
Operator Materials	24
Debriefing and Evaluation Pearls	26
Simulation Assessment	29
Appendix A: Lateral Canthotomy/Cantholysis Model: How to Guide	34
Appendix B: Retrobulbar Hemorrhage, Canthotomy/Cantholysis PowerPoint	38
Appendix C: Lateral Canthotomy/Cantholysis Checklist	39
Appendix D: MCQ Test and Answers for Assessment Before and/or After Participation in the OCS Simulation Case	41
Appendix E: Self-Efficacy Scale (SES)	48
Appendix F: Stimuli for Simulation Case	49

**Learner Audience:**

Medical Students, Interns, Junior Residents, Senior Residents

**Time Required for Implementation:**

**Instructor Preparation:** Instructors should set aside 60 minutes to review and prepare for running the case itself. Thirty minutes is needed to initially set up the orbital compartment syndrome (OCS) model. Resetting the model between repetitions of the same case requires 5 minutes.

**Time for case:** 15–20 minutes is required for completion of the case.

**Time for debriefing:** 20 minutes is required for debriefing the case.

**Recommended Number of Learners per Instructor:**

No more than four learners to one instructor. We conducted these simulations in a single learner: single instructor fashion; however, this is time-intensive for instructors and may also induce more anxiety among learners (as simulation in groups has been shown to reduce cognitive load).^4^

**Topics:**

Emergency medicine (EM), ophthalmology, orbital compartment syndrome (OCS), retrobulbar hematoma, vision loss, eye pain.

**Objectives:**

By the end of this simulation, learners will be able to:

1. Demonstrate the major components of and a systematic approach to the emergency ophthalmologic examination
2. Develop a differential diagnosis of sight-threatening etiologies that could cause eye pain or vision loss
3. Demonstrate proficiency in performing potentially vision-saving procedures within the scope of EM practice

### Linked objectives and methods

**Objective 1** - *Demonstrate the major components of and a systematic approach to the emergency ophthalmologic examination* - Learners are specifically observed approaching a patient with painful visual loss during this simulated case. The instructor notes the order of steps in which the learner performs the ophthalmologic examination and discusses ways that they can improve their systematic approach during the debriefing; specifically, what portions of the ophthalmologic examination may have been missing. A key point for learners to take away from this discussion is that intraocular pressure (IOP) should only be assessed AFTER an open globe injury (OGI) has been ruled out via a thorough gross examination, pupillary examination, and fluorescein staining, as placing undue pressure on the eye could result in extrusion of intraocular contents. Learners may be able to recite the importance of this in an oral board-style setting, but when pushed to practically apply this knowledge, many learners were observed to miss this appropriate order of steps. Repeat assessment over time demonstrated improved checklist-based performance on the simulated case of OCS, suggesting that simulation is an ideal format to help learners to retain this information.

**Objective 2** - *Develop a differential diagnosis of sight-threatening etiologies that could cause eye pain or vision loss* - During the simulated case, learners encounter a patient who has painful visual loss after significant trauma to the eye. They will need to recognize the possibility that several vision-threatening diagnoses that require immediate intervention could account for this presentation, including OCS and OGI. This differential diagnosis is discussed at length during the debriefing and helps reinforce the importance of ruling out these diagnoses as well as involving ophthalmology early in the management of patients with ophthalmologic emergencies.

**Objective 3** - *Demonstrate proficiency in performing potentially vision-saving procedures within the scope of EM practice* - In the simulated case of OCS, learners are expected to perform a lateral canthotomy and cantholysis (LCC) given the time-sensitive nature of this procedure. While practicing this procedure in isolation via a procedural training station is helpful, the pressure of treating a simulated patient with OCS adds a layer of stress and anxiety that more closely mimics the true ED environment in which this procedure would be performed. In other words, while a LCC procedural station would allow for engagement of intrinsic and germane cognitive load, a simulation setting provides the addition of extraneous cognitive load.

### Recommended pre-reading for instructor

- Dupre A, Vojta L. Red and painful eye. In Walls RM, Hockberger RS, Gausche-Hill M, et al, eds. *Rosen’s Emergency Medicine: Concepts and Clinical Practice*, Vol 10. Philadelphia, PA: Elsevier, 2023.
- Perin A, Bayram J, Uwaydat S. Acute orbital compartment syndrome (retrobulbar hemorrhage) management. In Fielding A, Davis KJ, eds. *Reichman’s Emergency Medicine Procedures*, 3rd ed. China: McGraw-Hill Education, 2019.
- Vietvuong V. Lateral Canthotomy. In Mattu A, Swadron S, eds. *CorePendium*. Burbank, CA: CorePendium, LLC, Updated October 26, 2022. Accessed February 18, 2023. At: https://www.emrap.org/corependium/chapter/recVOp8GoTweO76o2/Lateral-Canthotomy

### Learner responsible content

If the simulated OCS case will be included as part of an ophthalmology education day (OED) or “bootcamp,” we recommend that learners receive the following prior to engaging in the simulation:

1. Receiving a lecture on OCS - The following presentation was created by a member of our study team (Appendix B)
2. Practicing LCC on the same model used in the simulated OCS case in order to familiarize learners with the model

If the simulated OCS case will NOT be conducted as part of an OED or bootcamp, we recommend that learners are provided with the aforementioned slideshows to review before the simulation. They should also review the following to substitute for practicing on an OCS model:

- *Lateral Canthotomy.* Emergency Medicine Reviews and Perspectives (EM:RAP); 2016. Accessed January 6, 2024. At: https://www.emrap.org/episode/lateral/lateral. The video can be accessed for free here: https://www.youtube.com/watch?v=tgQaKVGynFA

### Implementation Methods

Instructors should begin with provision of a brief, 30-minute lecture on OCS (Appendix B: Retrobulbar Hemorrhage, Canthotomy/Cantholysis PowerPoint). We would recommend performing the lecture on a separate day from this simulation to avoid priming the learners regarding the content of the case. Our simulation team separated the lecture and simulation days by one week.

During simulation pre-briefing, all learners should be instructed to conduct “any necessary procedures” on the provided model rather than on the mannikin. They should be encouraged to ask questions during the simulation if they are unclear what anatomical structures the components of the model are intended to mimic.

Instructors may consider evaluating learner LCC skills via the checklist we developed using a modified Delphi method (Appendix C). This may serve as a launch board for discussion during the debriefing, as well as a rubric that residents can take home with them after the simulation to help promote effective studying.

Appropriately completed items should be scored a “1,” and items not completed or not appropriately completed should be scored a “0.” No partial credit should be awarded. The maximum number of points is 19/19 (100%). If only a single instructor is available, one can record learner simulation sessions and fill out the checklist in a post-hoc manner (ensuring that there is minimal time between the simulation and learner receipt of their checklist-based performance). If more than one instructor is available, then having one instructor run the simulation case while the second instructor fills out the checklist would be the most efficient use of time and resources. This will also provide learners with more immediate feedback.

Instructors may also consider evaluating learner knowledge via MCQ test (Appendix D: MCQ test for assessment before and/or after participation in the OCS simulation case) before and/or after participation in the OCS simulation case. Each question is worth 10 points for a total of 100 points. Multiple choice question data may be used to help instructors evaluate whether there is a change in learner medical knowledge before versus after participation in the simulated case. Instructors may consider administering the MCQ again at later timepoints to determine whether there is retention of learner knowledge on the topic of LCC over time. Determining when MCQ performance starts to decline may help instructors determine the necessary frequency of spaced repetition simulations on this topic to ensure retention of learner knowledge necessary for clinical practice.

Finally, instructors may wish to consider evaluating learner attitudes before and after participation in the simulated OCS case (Appendix E: Self-efficacy scale [SES]). The SES is scored using a Likert scale ranging from 1–5, in which “very uncomfortable” correlates with 1 point, and “very comfortable” correlates with 5 points. Learners should be instructed: “Please rate your current level of comfort [BEFORE OR AFTER] our simulation with each of the following items in the setting of a chief complaint of eye pain and vision loss. Place a check mark or X in the box that applies to you.” Items in italics were included in our SES but are not specific to the OCS case and may be excluded at the discretion of the instructor. The SES data may be used by the instructors for quality improvement purposes during future iterations of the simulation case.

We recommend having the LCC model set up and ready prior to the simulation day. The model takes approximately 5 minutes to reset between learners. The case should take approximately 15 minutes to run, and instructors should plan to utilize 15–20 minutes for debriefing after the case. If checklist-based performance is assessed in real time, we suggest going through the checklist with the learner(s) during debriefing.

The case itself utilizes a low-fidelity mannikin, with the voice of the patient played by an embedded actor (i.e., the instructor). We suggest printing out Stimulus #1 and taping it to the mannikin. Supplies needed for ophthalmologic examination should be immediately available to the learner and do not need to be hidden from view. The LCC model and all supplies needed for the LCC procedure should be immediately available. We suggest covering the LCC model and supplies with a sheet to avoid priming the learners that they will need to perform this procedure. Vital signs may be shown on a monitor or verbalized to the learners depending on availability of resources. The instructor should have a digital device such as a smartphone or laptop available to provide the learner with Stimuli #2-5 if/when they are requested (see Appendix F: Stimuli for Simulation Case). No laboratory results are necessary for appropriate diagnosis and management of the patient, and learners should be told that the labs have not resulted if they are requested at any point during the case. In addition to the voice of the patient, the primary instructor may play the roles of consultants and other healthcare professionals (such as the nurse). If additional instructors are available, these roles may be divided up.

### List of items required to replicate this innovation

Please note that unless indicated otherwise, all items listed can be purchased on Amazon.com. See “Appendix A: Lateral Canthotomy/Cantholysis Model: How to Guide” for list of all materials with cost and links.

#### Supplies for ophthalmologic examination

1. Fluorescein
2. Topical ocular anesthetic (such as proparacaine or tetracaine)
3. Paper clip X 2 (to serve as makeshift eyelid retractors)
4. Pen light
5. Snellen chart

#### Supplies for LCC

1. Anesthetic for infiltration (ideally lidocaine with epinephrine)
2. 18-gauge needle
3. 27-gauge needle
4. 10 cc syringe
5. Hemostat
6. Iris scissors

#### High-fidelity LCC model

1. Foam disc (to serve as a base)
2. Foam head
3. Hot glue gun
4. Hot glue sticks
5. Kabob skewers
6. Plastic eyes
7. Rubber bands
8. Scissors
9. Sewing pins
10. Silk tape
11. Tensoplast™ elastic adhesive bandage

### Detailed methods to construct this innovation

Please see “Appendix A: Lateral Canthotomy/Cantholysis Model: How to Guide” for step-by-step instructions with associated images demonstrating each step for clarity.

### Step-By-Step LCC Model Construction Guide

1. Scoop out “orbits” in the foam head using a spoon
2. Insert plastic eyes
3. Hot glue around edges of plastic eyes to hold in place
4. Cut rubber band into 4 pieces, then cut slit down middle to make a “Y”
5. Insert sewing pin through stem of rubber band “Y”
6. Insert sewing pin 2 cm lateral to the lateral canthus
7. Hot glue limbs of rubber band “Y” to the plastic eye
8. Fold Tensoplast™ and cut semi circle to form inner layer of “eyelid,” then apply over plastic eye
  - Fold Tensoplast™ into a square
  - Cut square from roll
  - Fold square in half
  - Cut semicircle
  - Unfold Tensoplast™
  - Rest Tensoplast™ over eye
9. Apply silk tape over Tensoplast™ to form outer layer of eyelid
10. Attach foam head to foam base using kabob skewer
  - Cut kabob skewer into three pieces using scissors
  - Insert skewer pieces into base of foam head
  - Apply foam head to foam base

### LCC Model Turnover Between Cases

1. Turn on hot glue gun
2. Remove cut tape, Tensoplast,™ and rubber band “Y”
3. Insert sewing pin through a new rubber band “Y”
4. Hot glue limbs of rubber band “Y” to the plastic eye
5. Apply new Tensoplast™ and silk tape

### Results and tips for successful implementation

#### Study Implementation/Research Methods

We conducted a single-center prospective pre- and post-interventional study evaluating the impact of an educational intervention on EM resident management of a simulated case of OCS. Our two-part study intervention consisted of a lecture on orbital compartment syndrome (OCS) followed by a four and a half hour OED. Residents were evaluated using self-efficacy scales (SES), multiple-choice questions (MCQ), and a performance checklist at three timepoints: Pre-intervention (pre-OED), immediate post-intervention (post-OED), and three months post-intervention (3M post-OED). The Institutional Review Board (IRB) waived the need for consent from residents (IRB #1216) due to the educational nature of the study. Residents who did not consent to data collection for study purposes were still permitted to participate in the OED for educational purposes.

The checklist was developed and validated using a modified Delphi process for performance of emergent LCC. The original checklist was derived by one of the study team members using common knowledge of best practice from several EM textbooks.^5^–^7^ Feedback was elicited from an ophthalmologist, who was a member of the study team, and revisions were made. Snowball sampling strategy was employed to recruit additional input from EM physicians and ophthalmologists at four academic institutions and one community institution. A total of 22 physicians outside of our study team were contacted via email. Ultimately, 12 board-certified expert physicians (seven EM and five ophthalmologists) from five institutions provided feedback and modification recommendations to the checklist.

The validity of the MCQ test was based on expert consensus among the study authors, who utilized a combination of clinical experience and consultation of peer-reviewed literature for question development. Reliability was ensured via homogenous administration by administering the test in the same fashion at each timepoint. The validity and reliability of self-assessment via Likert scales has been previously validated.^8^

**Figure 1:**
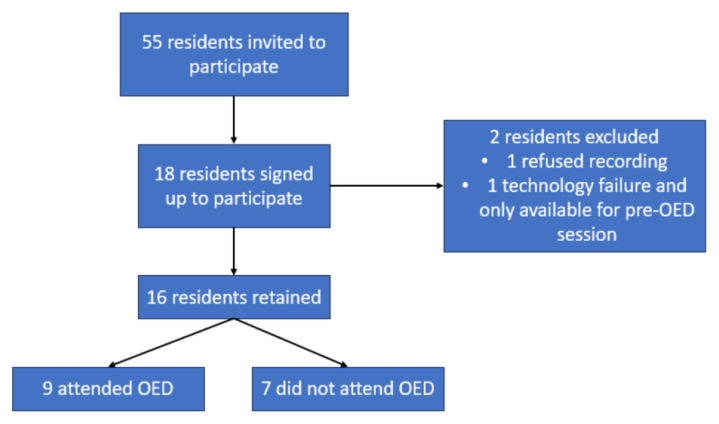
Study flow diagram.

Pre-OED sessions took place in Quarter 1 of 2023. At the beginning of each session, participants completed SES and an MCQ test. The resident completed the OCS case in which one study administrator played an embedded nurse. A Laerdal SimMan 3G™ (model number 212-02150, Stavanger, Norway) was used to simulate the patient. Residents were provided with all equipment that they could require for appropriate ophthalmologic examination as well as performance of a LCC. No debriefing was performed, and residents were informed that debriefing would occur during the OED. Participants then completed the same SES and MCQ test. A total of 18 residents participated in the pre-OED simulation sessions, and 16 residents had sufficient data sets for analysis (Fig. 1).

Sessions were recorded for checklist review. Two study team members who were not involved in administration of the OCS case virtually reviewed and scored the residents using the LCC checklist. Scored checklists were stored on an encrypted server. This prospective study was conducted in a simulation space used for training EM residents at an Urban Level 1 trauma center. The first component of our two-part study intervention took place one week before the OED during regularly scheduled didactic time and consisted of a 30-minute lecture discussing the diagnosis and management of OCS. The structure of the lecture was guided by our LCC checklist, but the checklist was not revealed to residents.

The second component of our two-part study intervention took place on 2/14/2023, consisting of a four and a half hour OED. This began with a 30-minute lecture from one of our study team members discussing systematic ophthalmologic examination with the slit lamp and slit lamp logistics. Residents were equally divided and rotated through eight stations (four procedural stations, three scenario-based simulations, and one table-top session of “Can’t-Miss” eye diagnoses) for approximately 25 minutes each. LCC was one of the procedural stations, and the OCS case was one of the simulations. A total of 25 residents participated in the OED, and 12 faculty members assisted during the event (ten EM and two ophthalmologists).

At the LCC station, the procedural steps and considerations were reviewed, but residents did not receive a copy of the checklist or its contents during the study. The OCS case was debriefed, highlighting the importance of the checklist items. Residents were not exposed to a copy of the checklist at any point during the study.

In the subsequent week, post-OED simulation sessions were conducted using the same methods as the pre-OED simulation session. Three months after the OED, the 3M post-OED simulation sessions were conducted using the same methods as the pre- and post-OED simulation sessions.

We anticipated at least a 30% improvement in the median checklist, MCQ, and SES scores between the pre-OED and post-OED timepoints. Using an anticipated mean score increase of 30%, with a continuous endpoint, alpha of 0.05, and power of 80%, the sample size necessary was calculated to be at least 14 residents.

When scoring checklists, residents were scored in a binary fashion (completed or not completed). If the action was incomplete or done incorrectly, no partial points were awarded. The maximum total score was 19 points out of 19 items. When scoring MCQ tests, each question was awarded 10 points, for a total score of 100. The percentage correct was calculated by dividing the number of points received by 100. For example, three incorrect questions were awarded 70 points.

The SES were scored using a Likert scale ranging from 1–5, in which “very uncomfortable” correlated with 1 point and “very comfortable” correlated with 5 points. The number of points scored by the resident was divided by the total availability points to yield the percentage received. We evaluated for statistically significant change on the checklist-based performance of the OCS case which necessitated performance of a lateral canthotomy and cantholysis. The median checklist scores of all residents were evaluated at each timepoint.

We report categorical variables using frequencies and percentages and continuous variables as medians (min-max). Differences between pre- and post-scores were estimated using the Mann Whitney U test with p < 0.05 considered statistically significant. Kappa coefficients were calculated to assess the interrater reliability (IRR) of our checklist. GraphPad Prism and Microsoft Excel were utilized for statistical analysis.

#### Results

Initial recruitment consisted of 18 residents (Fig. 1), and 16 resident data points were retained in the study; one resident declined video or paper assessment, and there was one failure of video capture followed by lack of availability to participate in the post-OED sessions. There was a second case of video capture failure during the pre-OED session, but MCQ and SES data were obtained. An additional resident did not attend their scheduled post-OED simulation session, but data were collected at two other timepoints. One resident declined video recording but assented to audio recording, allowing for checklist scoring of their simulation sessions. A total of 16 participants were retained in the study – four PGY-1 (two attended the OED, two did not), nine PGY-2 (six attended the OED, three did not), three PGY-3 (one attended the OED, two did not), and zero PGY-4 residents. Median checklist scores, MCQ scores, and SES scores were calculated using all data available at each timepoint.

**Figure 2:**
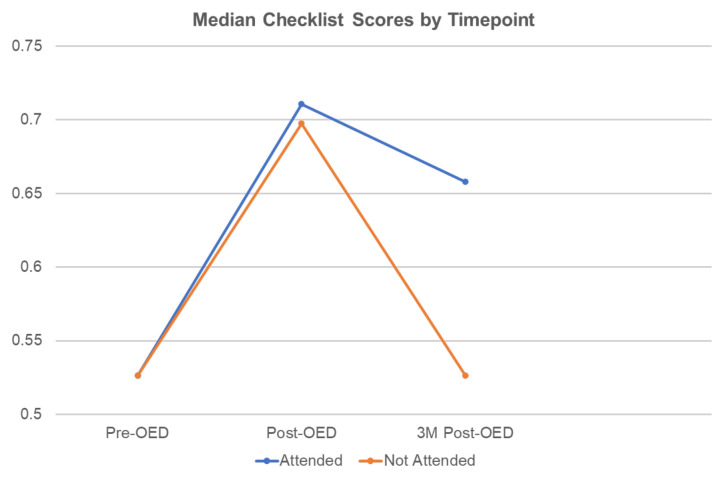
Median checklist scores (range 0 to 1) by time point. The difference between groups at three months was statistically significant.

There was no significant baseline difference between checklist scores for the OED attendees versus non-attendees. Attendees scored higher than non-attendees at the post-OED timepoint (71.0% versus 69.7%, respectively), but this difference was not statistically significant between groups. The median checklist score for attendees at the 3M post-OED was 65.8% compared with 52.6% for non-attendees (13.2% difference), and this difference was statistically significant (Fig. 2). When comparing pre-OED baseline scores with post-OED scores, median checklist scores improved for OED attendees by 18.4%, and for non-attendees by 17.1%, and this difference was statistically significant for both groups.

**Figure 3:**
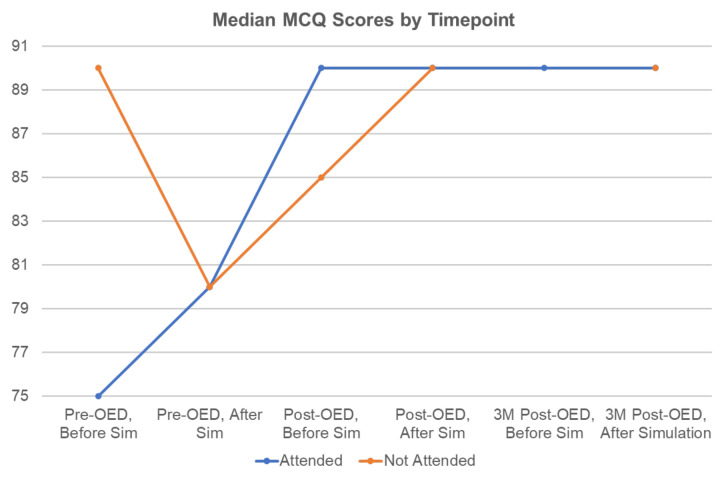
Median MCQ scores (range 0 to 100) by time point. There was no statistically significant difference between groups at any time point.

**Figure 4:**
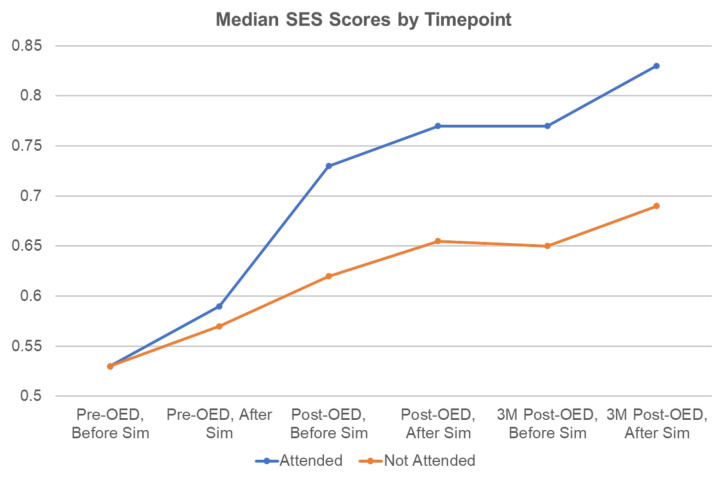
Median SES scores (range 0 to 1) by timepoint. There was no statistically significant difference between groups at any time point.

Baseline MCQ scores differed for attendees versus nonattendees at the pre-OED session; non-attendees scored higher than attendees before the pre-OED simulation, but median scores were the same after the pre-OED session. There was no statistically significant difference in median MCQ score between groups at any time point (Fig. 3). Median SES scores improved over time for both OED attendees and non-attendees, but there was no statistically significant difference between groups at any time point (Fig. 4).

Kappa coefficients were used to determine IRR. Checklist item number 7 demonstrated substantial interrater reliability (IRR). Four checklist items exhibited moderately strong IRR (checklist item numbers 5–6, 10, and 11) and six checklist items exhibited fair IRR (checklist item numbers 1, 12, 14, 16, 18, and 19). OED attendees provided very positive feedback regarding the session, with 100% (10/10) respondents strongly agreeing that the content was applicable to their needs as EPs; 100% (2/2) respondents strongly agreeing that the session was wellorganized; 100% (2/2) strongly agreeing that the content was appropriate for their level of training; 100% (3/3) strongly agreeing that the presenters’ presentation style was effective; 100% (3/3) strongly agreeing that the instructor was effective in creating an interactive and engaging session; 100% (3/3) strongly agreeing that they would be interested in attending future sessions on this topic; and 90% (9/10) globally rating the session as excellent. Selected quotes from OED attendees can be found in Table 1.

#### Discussion

This single-center prospective pre- and post-interventional study of an educational intervention for EM residents demonstrated that the OED consisting of lecture-based, procedural, and simulation-based training improved resident procedural performance as measured via checklist in a simulated case of OCS. The interrater reliability (IRR) for the majority of checklist items was not robust. We suspect that this was related to a single observer training session lasting 30 minutes, and we recommend that more extensive training of observers be conducted during future iterations of this curriculum. Usage of mounted pan-tilt-zoom (PTZ) cameras or live observation could also assist with improving IRR.

Our second research objective was to assess whether there was improvement in skills, knowledge, and attitudes surrounding ophthalmologic examination, diagnosis, and management after our OED intervention. Residents who attended the OED demonstrated significantly increased checklist-based performance at three months compared with those who did not attend, suggesting that attending the OED improved and maintained ophthalmologic diagnostic and management skills. Despite very positive resident feedback regarding the OED, our data do not suggest an improvement in ophthalmologic diagnostic and management knowledge and attitudes for attendees versus non-attendees at any time point. It is surprising that there was no difference in MCQ scores for the two groups given that checklist-based performance differed. However, baseline median MCQ scores for both groups were fairly high, so expecting further improvement after the OED may be unreasonable. It is possible that no difference was noted between attendees versus non-attendees since simulation-based education caters to psychomotor performance, which occupies a higher level of Miller’s pyramid (“shows how” or “does”) than MCQ testing (“knows”).^9^

Overall, our data suggests that further work is needed to determine whether our checklist is both valid and reliable. The fact that median SES scores improved over time for both OED attendees and non-attendees suggests that residents are unaware of how quickly their skills degrade.

#### Limitations and Lessons Learned

There were several limitations of this pre- and postinterventional study. This study was conducted at a single center, and therefore the results would need to be widely externally validated. Personnel limitations required the same study team member to be responsible for running the simulations as well as entering and analyzing the data. This lack of blinding could have introduced bias.

It is unclear why both OED attendees and non-attendees demonstrated similar levels of checklist-based performance at the immediate post-OED simulation session. We were not able to control for which residents attended the resident on orbital compartment syndrome the week before the OED. We could not control for the confounding effect of outside studying that the residents did on their own time or cases that they experienced clinically outside of the study (an additional source of maturation).^10^ The same case was run at the pre-OED, immediate post-OED, and at three months post-OED; thus, it is possible that learning the case itself rather than the content was responsible for improvement in checklist-based performance over time for the OED attendees. There was missing data due to technology failures and inability of one resident to attend their scheduled immediate post-OED session. Despite these limitations, we feel that the rigor of this study’s methodology lends itself to providing reliable and thought-provoking results.

Our results raise the question of whether an ophthalmology “bootcamp” is truly an effective way to deliver ophthalmology skills content to EM residents. It is possible that longitudinal simulation sessions, using cases similar to the OCS case presented in this curriculum, could be used to capitalize on the phenomenon of spaced repetition and ideally promote more long-term learning.^11^

### Associated content

Appendix A: Lateral Canthotomy/Cantholysis Model: How to Guide

Appendix B: Retrobulbar Hemorrhage, Canthotomy/Cantholysis PowerPoint

Appendix C: Lateral Canthotomy/Cantholysis Checklist

Appendix D: MCQ Test and Answers for Assessment Before and/or After Participation in the OCS Simulation Case

Appendix E: Self-Efficacy Scale (SES)

Appendix F: Stimuli for Simulation Case

## Figures and Tables

**Table t3-jetem-9-4-s1:** 

#1	Patient “eye” - should be printed and taped onto the mannikin.
#2	Patient electrocardiogram (ECG) showing sinus tachycardia.
#3	Computed tomography (CT) brain demonstrating no intracranial hemorrhage (ICH), obtained before sign-out. *The previous team did not note the retrobulbar hematoma on the left. The final radiology interpretation should NOT be provided to the learner unless they specifically request it.*
#4	Normal CT cervical spine, obtained before sign-out.
#5	Normal chest x-ray, obtained before sign-out.

**Table t4-jetem-9-4-s1:** 

**Vitals:**	Heart Rate (HR) 112 beats/minute	Blood Pressure (BP) 126/82 mmHg
	Respiratory Rate (RR) 15 breaths/minute	Temperature (T) 37.1°C
	Oxygen Saturation (O_2_Sat) 96% on room air	

**Figure f5-jetem-9-4-s1:**
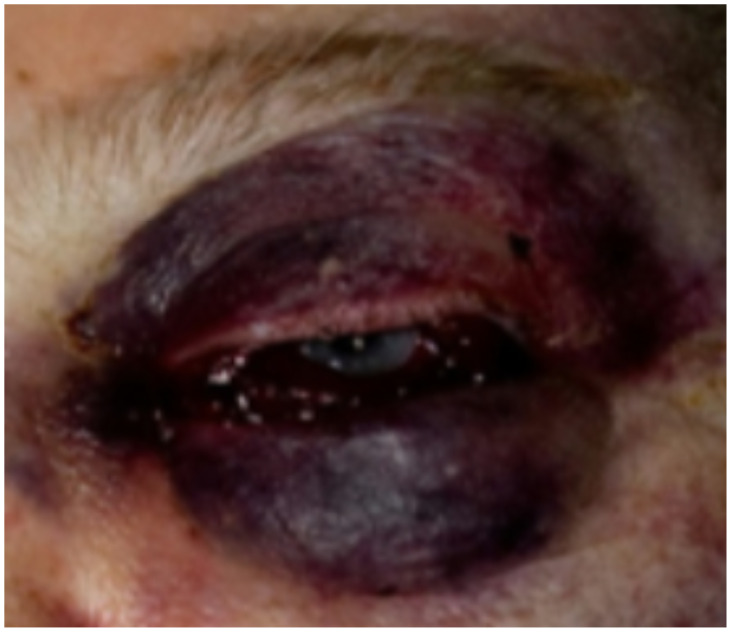


**Figure f6-jetem-9-4-s1:**
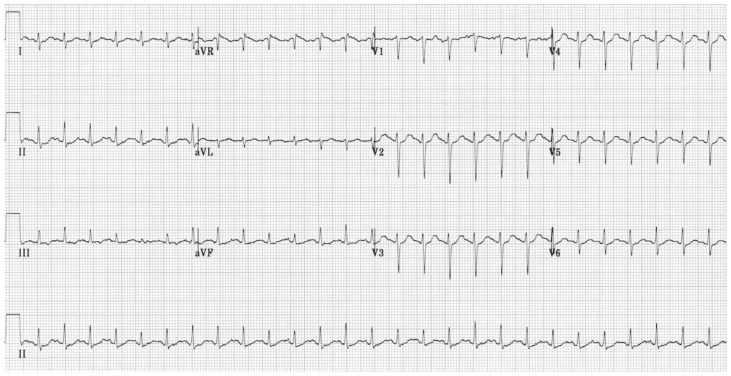


**Figure f7-jetem-9-4-s1:**
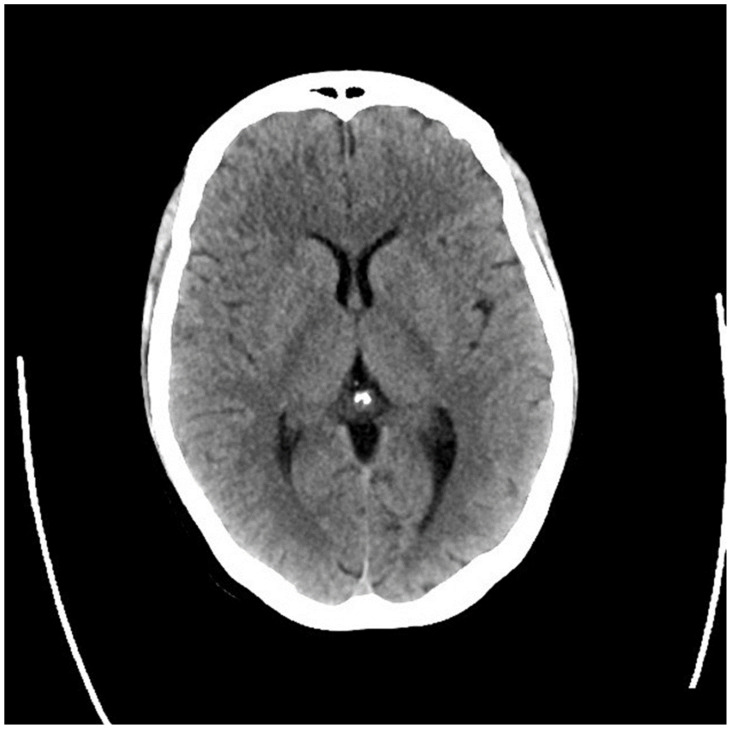


**Figure f8-jetem-9-4-s1:**
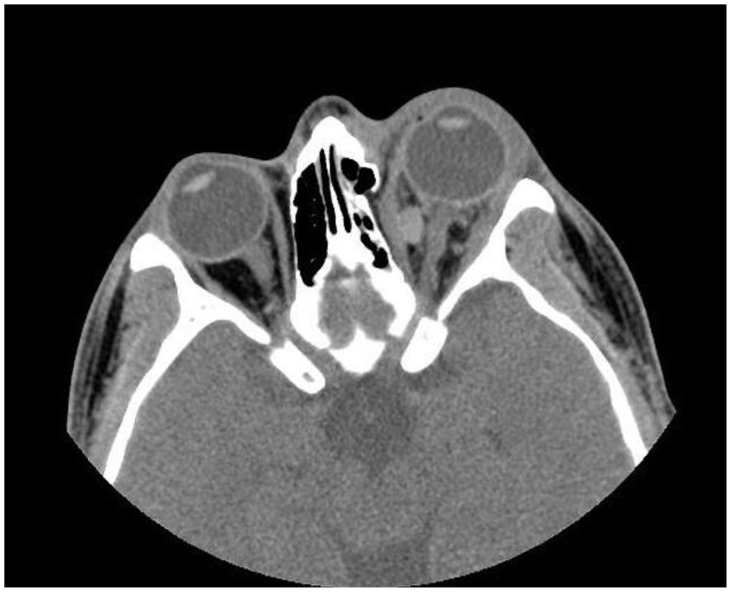


**Figure f9-jetem-9-4-s1:**
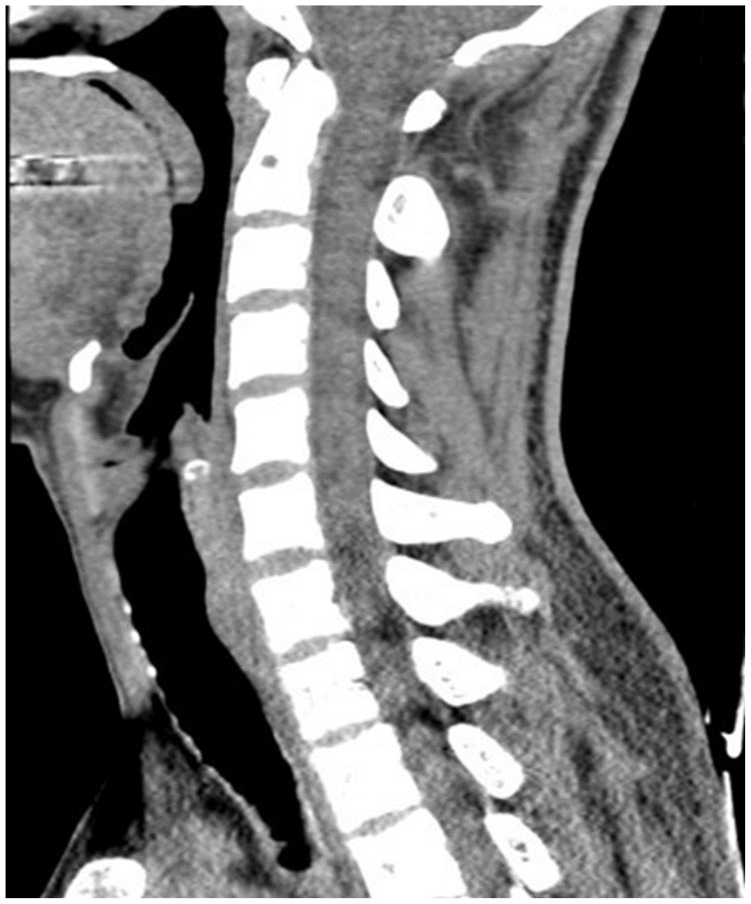


**Figure f10-jetem-9-4-s1:**
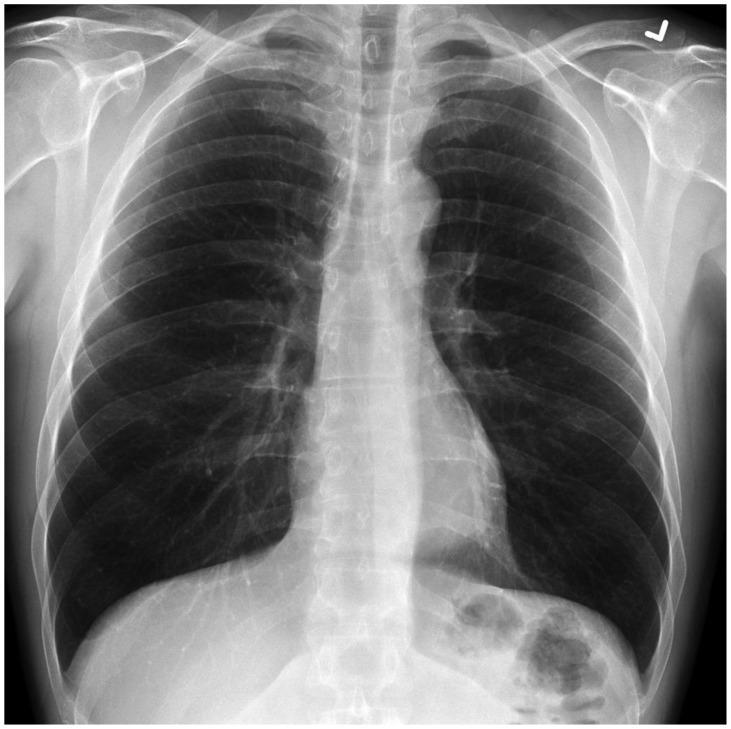


**Table t5-jetem-9-4-s1:** SIMULATION EVENTS TABLE:

Minute (state)	Participant action/trigger	Patient status (simulator response) & operator prompts	Monitor display (vital signs)
0:00 (Baseline state)	Diagnoses OCS (as indicated by visual loss, APD, and elevated IOP OS)	- Patient is agitated, confused, intoxicated, and yelling about eye pain/inability to see OS - Pain does NOT improve with application of fluorescein - Pain slightly improves with intravenous (IV) opioids or ketamine - If learner chooses to send the patient for CT orbit before proceeding with LCC, ED attending (played by voice of instructor) arrives and recommends they do otherwise - If learner chooses to wait for ophthalmology to perform LCC, ophthalmology will instruct them to proceed with the procedure immediately	T 37.1 C HR 112 bpm BP 126/82 mmHg RR 15 bpm O2 96% RA GCS 12
10:00 (LCC)	Cuts the inferior crus of the lateral canthal tendon	- Patient continues to scream about his pain - No improvement in visual loss, APD, or elevated IOP OS	T 37.1 C HR 96 bpm (if given IV pain control) BP 130/80 mmHg RR 16 bpm O2 98% RA GCS 12
14:00 (LCC)	Cuts the superior crus of the lateral canthal tendon	- Patient’s pain improves - Visual acuity: can appreciate light and hand motion OS - APD: resolved OS - IOP: 25 mmHg OS	T 37.1 C HR 85 bpm BP 115/70 mmHg RR 16 bpm O2 98% RA GCS 12
15:00 (Case Completion)	Has consulted ophthalmology	- Unchanged	T 37.1 C HR 85 bpm BP 115/70 mmHg RR 16 bpm O2 98% RA GCS 12

**Table t6-jetem-9-4-s1:** 

**Critical Actions:** Rules out OGI before checking IOP Appropriately diagnoses OCS Cuts inferior crus of lateral canthal tendon first, then *reassesses* (1) visual acuity, (2) pupils, and (3) IOP Cuts superior crus of lateral canthal tendon second, then *reassesses* (1) visual acuity, (2) pupils, and (3) IOP Consults ophthalmology (this may take place at *any point* during the case)	0:00

**Table t7-jetem-9-4-s1:** 

	Milestone	Did not achieve level 1	Level 1	Level 2	Level 3
1	**Emergency Stabilization (PC1)**	□ Did not achieve Level 1	□ Recognizes abnormal vital signs	□ Recognizes an unstable patient, requiring intervention Performs primary assessment Discerns data to formulate a diagnostic impression/plan	□ Manages and prioritizes critical actions in a critically ill patient Reassesses after implementing a stabilizing intervention
2	**Performance of focused history and physical (PC2)**	□ Did not achieve Level 1	□ Performs a reliable, comprehensive history and physical exam	□ Performs and communicates a focused history and physical exam based on chief complaint and urgent issues	□ Prioritizes essential components of history and physical exam given dynamic circumstances
3	**Diagnostic studies (PC3)**	□ Did not achieve Level 1	□ Determines the necessity of diagnostic studies	□ Orders appropriate diagnostic studies. Performs appropriate bedside diagnostic studies/procedures	□ Prioritizes essential testing Interprets results of diagnostic studies Reviews risks, benefits, contraindications, and alternatives to a diagnostic study or procedure
4	**Diagnosis (PC4)**	□ Did not achieve Level 1	□ Considers a list of potential diagnoses	□ Considers an appropriate list of potential diagnosis May or may not make correct diagnosis	□ Makes the appropriate diagnosis Considers other potential diagnoses, avoiding premature closure
5	**Pharmacotherapy (PC5)**	□ Did not achieve Level 1	□ Asks patient for drug allergies	□ Selects an medication for therapeutic intervention, consider potential adverse effects	□ Selects the most appropriate medication and understands mechanism of action, effect, and potential side effects Considers and recognizes drug-drug interactions
6	**Observation and reassessment (PC6)**	□ Did not achieve Level 1	□ Reevaluates patient at least one time during case	□ Reevaluates patient after most therapeutic interventions	□ Consistently evaluates the effectiveness of therapies at appropriate intervals
7	**Disposition (PC7)**	□ Did not achieve Level 1	□ Appropriately selects whether to admit or discharge the patient	□ Appropriately selects whether to admit or discharge Involves the expertise of some of the appropriate specialists	□ Educates the patient appropriately about their disposition Assigns patient to an appropriate level of care (ICU/Tele/Floor) Involves expertise of all appropriate specialists
9	**General Approach to Procedures (PC9)**	□ Did not achieve Level 1	□ Identifies pertinent anatomy and physiology for a procedure Uses appropriate Universal Precautions	□ Obtains informed consent Knows indications, contraindications, anatomic landmarks, equipment, anesthetic and procedural technique, and potential complications for common ED procedures	□ Determines a back-up strategy if initial attempts are unsuccessful Correctly interprets results of diagnostic procedure
20	**Professional Values (PROF1)**	□ Did not achieve Level 1	□ Demonstrates caring, honest behavior	□ Exhibits compassion, respect, sensitivity and responsiveness	□ Develops alternative care plans when patients’ personal beliefs and decisions preclude standard care
22	**Patient centered communication (ICS1)**	□ Did not achieve level 1	□ Establishes rapport and demonstrates empathy to patient (and family) Listens effectively	□ Elicits patient’s reason for seeking health care	□ Manages patient expectations in a manner that minimizes potential for stress, conflict, and misunderstanding. Effectively communicates with vulnerable populations, (at risk patients and families)
23	**Team management (ICS2)**	□ Did not achieve level 1	□ Recognizes other members of the patient care team during case (nurse, techs)	□ Communicates pertinent information to other healthcare colleagues	□ Communicates a clear, succinct, and appropriate handoff with specialists and other colleagues Communicates effectively with ancillary staff

**Table 1:** Selected quotes from OED attendees.

“Session was tailored well to EM needs.”
“Very useful skills.”
“This was exactly what I needed. Probably the best conference this year. Thank you.”

## INSTRUCTOR MATERIALS

**Case Title:** A Case of Painful Visual Loss

**Case Description & Diagnosis (short synopsis):** A 41-year-old male presents to the emergency department (ED) with painful post-traumatic unilateral vision loss. Residents are expected to identify clinical orbital compartment syndrome (OCS), appropriately conduct tonometry to assess intraocular pressure (IOP), and perform lateral canthotomy and cantholysis (LCC) proficiently prior to obtaining imaging or further testing.

**Equipment or Props Needed:**

- Mannikin (which does not need to be high-fidelity)
- Supplies for ophthalmologic examination
  ○ Fluorescein and topical ocular anesthetic (such as proparacaine or tetracaine)
  ○ Paper clip X 2 (to serve as makeshift eyelid retractors)
  ○ Pen light
  ○ Snellen chart
  ○ Woods lamp
- Supplies for LCC
  ○ Anesthetic for infiltration (ideally lidocaine with epinephrine) with 18- and 27- gauge needles, 10 cc syringe
  ○ Hemostat
  ○ High-fidelity LCC model (see separate instructions)
  ○ Iris scissors

**Actors needed:**

None. Instructor may play the voice of the patient and the voice of any consultants (i.e., ophthalmology) as well as the nurse. Separate individuals may be used for these roles if more facilitators are available.

**Stimulus Inventory:**

Learners should only need stimuli #1 and stimuli #2. However, if they question the diagnoses that their colleagues made prior to sign-out and request imaging results, they may be provided with the remaining stimuli. If learners request laboratory results, they should be told that these have not resulted yet, as no laboratory results are needed for learners to make the diagnosis and manage the patient appropriately.[Table t3-jetem-9-4-s1]

**Background and brief information:** Triage note: Brought in by police after assault at a bar. Patient says his left eye hurts and he “can’t see.”

Sign out from co-resident on previous ED shift:

- Punched in the left eye at a bar
- Already underwent primary and secondary surveys
- CT head/cervical spine look negative per their read; radiology interpretations pending
- They didn’t get a chance to examine the left eye yet since the patient went straight from the trauma bay to the CT scanner
- Still intoxicated and belligerent
- Disposition: Likely discharge when sober

**Initial presentation:** On examination by the learner after receiving “sign-out,” the patient is intoxicated and belligerent. He can answer yes and no questions and frequently yells about how painful his left eye is.

**How the scene unfolds:** Learners are expected to conduct a brief but thorough ophthalmologic examination, which will allow them to appropriately diagnose OCS. The patient will require pain control to facilitate examination by the learner. The learner will be unable to retract the eyelids manually and will need to use folded paper clips if they do not have eyelid retractors available to them (if they do not know how to do this, the “embedded nurse” will say, “I’ve seen other docs do it this way” and will assist them). Essential components of the ophthalmologic examination that will allow the learner to make the diagnosis of OCS include:

1. Presence of proptosis on gross examination, along with absence of findings on gross examination of the eye that would suggest open globe injury (OGI) (including scleral defects, corneal defects, or abnormal pupil shape)
2. Decreased visual acuity in the left eye compared to the right
3. Absence of an extraocular muscle (EOM) entrapment syndrome that would explain the decreased visual acuity on the left
4. Presence of an afferent pupillary defect (APD) on the left
5. Negative Seidel test on the left via fluorescein stain
6. Elevated IOP on the left compared with the right

Once the diagnosis of OCS is made, the learner should page ophthalmology and proceed with preparation for LCC. Ophthalmology will be 30 minutes away, which should force the learner to proceed with LCC. The patient will not be able to give consent given that he is intoxicated, but he should be given an explanation of what needs to happen and receive appropriate pain medication/sedation to facilitate the procedure. Learners must reassess the (1) visual acuity, (2) pupils, and (3) IOP after cutting the inferior crus of the lateral canthal tendon. These examination findings will not improve, which should force the learner to proceed with cutting the superior crus of the lateral canthal tendon. When reassessed, the (1) visual acuity, (2) pupils, and (3) IOP will all improve after cutting the superior crus.

If the learner is unsure how to proceed after the inferior crus has been cut and the examination has not improved, they will be permitted to speak with the ophthalmology consultant via phone. The case ends when the superior crus has been successfully cut.

**Critical actions:**

1. Rules out OGI before checking IOP
2. Appropriately diagnoses OCS
3. Cuts inferior crus of lateral canthal tendon first, then *reassesses* (1) visual acuity, (2) pupils, and (3) IOP
4. Cuts superior crus of lateral canthal tendon second, then *reassesses* (1) visual acuity, (2) pupils, and (3) IOP
5. Consults ophthalmology (this may take place at *any point* during the case)

**Case Title:** A Case of Painful Visual Loss

**Chief Complaint:** Left eye pain[Table t4-jetem-9-4-s1]

**General Appearance:** Well-dressed and well-nourished tall, thin male but disheveled from physical altercation. Smells of alcohol

**Primary Survey:**

- **Airway:** Intact; awake and speaking
- **Breathing:** No obvious respiratory distress
- **Circulation:** Mildly tachycardic, 2+ peripheral pulses, capillary refill < 2 seconds

**History:**

- **History of present illness (HPI) (provided by colleague during sign-out):** Punched in the left eye at a bar
  ○ Already underwent primary and secondary surveys
  ○ CT head/cervical spine look negative per their read; radiology interpretations pending
  ○ They didn’t get a chance to examine the left eye yet since the patient went straight from the trauma bay to the CT scanner
  ○ Still intoxicated and belligerent
  ○ Disposition: Likely discharge when sober
- **HPI (will not be volunteered but may be provided by patient if learner asks):**
  ○ Punched in the left eye at a bar
  ○ Did not fall or lose consciousness
  ○ Doesn’t remember how many drinks he had
  ○ “My eye f*@$% hurts!”
- **Past medical history:** Tobacco dependence
- **Past surgical history:** None
- **Patient’s medications:** Nicotine patches
- **Allergies:** None
- **Social history:** Binge drinks on weekends, snorts cocaine occasionally
- **Family history:** N/A

**Secondary Survey/Physical Examination:**

- **General appearance:** Well-dressed and well-nourished tall, thin male but disheveled from physical altercation. Smells of alcohol
- **HEENT:**
  ○ **Head:** within normal limits (WNL)
  ○ **Eyes:** learner will need to open the right eye with fingers since the patient will not follow commands, and will need to open the left eye using commercial eyelid retractors or two paper clips
    ▪ Visual acuity – 20/20 right (OD), no light perception left (OS)
    ▪ Confrontational visual fields (CVF) – intact OD, unable to test OS
    ▪ Anterior Segment – learner should NOT proceed with slit lamp exam, as it will produce an unnecessary delay in performing a vision-saving procedure. If they request a slit lamp, the nurse should tell them that it is currently unavailable. The findings below are noted on gross examination of the eye
      - External structures and adnexa – proptosis OS
      - Lids and lashes – swelling and ecchymosis of the lids OS
      - Conjunctiva and sclera – 360-degree subconjunctival hemorrhage with no scleral defects OS
      - Cornea – no irregularities bilaterally (OU)
      - Anterior chamber – no visible hyphema OS
      - Iris and pupils – pupil and iris demonstrate no shape irregularities OS
      - Pupils – slow to react and relative APD OS
  ○ EOM – intact OU
  ○ IOP – 16 OD, 45 OS
  ○ Fundus – N/A
  ○ Fluorescein – no corneal abrasions OU
- **Ears:** WNL
- **Nose:** WNL
- **Throat:** WNL
- **Neck:** WNL
- **Heart:** Tachycardic but regular rhythm. No murmurs, rubs, or gallops.
- **Lungs:** WNL
- **Abdominal/GI:** WNL
- **Genitourinary:** WNL
- **Rectal:** WNL
- **Extremities:** WNL
- **Back:** WNL
- **Neuro:** Refuses to open his eyes. Oriented only to self and repeatedly yelling, “My eye! *!&% I can’t see!” Does not follow commands but withdraws to pain. CN IV-XII intact. Moving all four limbs. Sensation grossly intact
- **Skin:** WNL with exception of left eyelid - see eye examination
- **Lymph:** WNL
- **Psych:** Agitated but intermittently redirectable

*Patient “eye” - should be printed and taped onto the mannikin.*

Dr. Steven Couch. The patient in this image provided consent to use these images.[Fig f5-jetem-9-4-s1]

*Patient electrocardiogram (ECG) showing sinus tachycardia.*

ECG Sinus Tachycardia. In: Life in the Fast Lane. https://litfl.com/wp-content/uploads/2018/08/ECG-Sinus-Tachycardia.jpg. Accessed June 5, 2024. CC BY-NC-SA 4.0[Fig f6-jetem-9-4-s1]

*Computed tomography (CT) brain demonstrating no intracranial hemorrhage (ICH), obtained before sign-out. The previous team did not note the retrobulbar hematoma on the left. The final radiology interpretation should NOT be provided to the learner unless they specifically request it.*

Gaillard F. Normal CT brain (pre and post contrast). In: Radiopaedia. https://doi.org/10.53347/rID-37008. Accessed June 5, 2024. CC BY-NC-SA 3.0[Fig f7-jetem-9-4-s1]

Sharifkashani S. Orbital Blow-out fracture and retrobulbar hemorrhage. In: Radiopaedia. https://doi.org/10.53347/rID-99591. Accessed June 5. 2024. CC BY-NC-SA 3.0[Fig f8-jetem-9-4-s1]

*Normal CT cervical spine, obtained before sign-out.*

Source: Di Muzio B. Normal cervical spine - CT. In: Radiopaedia. https://doi.org/10.53347/rID-39801. Accessed June 5, 2024. CC BY-NC-SA 3.0[Fig f9-jetem-9-4-s1]

*Normal chest x-ray, obtained before sign-out.*

Source: Gaillard F. Normal chest x-ray. In: Radiopaedia. https://doi.org/10.53347/rID-8304. Accessed June 5, 2024. CC BY-NC-SA 3.0[Fig f10-jetem-9-4-s1]

## OPERATOR MATERIALS

[Table t5-jetem-9-4-s1]

**Diagnosis:**

OCS

**Disposition:**

Remains in ED due to intoxication, and ophthalmology is consulted.

## DEBRIEFING AND EVALUATION PEARLS

### Orbital Compartment Syndrome and Lateral Canthotomy

The orbit is a tightly enclosed space and has limited room for expansion when there is an increase in the volume of its contents. When increased volume is introduced via bleeding (such as in traumatic or iatrogenic retrobulbar hematoma), mass lesion, or infection, the pressure within the space increases. This increased intra-orbital (and intraocular) pressure leads to compression and subsequent ischemia of the optic nerve, which can produce vision loss. OCS is a clinical diagnosis. Presenting symptoms include vision loss with or without diplopia associated with proptosis and an explanatory mechanism (in the case of trauma, such as with our patient, a punch to the eye could easily explain retrobulbar hematoma). Particularly high suspicion should be held in patients on anticoagulation or other risks for bleeding. Classic physical examination findings include:

1. Absence of light perception
2. APD
3. Elevated IOP (> 21 mmHg)
4. Chemosis
5. Proptosis with swelling of the eyelids
6. 360-degree subconjunctival hemorrhage^12^

***The most important findings are numbers 1–3. Remember that you must rule out an OGI before checking IOP or performing LCC, as you can extrude intraocular contents by putting pressure on the eye!*** *13*

The patient with OCS may have abnormal EOM, particularly if there is associated extraocular muscle entrapment from orbital floor fracture.^12^,^14^ Lateral canthotomy should not be delayed for more than two hours, as more than 90–120 minutes of retinal ischemia puts the patient at risk for permanent vision loss.^15^

Lateral canthotomy is a high-acuity, low-opportunity procedure, but the emergency physician must be prepared to perform this immediately if OCS is present and the patient’s vision is at stake. Key components to avoid missing include:

1. Using sterile technique
2. Providing pain control (likely with IV opioids or ketamine) and properly anesthetizing the patient (by injecting lidocaine with epinephrine into the lateral canthus)
3. Clamping the lateral canthus to ensure hemostasis
4. Cutting the inferior crus of the lateral canthal tendon first, then reassessing (1) visual acuity, (2) pupils, and (3) IOP
5. Moving to the superior crus of the lateral canthal tendon, then reassessing (1) visual acuity, (2) pupils, and (3) IOP
6. Consulting ophthalmology (at any point during this process)^16^

This case highlighted the importance of conducting a re-evaluation of patients during sign out and avoiding anchoring bias. Anchoring bias refers to our tendency as humans to stick with the initial impression, even if new information presents itself that should prove that impression to be incorrect.^17^ In addition, as part of a medical team, we have an innate desire to trust our colleagues’ judgment. We may want not to say this, but it is also easier to “go along” with someone else’s train of thought than to “reinvent the wheel” and start over again with the history and physical exam. Unfortunately, anchoring bias is one of the most common cognitive errors that can lead to diagnostic errors.^18^ Methods for avoiding anchoring bias in our practice include awareness of cognitive biases, ensuring we run through a thorough differential diagnosis as well as going back to the drawing board if new signs or symptoms arise that don’t fit with the presumed working diagnosis.^17^,^18^

Regarding the differential, using a cognitive organizing strategy to help us formulate a list of possibilities that includes the most serious or life-threatening diagnosis (such as “SPIT” - serious, probable, interesting, treatable)^19^ will help us to avoid missing can’t-miss diagnoses. There are numerous systems-based interventions that have been proposed to intervene on anchoring bias, such as integrated diagnostic support pop-ups built into electronic medical records.^18^ In summary, a multimodal approach is necessary to counteract our human instinct to default to the initial impression, which may not always be the correct one.

**Other debriefing points:**

- Debriefing points for the ophthalmologic examination
  ○ If the learner only examined the affected eye, emphasize the importance of comparing both eyes (as they will need to know the visual acuity, EOM status, pupillary exam, and IOP for the unaffected eye for comparison)
  ○ Discuss any key missing components of the exam. The most important points are:
    ▪ Rule out OGI before checking IOP
    ▪ At minimum, you need a (1) visual acuity, (2) pupillary exam (including checking for an APD), and (3) IOP to make the diagnosis of OCS
- Debriefing points for the LCC
  ○ Return to the model used during the case. Remove the tape “eyelid” and show them whether they successfully cut both the inferior and superior crus
  ○ Discuss that it can be hard to visually identify the inferior and superior crus when the eye is swollen and proptotic. “Strumming” each crus with your scissors should give you a “guitar string” palpatory sensation, allowing you to know that you are cutting the correct structure
  ○ Emphasize the importance of re-examination of (1) visual acuity, (2) pupillary exam (including checking for an APD), and (3) IOP after cutting each crus to know whether the procedure was successful

**Wrap Up:** Learners should be encouraged to watch the EM:RAP lateral canthotomy video (see “Learner responsible content”) after the simulation session if they did not do so already.

## SIMULATION ASSESSMENT

*A Case of Painful Visual Loss - Managing Orbital Compartment Syndrome in the Emergency Department*

Learner: _________________________________________

***Assessment Timeline***

This timeline is to help observers assess their learners. It allows observer to make notes on when learners performed various tasks, which can help guide debriefing discussion.[Table t6-jetem-9-4-s1]

**Critical Actions:**

□ Rules out OGI before checking IOP
□ Appropriately diagnoses OCS
□ Cuts inferior crus of lateral canthal tendon first, then ***reassesses*** (1) visual acuity, (2) pupils, and (3) IOP
□ Cuts superior crus of lateral canthal tendon second, then ***reassesses*** (1) visual acuity, (2) pupils, and (3) IOP
□ Consults ophthalmology (this may take place at ***any point*** during the case)

**Summative and formative comments:**

**Milestones assessment:** [Table t7-jetem-9-4-s1]

## INSTRUCTOR MATERIALS

### Appendix A Lateral Canthotomy/Cantholysis Model: How to Guide

Please see associated PowerPoint file

[Table t8-jetem-9-4-s1]

**Table t8-jetem-9-4-s1:**

Approximate cost of items to create this innovation:		APPROXIMATE COST	PURCHASE LINK
**Supplies for ophthalmologic examination**	Fluorescein	$17.70	https://www.amazon.com/Fluorescein-Strips-Pack-of-100/dp/B0CQW5HL19/ref=sr_1_1?crid=2WX10X29EFGQO&dib=eyJ2IjoiMSJ9.WbXKqDaOtbnZxAIc33y-eFqbXurr64NOgh8ltZEGu0soJUAIH6uwF79ye8bQodFiESmn25GkS36KmcylrM0DHVcDyUdpjULynvOuCywR-eIgvG3tEE2K6eSramzl2GI_txZFy8gEQ_9m
	Topical ocular anesthetic (such as proparacaine or tetracaine)	$12.45	https://pharmacy.amazon.com/Bausch-Health-TETRACAINE-Bottle-Milliliter/dp/B08LTTN76Q?ref_=sr_1_1&keywords=tetracaine&crid=3MUS717KPFOC9&sprefix=tetracain%2Caps%2C184&dib_tag=se&dib=eyJ2IjoiMSJ9.DS4RYEyjM6-THcl2xnVRJ4qKnr6pQKGl5CKs-uugfiXGjHj071QN20LucGBJI
	Paper clip X 2 (to serve as makeshift eyelid retractors)	$4.49	https://www.amazon.com/220pcs-Silver-Medium-Office-Paperclips/dp/B0B5KYWNH4/ref=sr_1_5?crid=29NO7AFMGG6OK&dib=eyJ2IjoiMSJ9.lrD0z74gsCqId6OMlxxzgveoQsVjVc6ixYQYQ4c9TRYv0skVY5fsRJqr-bXNDmAvRjlot-gnhmvuWsLIY8-nIc9h_p0hA6Xmtbd_kuop5WrbA3HUzSws-yQWhZLmF96epGTm
	Pen light	$6.01	https://www.amazon.com/sspa/click?ie=UTF8&spc=MTo0MTIxMjMwMDUxNDEzMjUwOjE3MTgwMzgyMTg6c3BfYXRmOjIwMDA2NjMzOTEyNTg2MTo6MDo6&url=%2FRISEMART-Reusable-Medical-Penlight-Student%2Fdp%2FB08X3D2KP3%2Fref%3Dsr_1_2_sspa%3Fcrid%3D9HGKLGAY0YLZ%26dib%3DeyJ2IjoiMSJ9.n
	Snellen chart	$6.99	https://www.amazon.com/Snellen-Canvas-Non-Reflective-Wooden-Framed/dp/B09DRR4Y9Z/ref=sr_1_1_sspa?crid=V2B698M8IE2C&dib=eyJ2IjoiMSJ9.s3NerLWWaBtIT38EErjDNe7L3DHdO1pZa_2gifmVkKl3q1wAIIHMjS7klwtPXuevg6-k2IZZBpOvrJ9x3gZkjPkBMDseCGaQ16tGloLCdqDKT2nfWPtDlnLWn42
**Supplies for LCC**	Anesthetic for infiltration (ideally lidocaine with epinephrine)	$5.99	https://www.allivet.com/product/lidoject-injection--lidocaine-hcl-2-100-ml/31540.html?gclid=CjwKCAjwyJqzBhBaEiwAWDRJVG_zo0jSH6H8RoypBzLZPRJ5g8sCzBwTQ518VmNEeYQwnsoYzeC4gRoCwYYQAvD_BwE
	18-gauge needle	$8.99	https://www.amazon.com/100Pack-Industrial-Dispensing-Accessories-Individual/dp/B0CQWCS7DM/ref=sr_1_2_sspa?crid=3TODOCR4G4EIG&dib=eyJ2IjoiMSJ9.THhvsiuq-TrBoFiE57wTP13mZAOqUYIy-rSVc6p3pY7WE7zyEgZ2Nligyw3B0uKVV0FhY5JaHIhpHeQ2j-5vzdRcMlGb7aSUHhHg_UKQ5NLyly3Bl
	27-gauge needle	$12.99	https://www.amazon.com/Alimoden-Industrial-Accessories-Refilling-Individually/dp/B0CYSQS8CH/ref=sr_1_2_sspa?crid=2HVUSQY5ODLGA&dib=eyJ2IjoiMSJ9.HL-o0Ysfm01h7z0GstmujDBZpIABCuQ-cygmONKMBYwS75tgoSq-gC97tLDulM3NDjEokavTN_0IcujxIHCKAtcuJJ4HWEr_vjYeDqgfYJRvGA2
	10 cc syringe	$9.99	https://www.amazon.com/100-Syringe-without-Needle-Individually/dp/B0CSZ8DWQ6/ref=sr_1_2_sspa?crid=2KOOE34LSLQ41&dib=eyJ2IjoiMSJ9.ziziTaJPeGvmbOFKKXvmAMP5W53BQ6Chg4Y5ENN0srr-e7QFxkPHyuwfjzbf8ePzQG9dptwjAtTe7ZyHYu88ntZq_Mh94g-r6mx3-HMaNPrRjdmD0rzjEobrBM23mq5eA6ADMhN8_D8RlMxAInqQdRtSq1ff75Uzz62yuutM_yqPUk_pPYIS4pQj2fizssl0mac6Dv8hnJwY3cuvL6W780aGI2Klgd242ERiDFwvo_c.HkLnLcerUSY7FX9ZF16kTC-GACduD0x-Gihtb6ZVkBU&dib_tag=se&keywords=10+cc+syringe+without+needle&qid=1718038113&sprefix=10+cc+sy,aps,171&sr=8-2-spons&sp_csd=d2lkZ2V0TmFtZT1zcF9hdGY&psc=1
	Hemostat	$7.99	https://www.amazon.com/Fishing-Straight-Hemostat-Forceps-Stainless/dp/B088PBNSWR/ref=sr_1_1_sspa?crid=19CWAAG37DIQP&dib=eyJ2IjoiMSJ9.CjqQ8LvzohtuP-S979z0Fxy4bUe-YusxNCDkxvNIFZEvr3Pb8cU1EM2dMrgtQhQYTxpeoQ_zQ2_Xxhdf5vji0OQ0crFMfLXcJvZ20wbx-r-s8uEPS7_ab-UtiJ
	Iris scissors	$7.99	https://www.amazon.com/Cynamed-Dissecting-Precision-Scissors-Straight/dp/B07WCYL655/ref=sr_1_1_sspa?crid=DXW0BETTWASN&dib=eyJ2IjoiMSJ9.WhaA1pkrePXaq4XbrctTjQuyACVrC4-ragU4vnLNtjRXhJUkxZNjrwPBnqL1fdP0Mtz88_X4QwjfoYNk7WisYl-_DYtmoBv6m9LgCjCziWllRO_JTqY5wZkk
**High-fidelity LCC model**	Foam disc (to serve as a base)	$8.25	https://www.amazon.com/Craft-Foam-Circle-Disc-Diameter/dp/B07LBFCMM9/ref=sr_1_1_sspa?crid=37E4NX9GRTB8K&keywords=foam+disc&qid=1705075018&sprefix=foam+disc%2Caps%2C88&sr=8-1-spons&sp_csd=d2lkZ2V0TmFtZT1zcF9hdGY&psc=1
	Foam head	$3.99	https://www.amazon.com/Mannequin-Female-Model-Display-Manikin/dp/B0CJVMMSVK/ref=sr_1_1_sspa?keywords=black%2Bstyrofoam%2Bhead&qid=1705074721&sr=8-1-spons&sp_csd=d2lkZ2V0TmFtZT1zcF9hdGY&th=1
	Hot glue gun	$8.99	https://www.amazon.com/Assark-Sticks-School-Repairs-20W/dp/B09FYWQ44L/ref=sr_1_1_sspa?crid=1X3UI466HB0FW&keywords=hot%2Bglue%2Bgun&qid=1705074746&sprefix=hot%2Bglue%2Caps%2C93&sr=8-1-spons&sp_csd=d2lkZ2V0TmFtZT1zcF9hdGY&th=1
	Hot glue sticks	$7.32	https://www.amazon.com/Surebonder-DT-100-Sticks-All-Temperature-5-Sticks-100/dp/B003JZII34/ref=sr_1_4_sspa?crid=G104O844ITG2&keywords=hot+glue+sticks&qid=1705074775&sprefix=hot+glue+sticks%2Caps%2C90&sr=8-4-spons&sp_csd=d2lkZ2V0TmFtZT1zcF9hdGY&psc=1
	Kabob skewers	$5.99	https://www.amazon.com/Comfy-Package-Bamboo-Appetizers-Grilling/dp/B0931JFMRC/ref=asc_df_B0931JFMRC/?tag=hyprod-20&linkCode=df0&hvadid=563782868078&hvpos=&hvnetw=g&hvrand=11956833589563353484&hvpone=&hvptwo=&hvqmt=&hvdev=c&hvdvcmdl=&hvlocint=&hvlocphy=902
	Plastic eyes	$6.99	https://www.amazon.com/gp/product/B01I53MLYG/ref=ppx_yo_dt_b_search_asin_title?ie=UTF8&psc=1
	Rubber bands	$9.99	https://www.amazon.com/Rubber-Bands-Band-Depot-Pound/dp/B084H2HFN3/ref=sr_1_3_sspa?crid=SHQTPRDC5PNS&keywords=rubberbands&qid=1705074905&sprefix=rubberband%2Caps%2C87&sr=8-3-spons&sp_csd=d2lkZ2V0TmFtZT1zcF9hdGY&psc=1
	Scissors	$3.97	https://www.amazon.com/Scotch-1448-Precision-Scissor-8-Inches/dp/B001BKHHGS/ref=sr_1_1?crid=16CML22TVRZHH&keywords=scissors&qid=1705074923&sprefix=scissors%2Caps%2C86&sr=8-1
	Sewing pins	$5.99	https://www.amazon.com/1000PCS-Straight-Durable-Stainless-Dressmaker/dp/B08FWZNCWJ/ref=sr_1_7?crid=9GVKO07LNO2G&keywords=sewing%2Bpins&qid=1705074850&sprefix=sewing%2Bpin%2Caps%2C86&sr=8-7&th=1
	Silk tape	$9.99	https://www.amazon.com/Conkote-Transparent-Medical-Adhesive-Surgical/dp/B09499CCKT/ref=sr_1_1_sspa?crid=3J1ALHRL0TXQ3&keywords=silk+tape&qid=1705074957&sprefix=silk+tap%2Caps%2C89&sr=8-1-spons&sp_csd=d2lkZ2V0TmFtZT1zcF9hdGY&psc=1
	Tensoplast™ elastic adhesive bandage	$9.99	https://www.amazon.com/Tenoplast-Elastic-Adhesive-Bandage-x5/dp/B000GG5TF4
**TOTAL COST:**		**$183**	

* Please note that the cost of a low-fidelity mannikin was not included because most simulation labs will already have this item in stock. The cost of a Woods lamp was also not included because this can be borrowed from the emergency department for use during the simulation.

### Appendix B Retrobulbar Hemorrhage, Canthotomy/Cantholysis PowerPoint

Please see associated PowerPoint file

### Appendix C Lateral Canthotomy/Cantholysis Checklist

[Table t9-jetem-9-4-s1]

**Table t9-jetem-9-4-s1:**

Item #	Action	Achieved?
1	Examines external eye and surrounding face for concomitant injury/alternate causes of symptomatology	
2	Visual acuity testing in one eye at a time (*must perform the following steps in sequential order as dictated by the individual patient’s exam; full credit should be given if the necessary steps are performed for the patient)* Asks patient whether they wear corrective lenses; note whether these are worn during the visual acuity testing* Tests visual acuity using Snellen chart. If unable, moves on to confrontation Tests visual acuity using confrontation. If unable, moves on to hand motion Tests visual acuity using hand motion. If unable, moves on to light perception Tests visual acuity using light perception	
3	Tests confrontational visual fields (four quadrants of vision) in each eye, one at a time	
4	Assesses extraocular movements	
5	Measures pupils for equal size and reactivity to light	
6	Checks for afferent pupillary defect (APD) via swinging flashlight test	
Rules out OGI and assesses IOP (the following steps must be performed in ***sequential order***):		
7	Performs fluorescein stain to rule out Seidel’s test/OGI BEFORE checking IOP	
8	Evaluates IOP bilaterally using tonometry device** only AFTER ruling out OGI. For credit, *must avoid placing pressure on the lids while checking IOP*	
9	Checks the tightness of the lower eyelid against the globe for distractibility	
10	If mental status is intact, explain the procedure briefly and provide rationale for emergent treatment with verbal consent (*learner may defer this step and be given 1 point if patient is too altered to consent*)	
Performs LCC (the following steps must be performed in ***sequential order***)		
11	Administers sedative/pain medication if patient requests it or is anxious about the procedure (*learner may defer this step and be given 1 point if patient is too altered to require analgesics*)	
12	Anesthetizes the lateral canthus with lidocaine with epinephrine to anesthetize and reduce bleeding	
13	Clamps the lateral canthus to de-vascularize/reduce bleeding	
14	Disinfects the lateral canthal skin with betadine	
15	Cuts the lateral canthal angle to the orbital rim (canthotomy). Canthotomy successfully performed without significant adjacent soft tissue disruption	
16	Strums for inferior crus of lateral canthal tendon, then cuts inferior crus of lateral canthal tendon (cantholysis)	
17	Reassesses visual acuity and IOP	
18	If unchanged, cuts superior crus of lateral canthal tendon, then reassesses visual acuity and IOP	
19	Consults ophthalmology (*learner may perform at any time after step 9*)	

APD = afferent pupillary defect, IOP = intraocular pressure, LCC = lateral canthotomy and cantholysis, OGI = open globe injury

* If patient wears corrective lenses and does not have them in the ED, learner should test visual acuity using pinhole corrector.

** May use any tonometry device

### Appendix D MCQ Test and Answers for Assessment Before and/or After Participation in the OCS Simulation Case

[Table t10-jetem-9-4-s1]

**Table t10-jetem-9-4-s1:**

Item #	Question
1	A 26-year-old male with severe facial trauma has just undergone the indicated emergent treatment for clinical OCS of his right eye in the ED. On re-evaluation, he is only able to perceive hand motion OD (unchanged from prior to the procedure) and 20/40 OS, uncorrected. IOP is 50 mmHg OD, 19 mmHg OS. What is the next best step in management? Call ophthalmology Cut the superior crus Obtain orbital CT Wait a few minutes to see whether vision and IOP improve
2	You have diagnosed a 50-year-old female who was hit in the eye with a golf ball with OCS. You have quickly prepped and draped the patient. What is the next appropriate step of the procedure? Cut the lateral canthus Anesthetize the lateral canthus with lidocaine with epinephrine Anesthetize the lateral canthus with lidocaine without epinephrine Cut the inferior crus of the lateral canthal tendon
3	You are treating a 45-year-old male who was hit in the left eye with a baseball bat for OCS. His visual acuity was 20/30 OD, no light perception OS, corrected with glasses. You have just completed cutting the lateral canthal tendon. What step is indicated next? Call ophthalmology Obtain orbital CT Reassess visual acuity and IOP Fundoscopic exam
4	A 13-year-old male presents to the ED for severe left eye pain and vision loss after his brother shot him in the eye with a BB gun. Visual acuity is 20/20 OD, hand motion only OS, uncorrected. Pupils are equally round but slowly reactive with relative afferent pupillary defect (RAPD) OS. Visual fields are intact OD, extraocular movements intact (EOMI) in both eyes (OU). What must you do before assessing IOP? Perform a slit lamp exam Perform orbital CT Consult ophthalmology Evaluate for positive Seidel test
5	You are treating the 13-year-old male from question 4. Seidel test is negative. IOP is 15 OD, 40 OS. You have prepped and anesthetized the lateral canthus OS. What is the next appropriate step of the procedure? Clamp the lateral canthus Cut the lateral canthus Cut the inferior crus of the lateral canthal tendon Cut the lateral crus of the lateral canthal tendon
6	A 29-year-old female presents to the ED after a rock fell and hit her right eye while she was caving. She reports right eye pain and vision loss. On exam, visual acuity is hand motion only OD, 20/30 OS, uncorrected. Pupils are equally round but slowly reactive OD. Visual fields are intact OS, EOMI OU. Seidel test is negative. IOP is 60 OD, 25 OS. What is missing from this patient’s ocular examination? Fundoscopic exam Slit lamp examination Performing the swinging flashlight test CT orbit
7	An 18-year-old presents to the ED with left eye pain after he was elbowed in the eye in a mosh pit. Visual acuity is 20/20 uncorrected OD, but the patient is unable to see the Snellen chart with his left eye. Which type of visual acuity should you move on to assessing? Hand motion Light perception Confrontation Fingers
8	An 80-year-old female tripped over an electrical cord in her living room and hit her left eye on the corner of her coffee table. She complains of severe left eye pain and inability to see. You have tried to test her vision with a Snellen chart and by asking her to count fingers, but these methods have been unsuccessful. Which type of visual acuity should you move on to assessing? Light perception Hand motion Snellen Confrontation
9	A 65-year-old male with A. fib on Xarelto arrives in the trauma bay with right eye pain and vision loss after he crashes into a tree while skiing. He did not lose consciousness and GCS is 15. The patient cannot see the Snellen chart, fingers, or hand motions. Which type of visual acuity should you move on to assessing? Light perception Confrontation Fingers Hand motion
10	You receive a trauma transfer request from a small outside hospital. They are sending you a 12-year-old female who was kicked in the left eye by a horse. She received a head CT at the OSH which demonstrated no intracerebral hemorrhage (ICH). While in the ED, she has complained of progressively worsening left eye pain and trouble seeing. You are concerned that this patient may have orbital compartment syndrome based on the verbal report you have received. Which of the following is a classic physical examination finding for orbital compartment syndrome that you might expect in this patient? Photophobia Tightness of the lower lid against the globe Bulging sclera Enophthalmos

A. fib = atrial fibrillation, CT = computed tomography, ED = emergency department, IOP = intraocular pressure, OCS = orbital compartment syndrome, OD = right, OS = left, OSH = outside hospital, OU = bilateral

#### Answer Key

[Table t11-jetem-9-4-s1]

**Table t11-jetem-9-4-s1:**

Item #	Question	Answer
1	A 26-year-old male with severe facial trauma has just undergone the indicated emergent treatment for clinical OCS of his right eye in the ED. On re-evaluation he is only able to perceive hand motion OD (unchanged from prior to the procedure) and 20/40 OS, uncorrected. IOP is 50 mmHg OD, 19 mmHg OS. What is the next best step in management? Call ophthalmology Cut the superior crus Obtain orbital CT Wait a few minutes to see whether vision and IOP improve	B
2	You have diagnosed a 50-year-old female who was hit in the eye with a golf ball with OCS. You have quickly prepped and draped the patient. What is the next appropriate step of the procedure? Cut the lateral canthus Anesthetize the lateral canthus with lidocaine with epinephrine Anesthetize the lateral canthus with lidocaine without epinephrine Cut the inferior crus of the lateral canthal tendon	B
3	You are treating a 45-year-old male who was hit in the left eye with a baseball bat for OCS. His visual acuity was 20/30 OD, no light perception OS, corrected with glasses. You have just completed cutting the lateral canthal tendon. What step is indicated next? Call ophthalmology Obtain orbital CT Reassess visual acuity and IOP Fundoscopic exam	C
4	A 13-year-old male presents to the ED for severe left eye pain and vision loss after his brother shot him in the eye with a BB gun. Visual acuity is 20/20 OD, hand motion only OS, uncorrected. Pupils are equally round but slowly reactive with RAPD OS. Visual fields are intact OD, EOMI OU. What must you do before assessing IOP? Perform a slit lamp exam Perform orbital CT Consult ophthalmology Evaluate for positive Seidel test	D
5	You are treating the 13-year-old male from question 4. Seidel test is negative. IOP is 15 OD, 40 OS. You have prepped and anesthetized the lateral canthus OS. What is the next appropriate step of the procedure? Clamp the lateral canthus Cut the lateral canthus Cut the inferior crus of the lateral canthal tendon Cut the lateral crus of the lateral canthal tendon	A
6	A 29-year-old female presents to the ED after a rock fell and hit her right eye while she was caving. She reports right eye pain and vision loss. On exam, visual acuity is hand motion only OD, 20/30 OS, uncorrected. Pupils are equally round but slowly reactive OD. Visual fields are intact OS, EOMI OU. Seidel test is negative. IOP is 60 OD, 25 OS. What is missing from this patient’s ocular examination? Fundoscopic exam Slit lamp examination Performing the swinging flashlight test CT orbit	C
7	An 18-year-old presents to the ED with left eye pain after he was elbowed in the eye in a mosh pit. Visual acuity is 20/20 uncorrected OD but the patient is unable to see the Snellen chart with his left eye. Which type of visual acuity should you move on to assessing? Hand motion Light perception Confrontation Fingers	C or D (these are technically the same)
8	An 80-year-old female tripped over an electrical cord in her living room and hit her left eye on the corner of her coffee table. She complains of severe left eye pain and inability to see. You have tried to test her vision with a Snellen chart and by asking her to count fingers, but these methods have been unsuccessful. Which type of visual acuity should you move on to assessing? Light perception Hand motion Snellen Confrontation	B
9	A 65-year-old male with A. fib on Xarelto arrives in the trauma bay with right eye pain and vision loss after he crashes into a tree while skiing. He did not lose consciousness and GCS is 15. The patient cannot see the Snellen chart, fingers, or hand motions. Which type of visual acuity should you move on to assessing? Light perception Confrontation Fingers Hand motion	A
10	You receive a trauma transfer request from a small outside hospital. They are sending you a 12-year-old female who was kicked in the left eye by a horse. She received a head CT at the OSH which demonstrated no ICH. While in the ED she has complained of progressively worsening left eye pain and trouble seeing. You are concerned that this patient may have orbital compartment syndrome based on the verbal report you have received. Which of the following is a classic physical examination finding for orbital compartment syndrome that you might expect in this patient? Photophobia Tightness of the lower lid against the globe Bulging sclera Enophthalmos	B

A. fib = atrial fibrillation, CT = computed tomography, ED = emergency department, IOP = intraocular pressure, OCS = orbital compartment syndrome, OD = right, OS = left, OSH = outside hospital, OU = bilateral

### Appendix E Self-Efficacy Scale (SES)

[Table t12-jetem-9-4-s1]

**Table t12-jetem-9-4-s1:**

Task	1 Very Uncomfortable	2	3	4	5 Very Comfortable
Treating a patient with an ophthalmologic complaint					
Approaching the ophthalmologic exam systematically					
Developing a differential diagnosis for sight-threatening diagnoses that could cause eye pain					
Developing a differential diagnosis for sight-threatening diagnoses that could cause vision loss					
Checking for an APD					
Assessing IOP					
Measuring visual acuity using a Snellen chart					
Identifying clinical OCS					
Performing a LCC					
*Performing a slit lamp exam*					
*Using POCUS as a diagnostic adjunct for patients with eye complaints*					
*Removing a foreign body from the eye*					
*Diagnosing an OGI*					
*Differentiating central from peripheral causes of vision loss*					

APD = afferent pupillary defect, IOP = intraocular pressure, LCC = lateral canthotomy and cantholysis, OCS = orbital compartment syndrome, OGI = open globe injury, POCUS = point-of-care ultrasound

### Appendix F Stimuli for Simulation Case

Please see associated PowerPoint file

## References

[1] Moxon NR, Goyal A, Giaconi JA (2020). The state of medical student education in the United States: An update. Ophthalmology.

[2] Uhr JH, Governatori NJ, Zhang Q (2020). Training in and comfort with diagnosis and management of ophthalmic emergencies among emergency medicine physicians in the United States. Eye.

[3] Phillips L, Stack L, Thurman RJ (2015). Addressing ophthalmology education for newly matriculated emergency medicine residents using innovative models. Simul Healthc J Soc Simul Healthc.

[4] Abbott EF, Laack TA, Licatino LK (2021). Comparison of dyad versus individual simulation-based training on stress, anxiety, cognitive load, and performance: A randomized controlled trial. BMC Med Educ.

[5] Vo V, Johnson W, Nordt S, Mattu A, Swadron S Lateral Canthotomy. CorePendium.

[6] Dupre A, Vojta L Red and painful eye. Rosen’s Emergency Medicine: Concepts and Clinical Practice.

[7] Perin A, Bayram J, Uwaydat S, Reichman EF (2019). Acute orbital compartment syndrome (retrobulbar hemorrhage) management. Reichman’s Emergency Medicine Procedures.

[8] Downing SM, Tekian A, Yudkowsky R (2006). Research methodology: Procedures for establishing defensible absolute passing scores on performance examinations in health professions education. Teach Learn Med.

[9] Witheridge A, Ferns G, Scott-Smith W (2019). Revisiting Miller’s pyramid in medical education: the gap between traditional assessment and diagnostic reasoning. Int J Med Educ.

[10] Rossi PH, Lipsey MW, Henry GT (2019). Evaluation: A Systematic Approach.

[11] Andreatta P, Saxton E, Thompson M, Annich G (2011). Simulation-based mock codes significantly correlate with improved pediatric patient cardiopulmonary arrest survival rates. Pediatr Crit Care Med.

[12] Murali S, Davis C, McCrea MJ (2021). Orbital compartment syndrome: Pearls and pitfalls for the emergency physician. J Am Coll Emerg Physicians Open.

[13] Pelletier J, Koyfman A, Long B (2022). High risk and low prevalence diseases: Open globe injury. Am J Emerg Med.

[14] McCallum E, Keren S, Lapira M, Norris JH (2019). Orbital compartment syndrome: An update with review of the literature. Clin Ophthalmol.

[15] Edmunds MR, Haridas AS, Morris DS, Jamalapuram K (2019). Management of acute retrobulbar haemorrhage: a survey of non-ophthalmic emergency department physicians. Emerg Med J.

[16] Adrian J, Moreira M (2022). Eye trauma. Corependium.

[17] Swaminathan A, Milne K Cognitive biases.

[18] Etchells E (2015). Anchoring bias with critical implications PSNet [internet].

[19] Chinai S, Guth T, Lovell E, Epter M Taking advantage of the teachable moment: A review of learner-centered clinical teaching models. West J Emerg Med.

