# Supplementary material for: A Case of Painful Visual Loss - Managing Orbital Compartment Syndrome in the Emergency Department
Source: J Educ Teach Emerg Med. 2024 Oct 31;9(4):S1–S50. doi: 10.21980/J8N35D (PMC11537727; doi:10.21980/J8N35D)
Supplement: Supplementary file 1 — Please see associated PowerPoint file [file 9-4-S1-Appendix_A.pptx]

## Slide 1
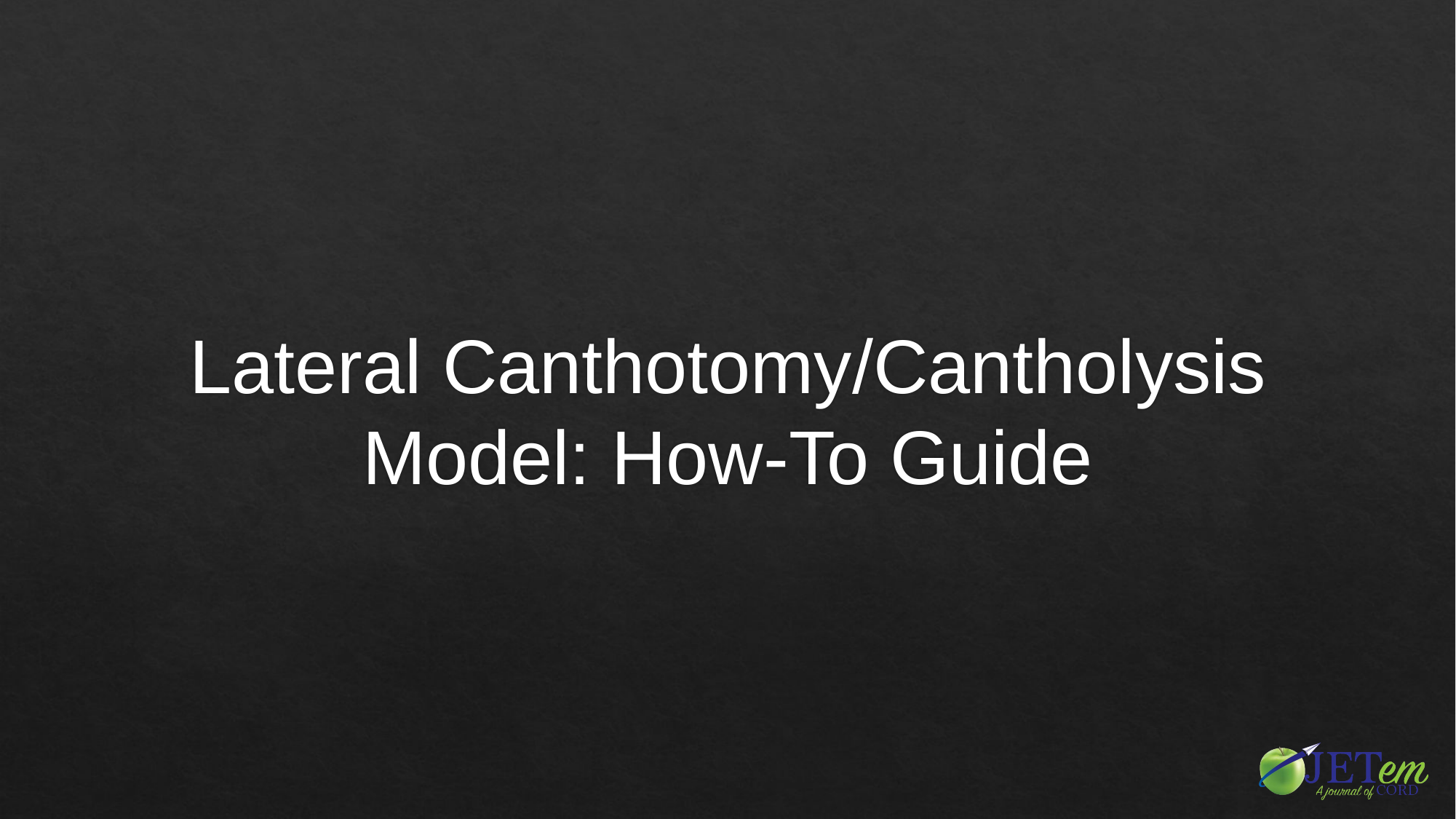

# Lateral Canthotomy/Cantholysis Model: How-To Guide

## Slide 2
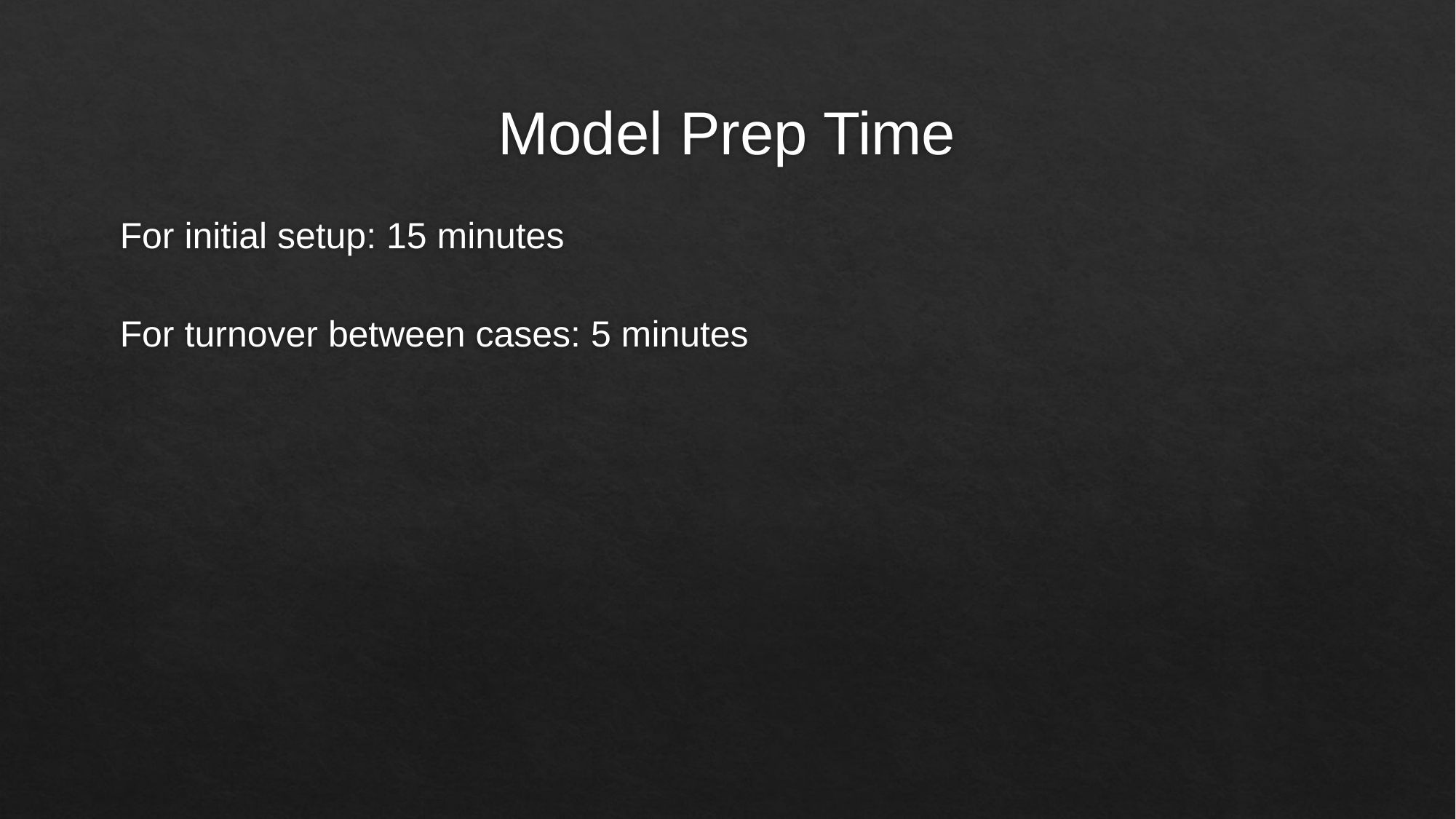

# Model Prep Time
For initial setup: 15 minutes
For turnover between cases: 5 minutes

## Slide 3
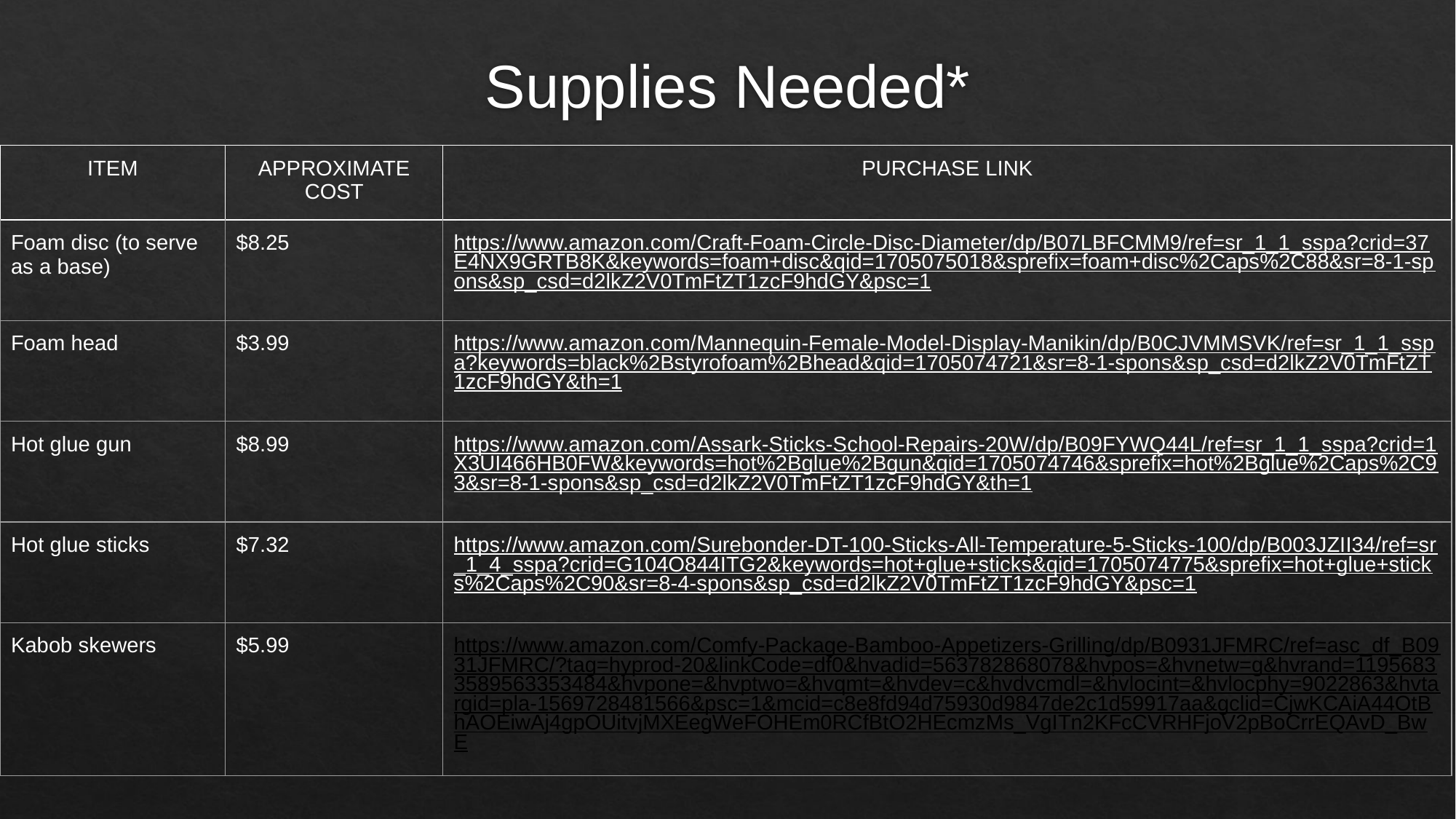

# Supplies Needed*
| ITEM | APPROXIMATE COST | PURCHASE LINK |
| --- | --- | --- |
| Foam disc (to serve as a base) | $8.25 | https://www.amazon.com/Craft-Foam-Circle-Disc-Diameter/dp/B07LBFCMM9/ref=sr\_1\_1\_sspa?crid=37E4NX9GRTB8K&keywords=foam+disc&qid=1705075018&sprefix=foam+disc%2Caps%2C88&sr=8-1-spons&sp\_csd=d2lkZ2V0TmFtZT1zcF9hdGY&psc=1 |
| Foam head | $3.99 | https://www.amazon.com/Mannequin-Female-Model-Display-Manikin/dp/B0CJVMMSVK/ref=sr\_1\_1\_sspa?keywords=black%2Bstyrofoam%2Bhead&qid=1705074721&sr=8-1-spons&sp\_csd=d2lkZ2V0TmFtZT1zcF9hdGY&th=1 |
| Hot glue gun | $8.99 | https://www.amazon.com/Assark-Sticks-School-Repairs-20W/dp/B09FYWQ44L/ref=sr\_1\_1\_sspa?crid=1X3UI466HB0FW&keywords=hot%2Bglue%2Bgun&qid=1705074746&sprefix=hot%2Bglue%2Caps%2C93&sr=8-1-spons&sp\_csd=d2lkZ2V0TmFtZT1zcF9hdGY&th=1 |
| Hot glue sticks | $7.32 | https://www.amazon.com/Surebonder-DT-100-Sticks-All-Temperature-5-Sticks-100/dp/B003JZII34/ref=sr\_1\_4\_sspa?crid=G104O844ITG2&keywords=hot+glue+sticks&qid=1705074775&sprefix=hot+glue+sticks%2Caps%2C90&sr=8-4-spons&sp\_csd=d2lkZ2V0TmFtZT1zcF9hdGY&psc=1 |
| Kabob skewers | $5.99 | https://www.amazon.com/Comfy-Package-Bamboo-Appetizers-Grilling/dp/B0931JFMRC/ref=asc\_df\_B0931JFMRC/?tag=hyprod-20&linkCode=df0&hvadid=563782868078&hvpos=&hvnetw=g&hvrand=11956833589563353484&hvpone=&hvptwo=&hvqmt=&hvdev=c&hvdvcmdl=&hvlocint=&hvlocphy=9022863&hvtargid=pla-1569728481566&psc=1&mcid=c8e8fd94d75930d9847de2c1d59917aa&gclid=CjwKCAiA44OtBhAOEiwAj4gpOUitvjMXEegWeFOHEm0RCfBtO2HEcmzMs\_VgITn2KFcCVRHFjoV2pBoCrrEQAvD\_BwE |

## Slide 4
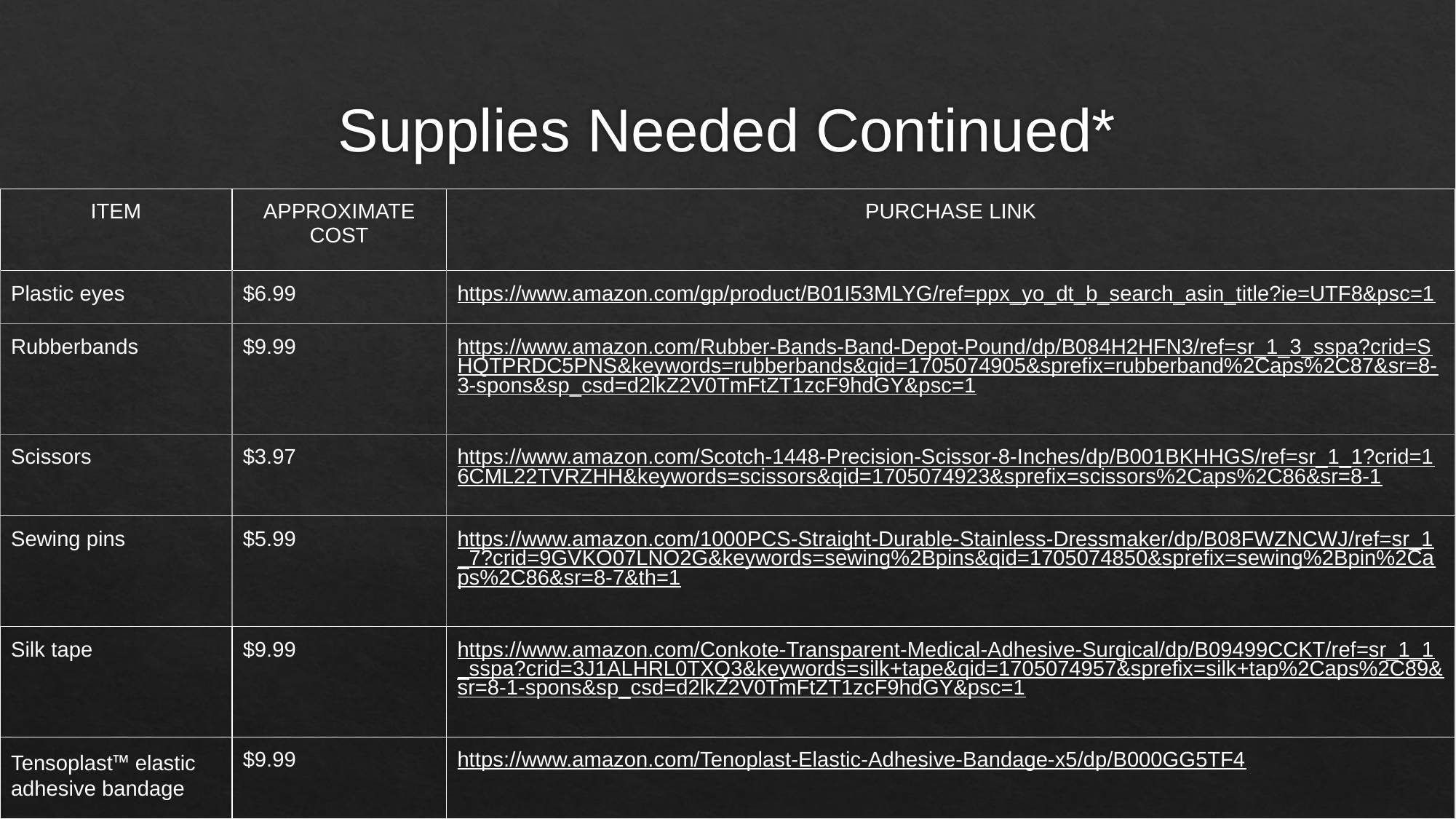

# Supplies Needed Continued*
| ITEM | APPROXIMATE COST | PURCHASE LINK |
| --- | --- | --- |
| Plastic eyes | $6.99 | https://www.amazon.com/gp/product/B01I53MLYG/ref=ppx\_yo\_dt\_b\_search\_asin\_title?ie=UTF8&psc=1 |
| Rubberbands | $9.99 | https://www.amazon.com/Rubber-Bands-Band-Depot-Pound/dp/B084H2HFN3/ref=sr\_1\_3\_sspa?crid=SHQTPRDC5PNS&keywords=rubberbands&qid=1705074905&sprefix=rubberband%2Caps%2C87&sr=8-3-spons&sp\_csd=d2lkZ2V0TmFtZT1zcF9hdGY&psc=1 |
| Scissors | $3.97 | https://www.amazon.com/Scotch-1448-Precision-Scissor-8-Inches/dp/B001BKHHGS/ref=sr\_1\_1?crid=16CML22TVRZHH&keywords=scissors&qid=1705074923&sprefix=scissors%2Caps%2C86&sr=8-1 |
| Sewing pins | $5.99 | https://www.amazon.com/1000PCS-Straight-Durable-Stainless-Dressmaker/dp/B08FWZNCWJ/ref=sr\_1\_7?crid=9GVKO07LNO2G&keywords=sewing%2Bpins&qid=1705074850&sprefix=sewing%2Bpin%2Caps%2C86&sr=8-7&th=1 |
| Silk tape | $9.99 | https://www.amazon.com/Conkote-Transparent-Medical-Adhesive-Surgical/dp/B09499CCKT/ref=sr\_1\_1\_sspa?crid=3J1ALHRL0TXQ3&keywords=silk+tape&qid=1705074957&sprefix=silk+tap%2Caps%2C89&sr=8-1-spons&sp\_csd=d2lkZ2V0TmFtZT1zcF9hdGY&psc=1 |
| Tensoplast™ elastic adhesive bandage | $9.99 | https://www.amazon.com/Tenoplast-Elastic-Adhesive-Bandage-x5/dp/B000GG5TF4 |

## Slide 5
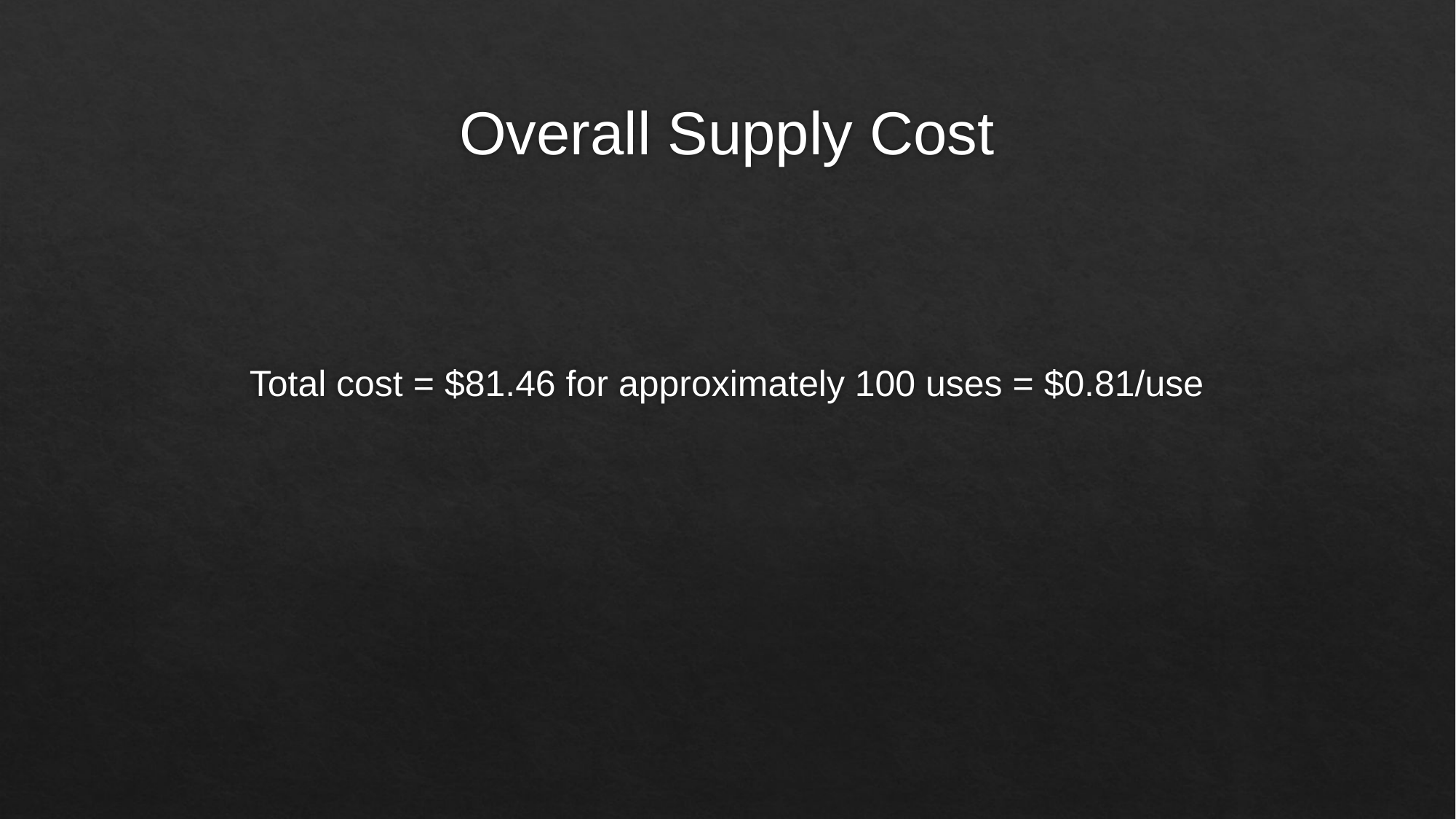

# Overall Supply Cost
Total cost = $81.46 for approximately 100 uses = $0.81/use

## Slide 6
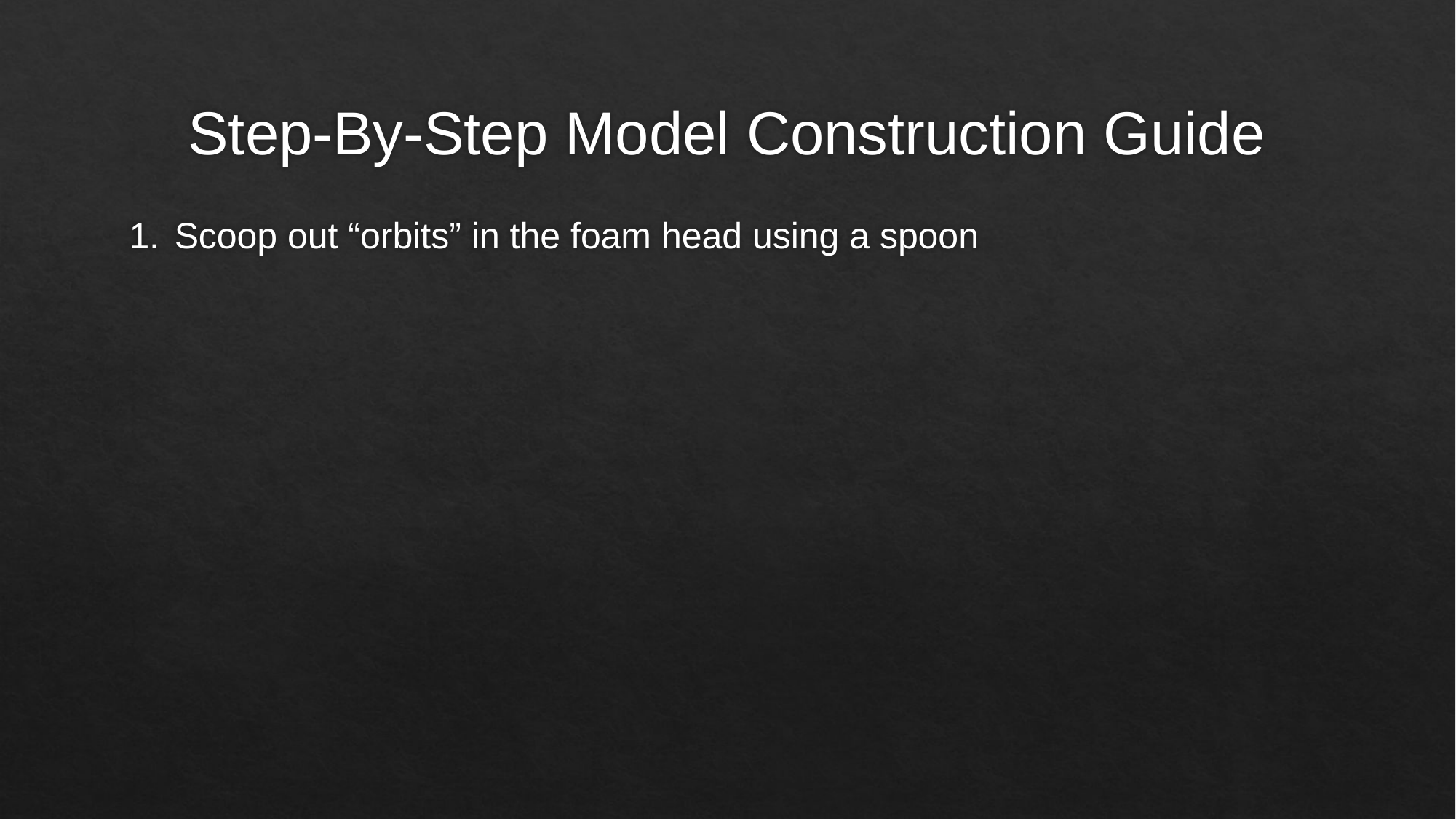

# Step-By-Step Model Construction Guide
Scoop out “orbits” in the foam head using a spoon

## Slide 7
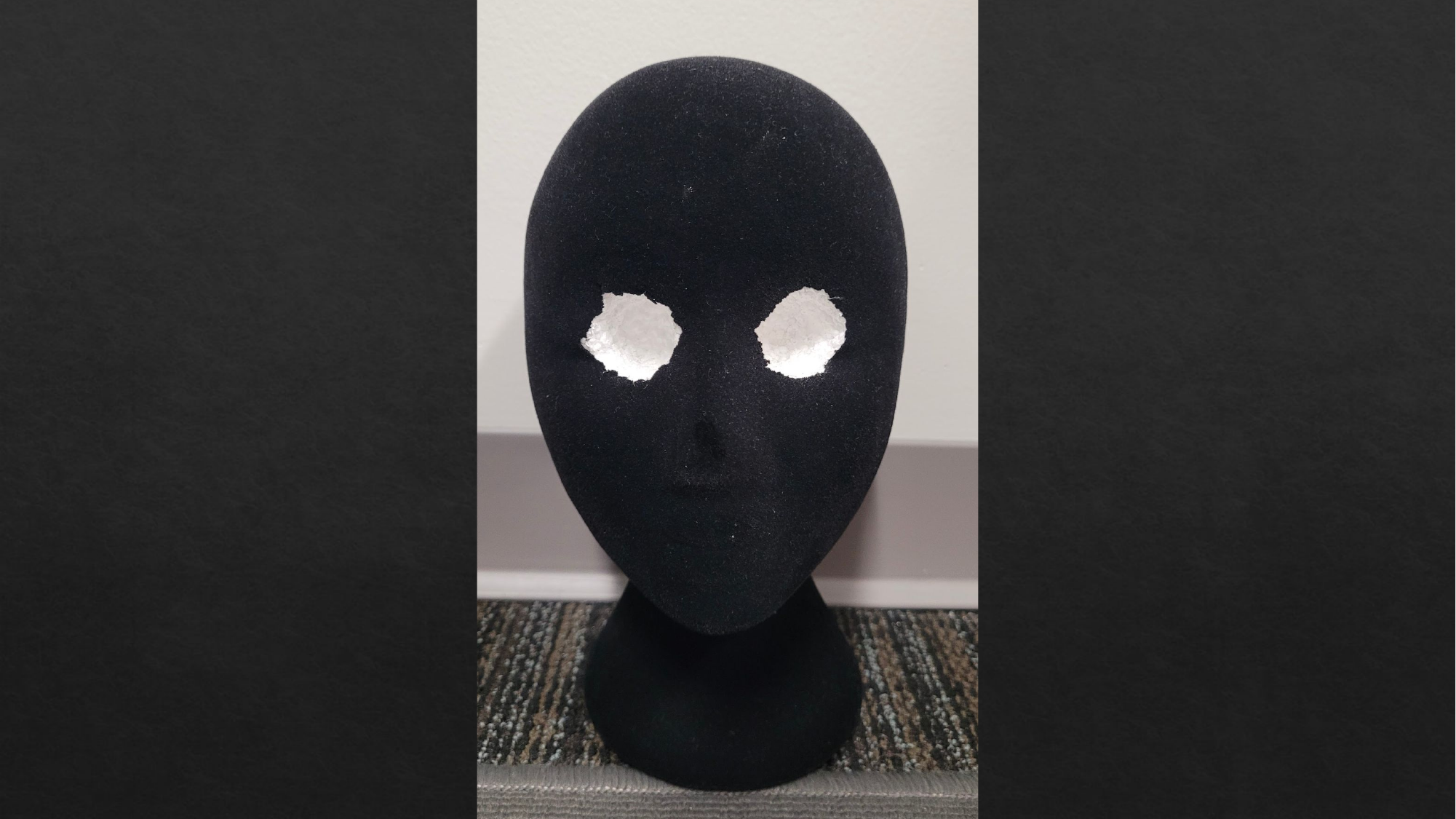

## Slide 8
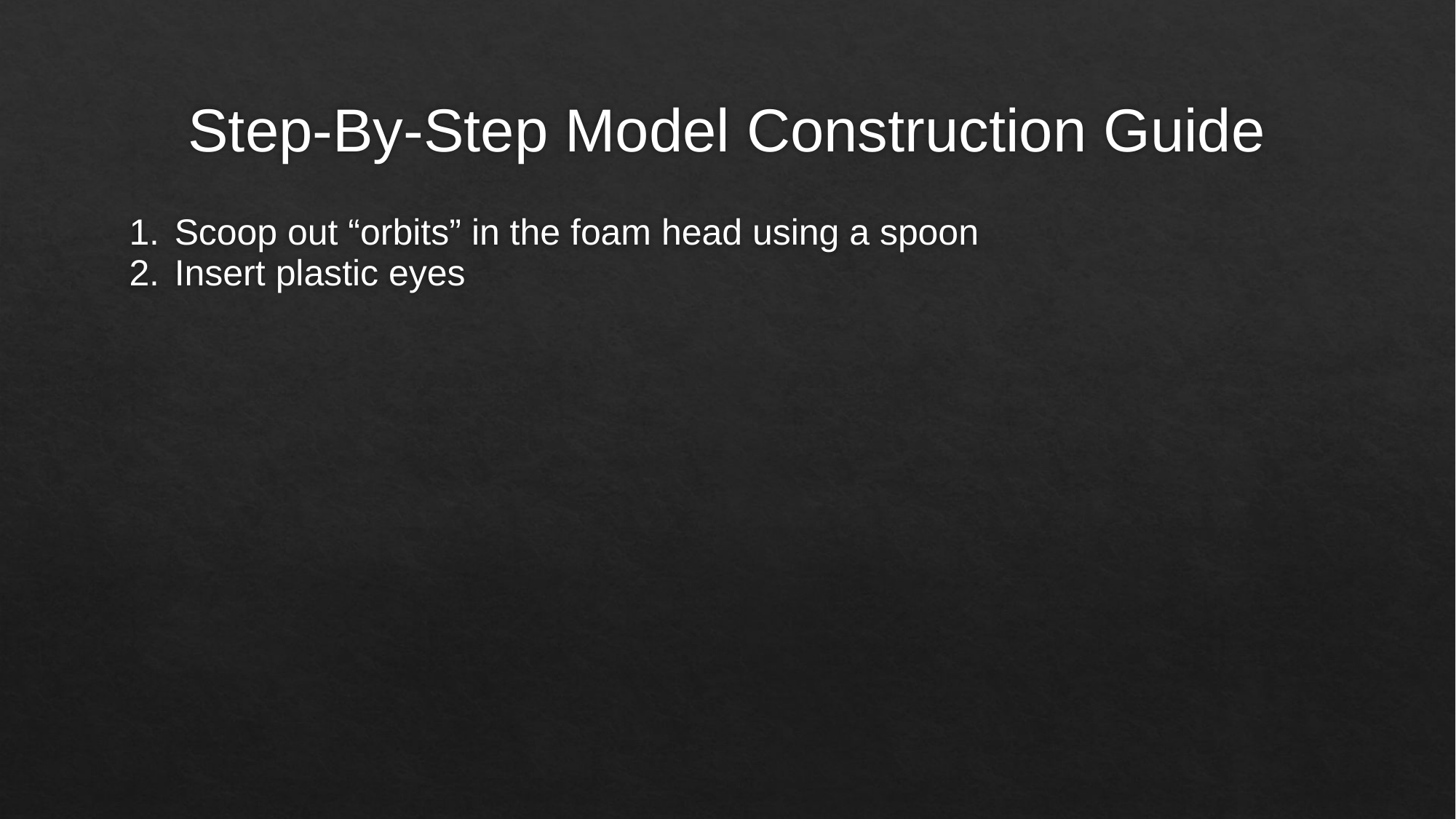

# Step-By-Step Model Construction Guide
Scoop out “orbits” in the foam head using a spoon
Insert plastic eyes

## Slide 9
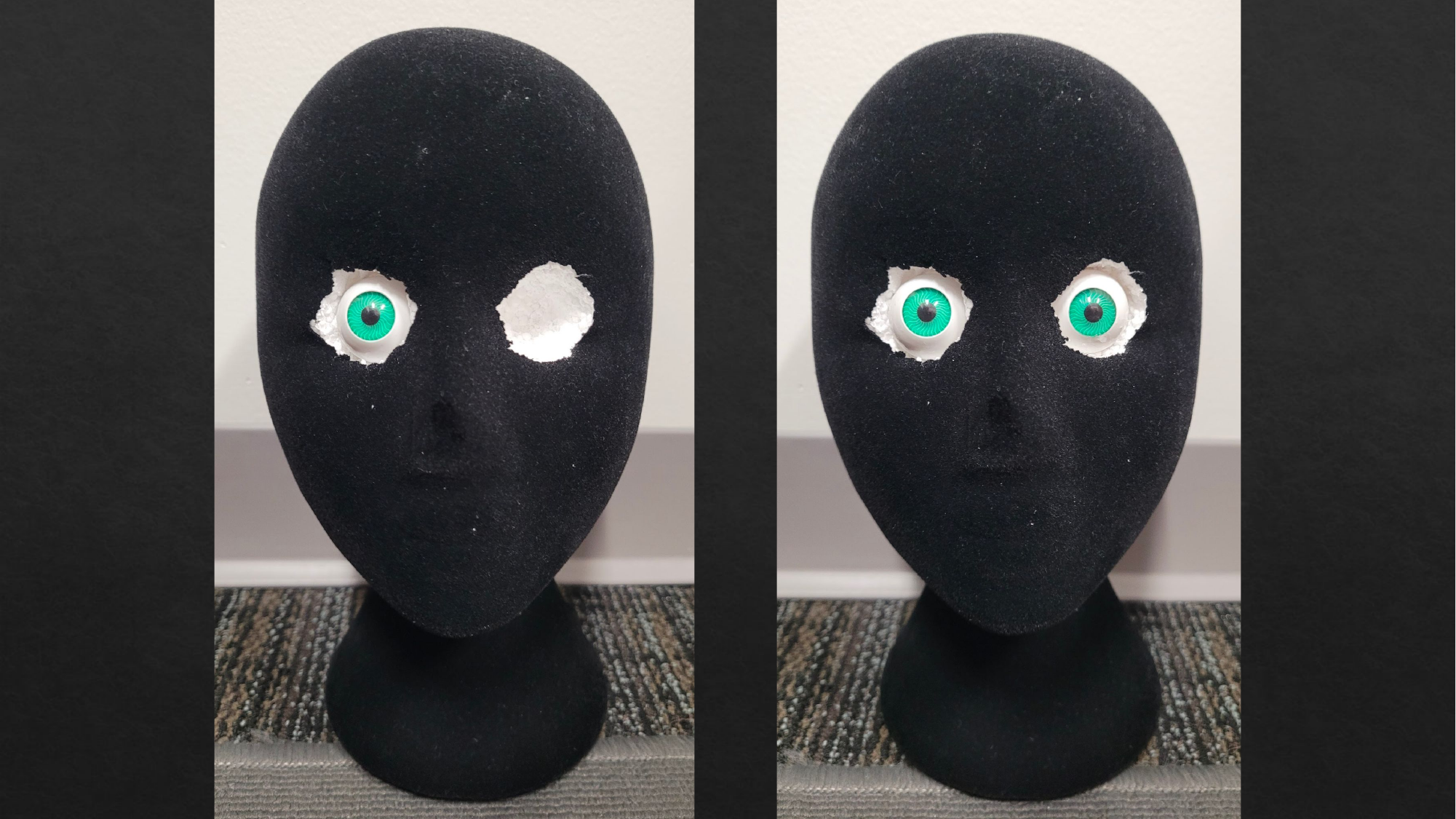

## Slide 10
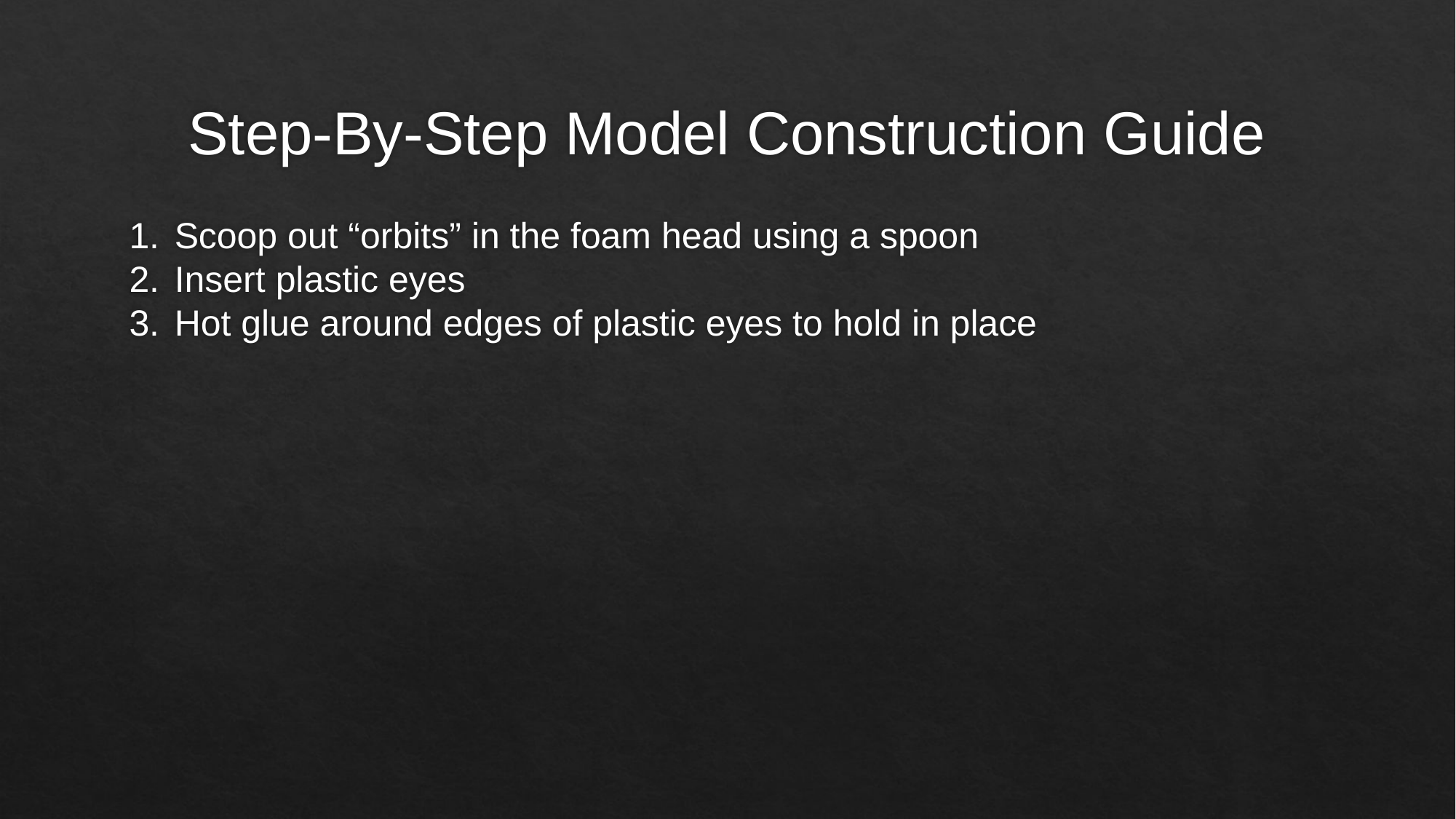

# Step-By-Step Model Construction Guide
Scoop out “orbits” in the foam head using a spoon
Insert plastic eyes
Hot glue around edges of plastic eyes to hold in place

## Slide 11
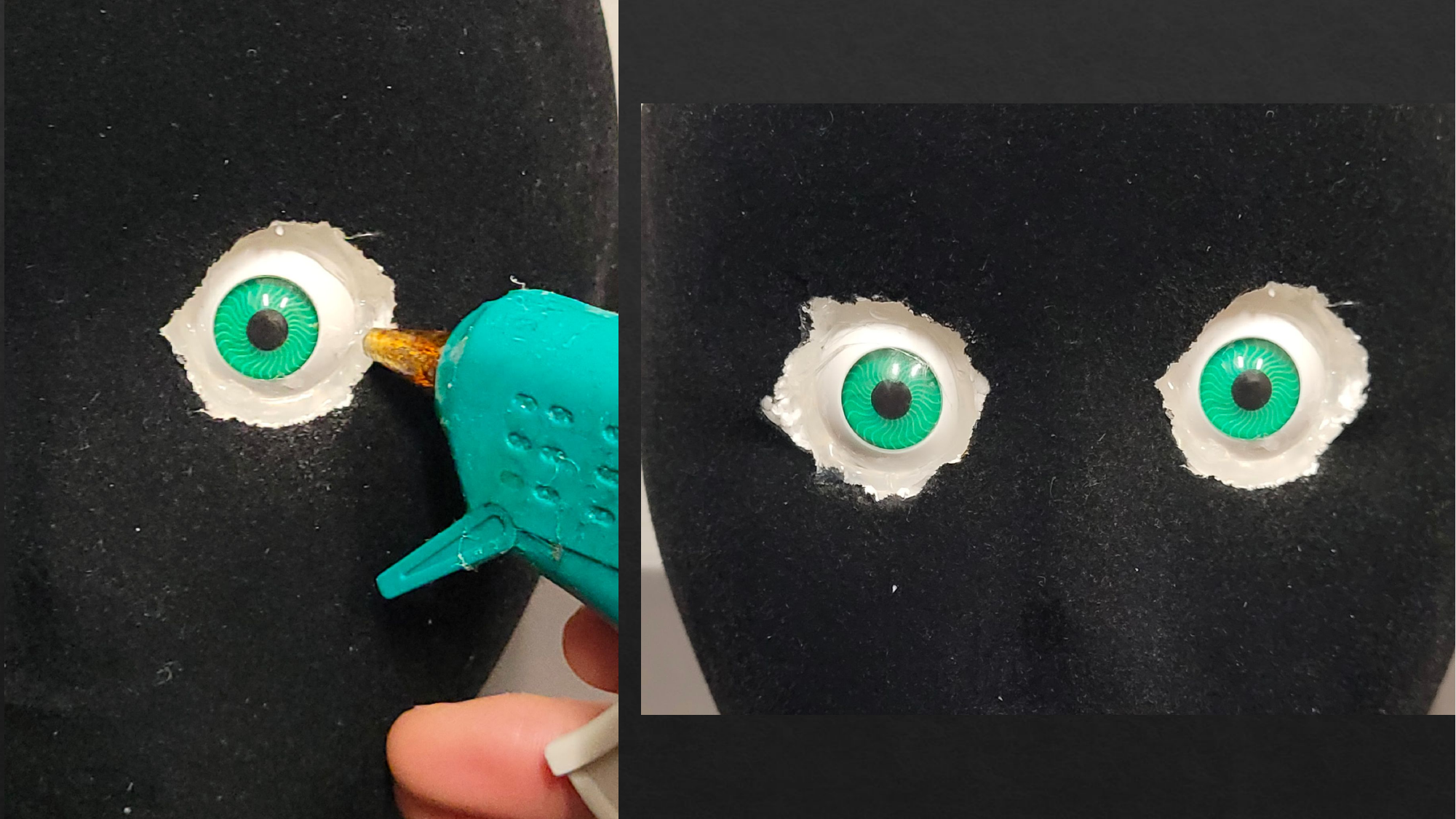

## Slide 12
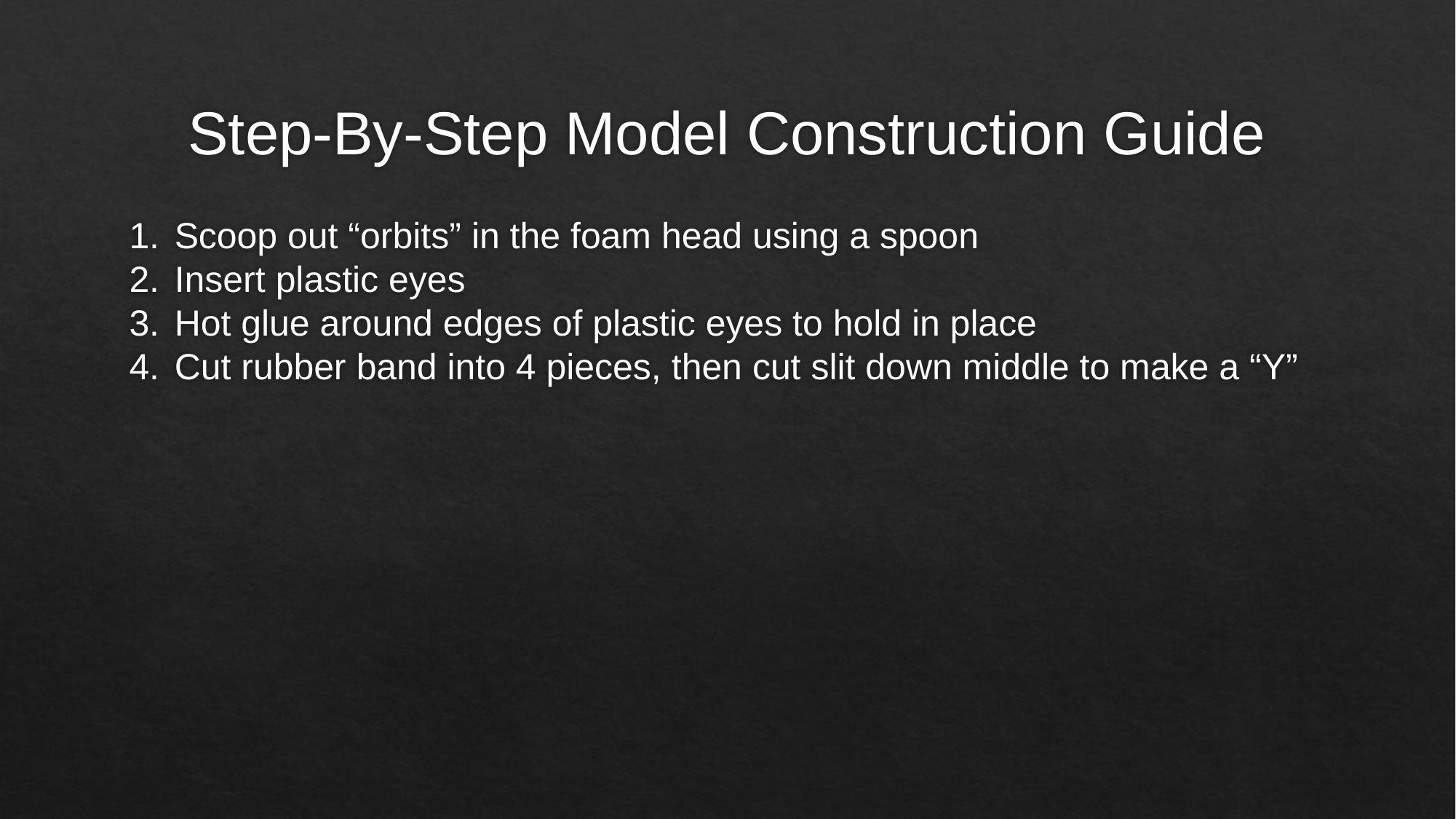

# Step-By-Step Model Construction Guide
Scoop out “orbits” in the foam head using a spoon
Insert plastic eyes
Hot glue around edges of plastic eyes to hold in place
Cut rubber band into 4 pieces, then cut slit down middle to make a “Y”

## Slide 13
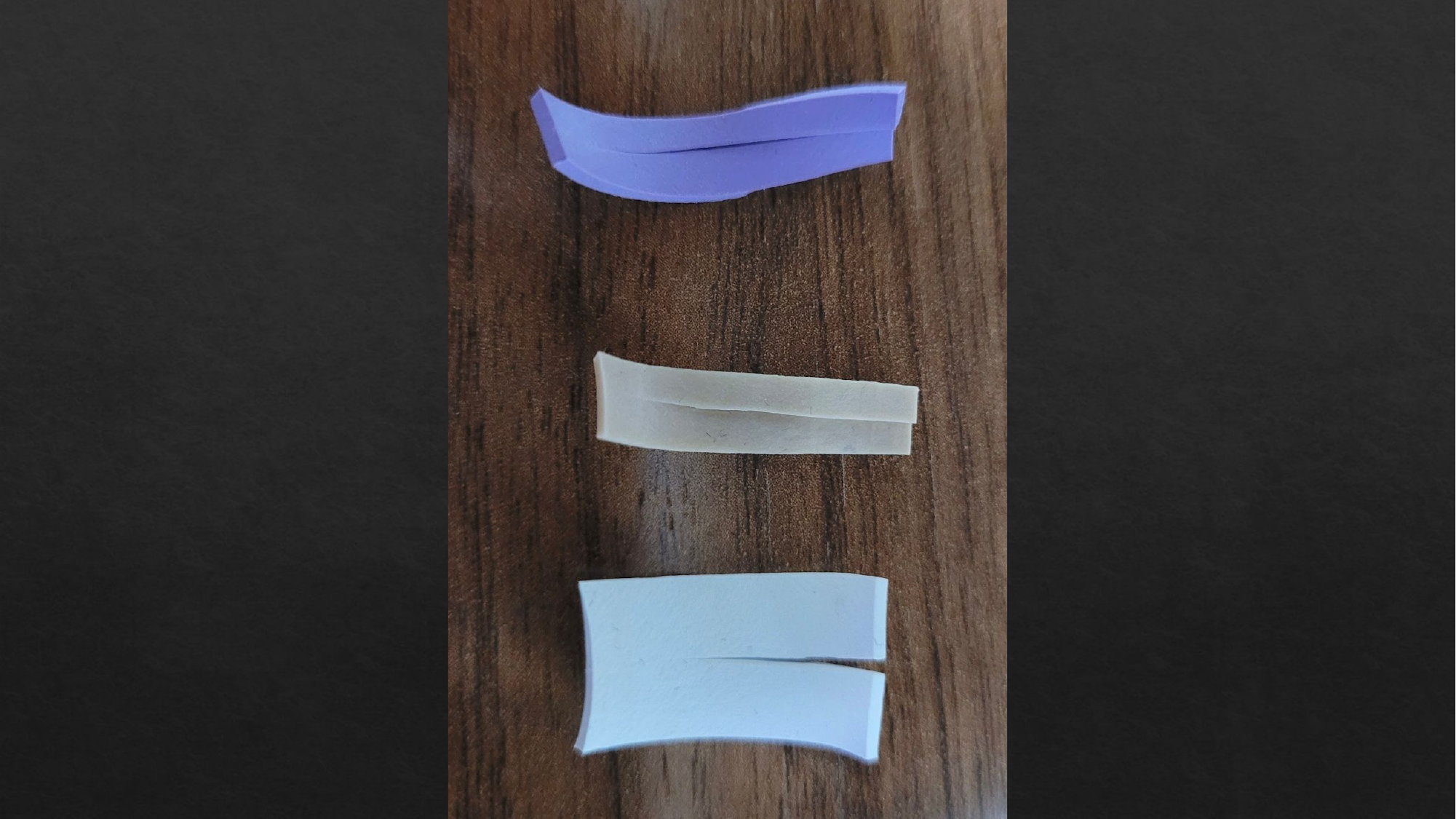

## Slide 14
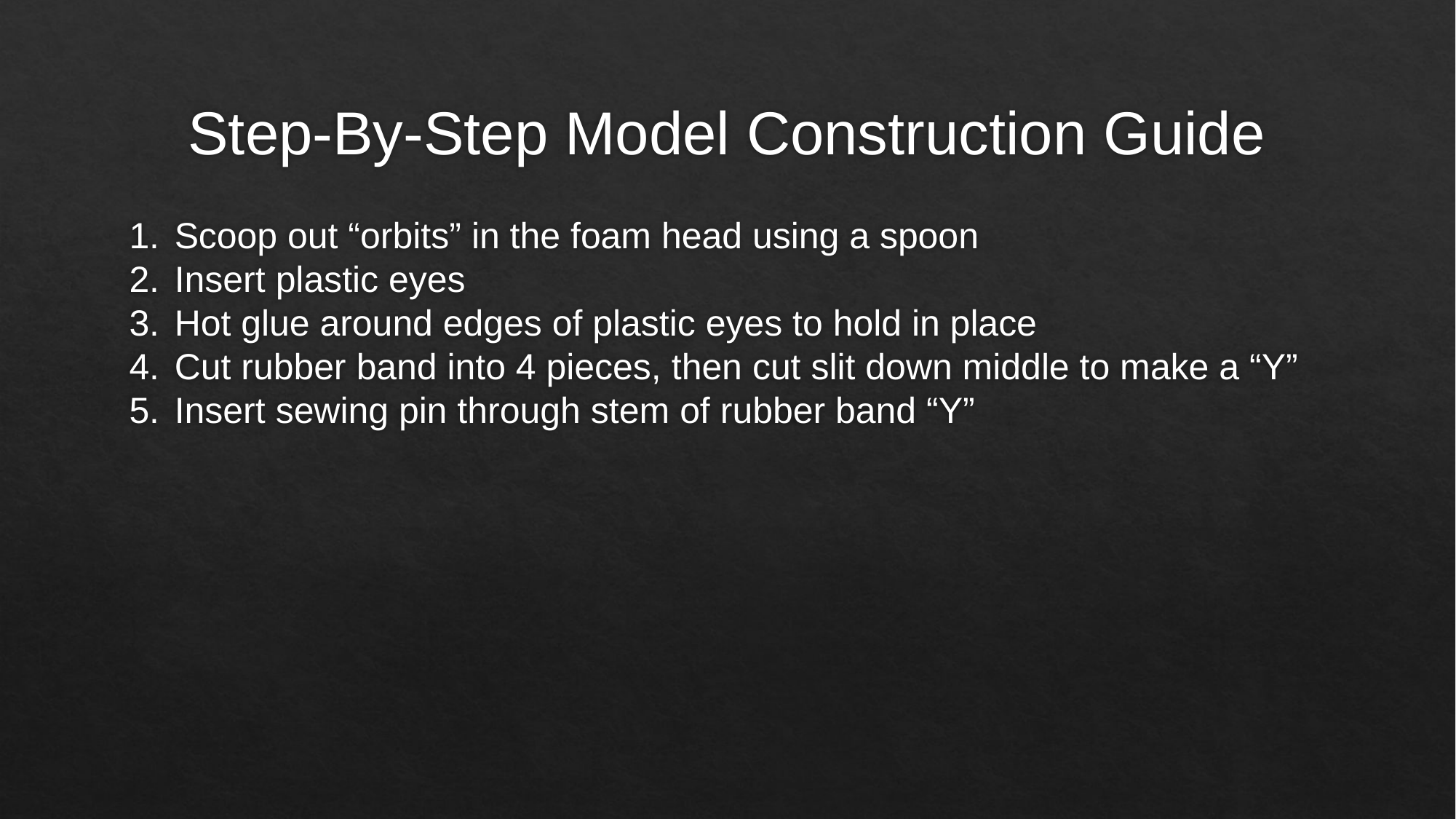

# Step-By-Step Model Construction Guide
Scoop out “orbits” in the foam head using a spoon
Insert plastic eyes
Hot glue around edges of plastic eyes to hold in place
Cut rubber band into 4 pieces, then cut slit down middle to make a “Y”
Insert sewing pin through stem of rubber band “Y”

## Slide 15
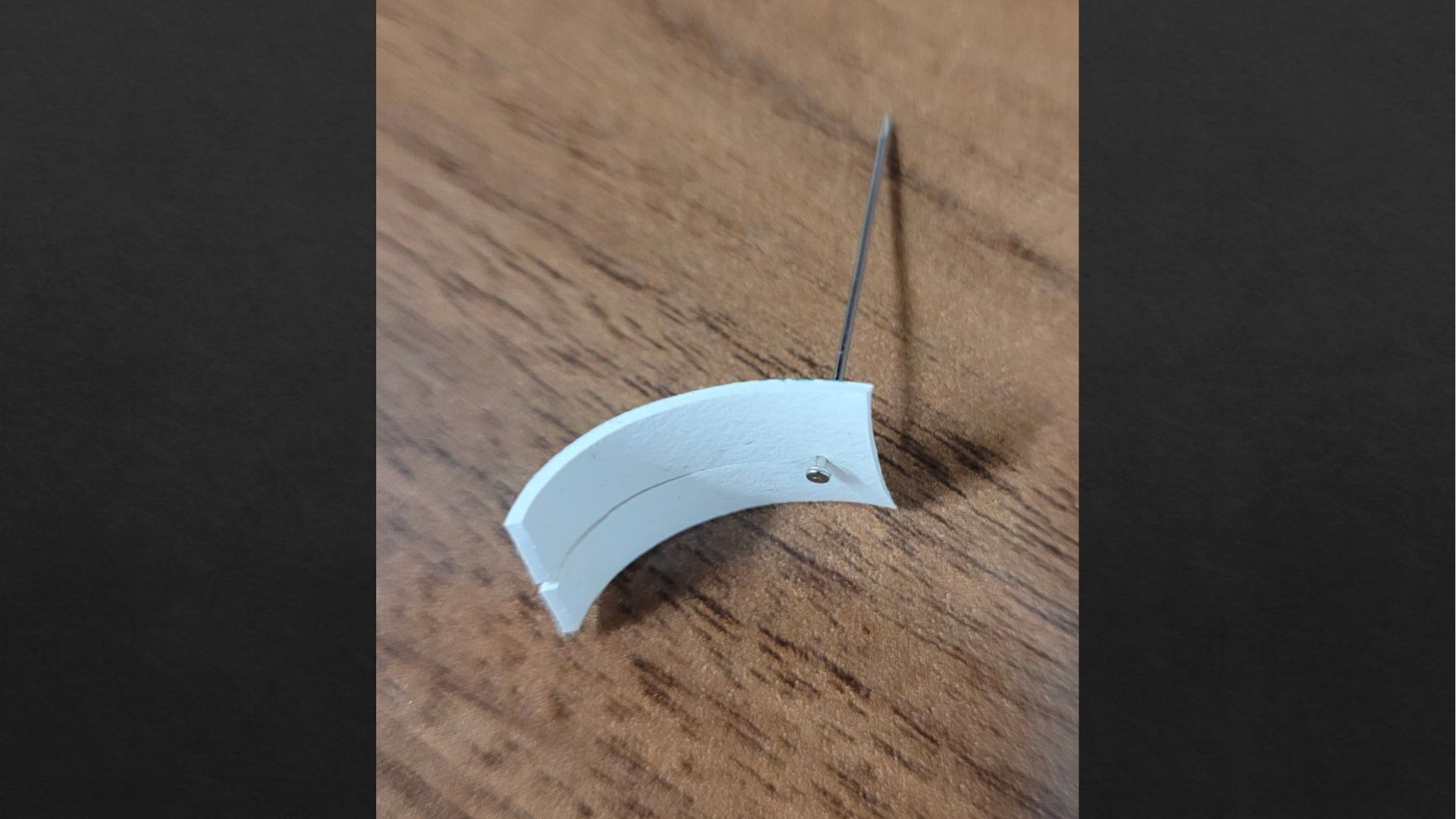

## Slide 16
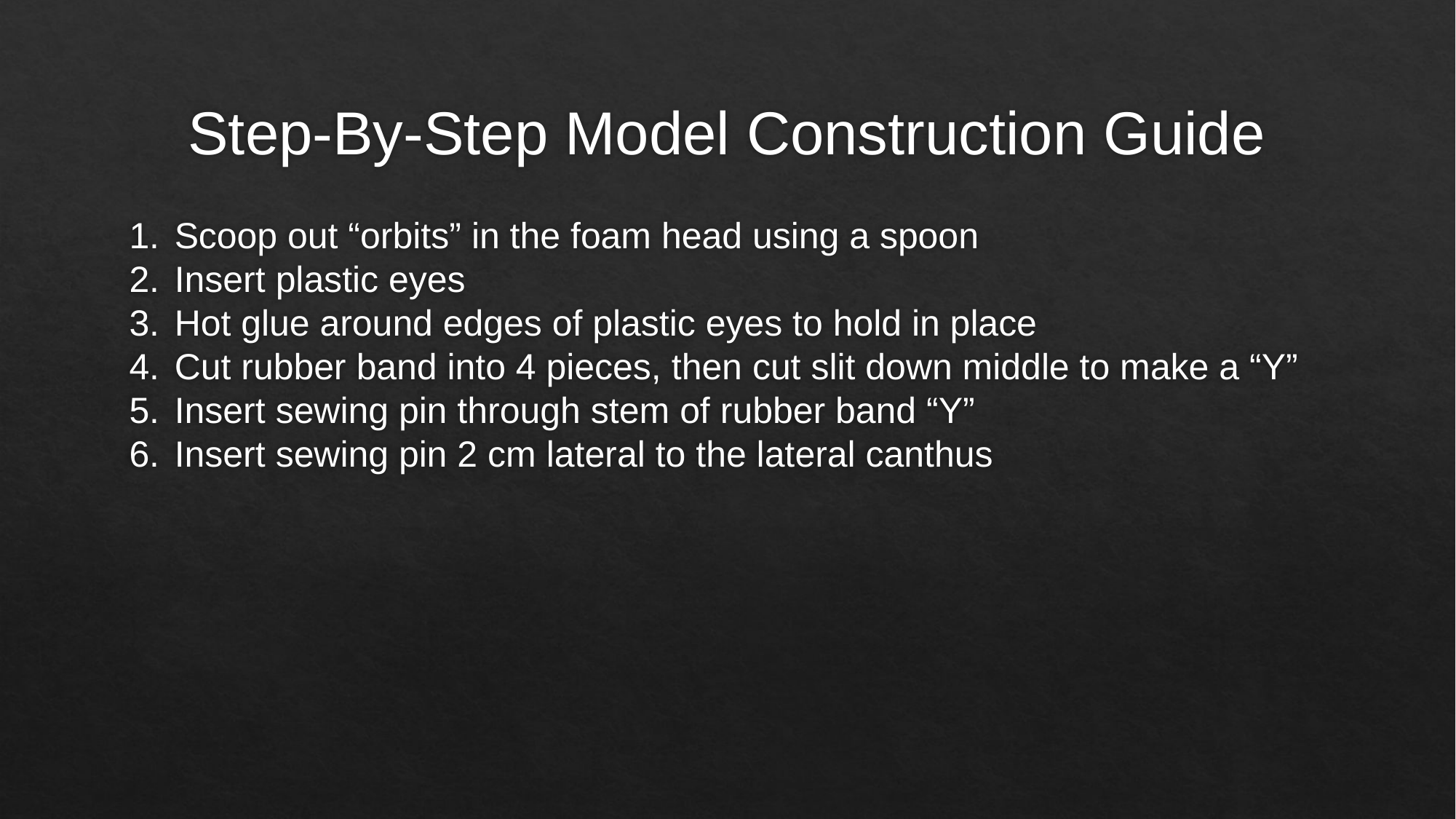

# Step-By-Step Model Construction Guide
Scoop out “orbits” in the foam head using a spoon
Insert plastic eyes
Hot glue around edges of plastic eyes to hold in place
Cut rubber band into 4 pieces, then cut slit down middle to make a “Y”
Insert sewing pin through stem of rubber band “Y”
Insert sewing pin 2 cm lateral to the lateral canthus

## Slide 17
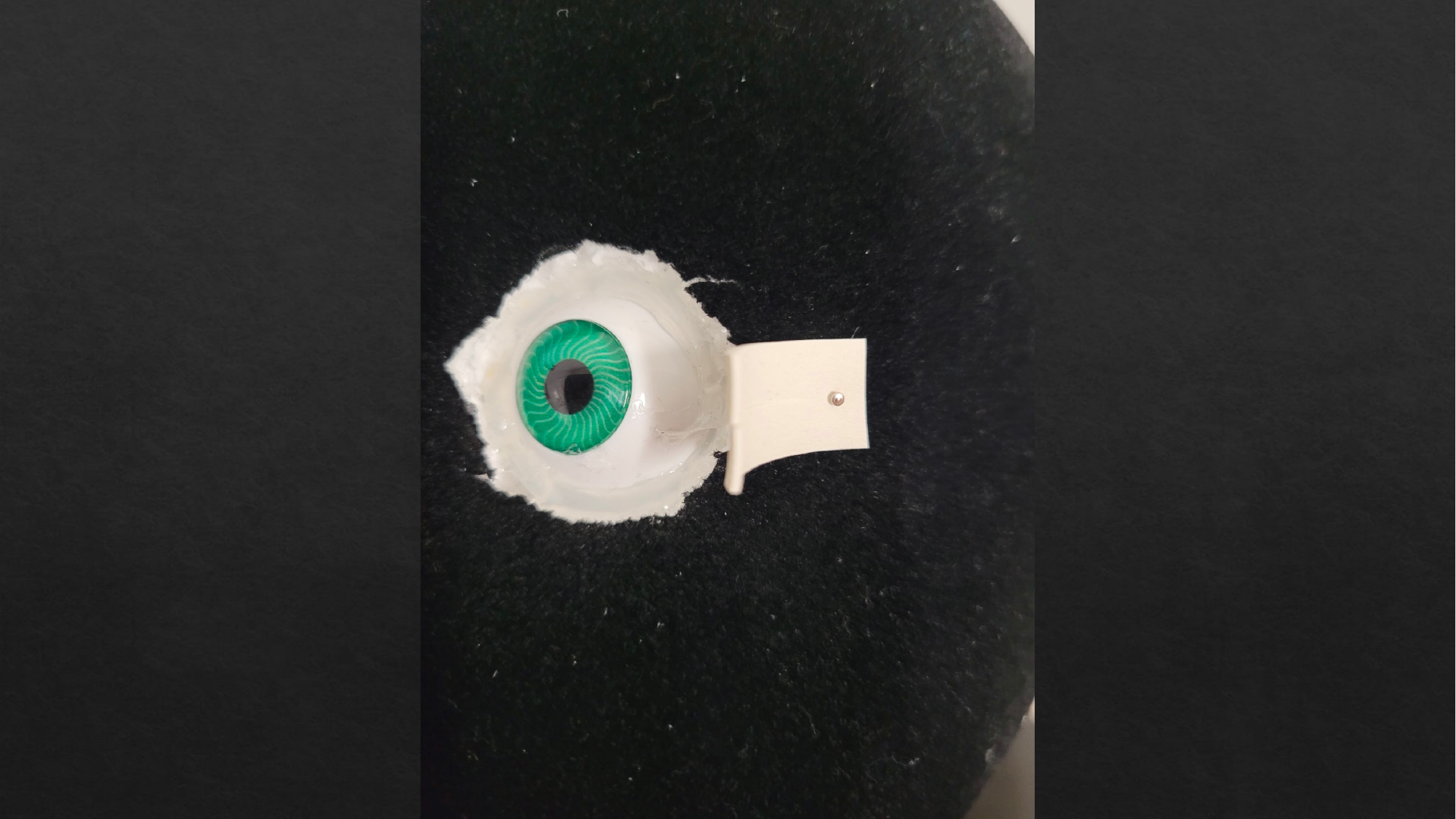

## Slide 18
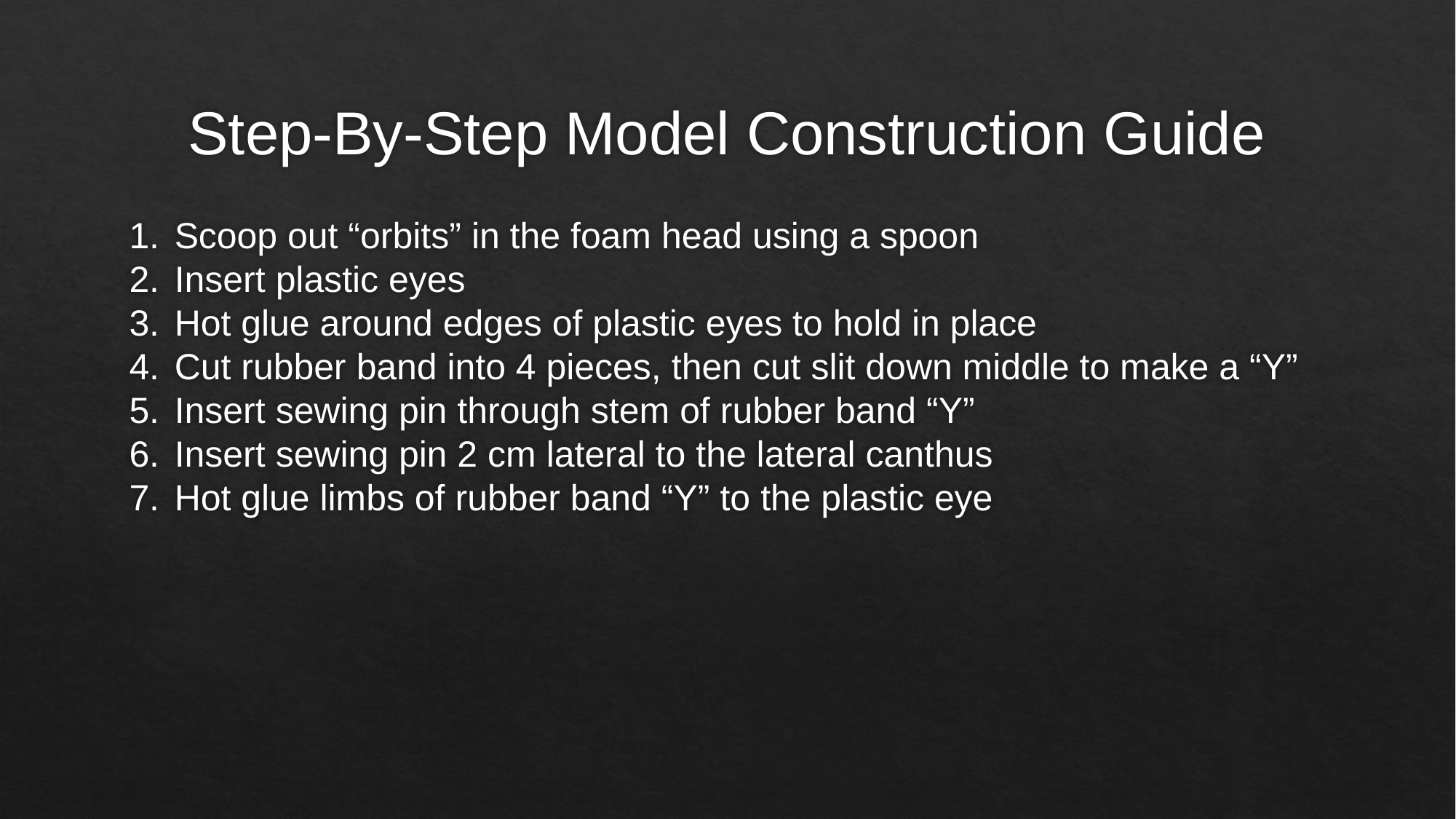

# Step-By-Step Model Construction Guide
Scoop out “orbits” in the foam head using a spoon
Insert plastic eyes
Hot glue around edges of plastic eyes to hold in place
Cut rubber band into 4 pieces, then cut slit down middle to make a “Y”
Insert sewing pin through stem of rubber band “Y”
Insert sewing pin 2 cm lateral to the lateral canthus
Hot glue limbs of rubber band “Y” to the plastic eye

## Slide 19
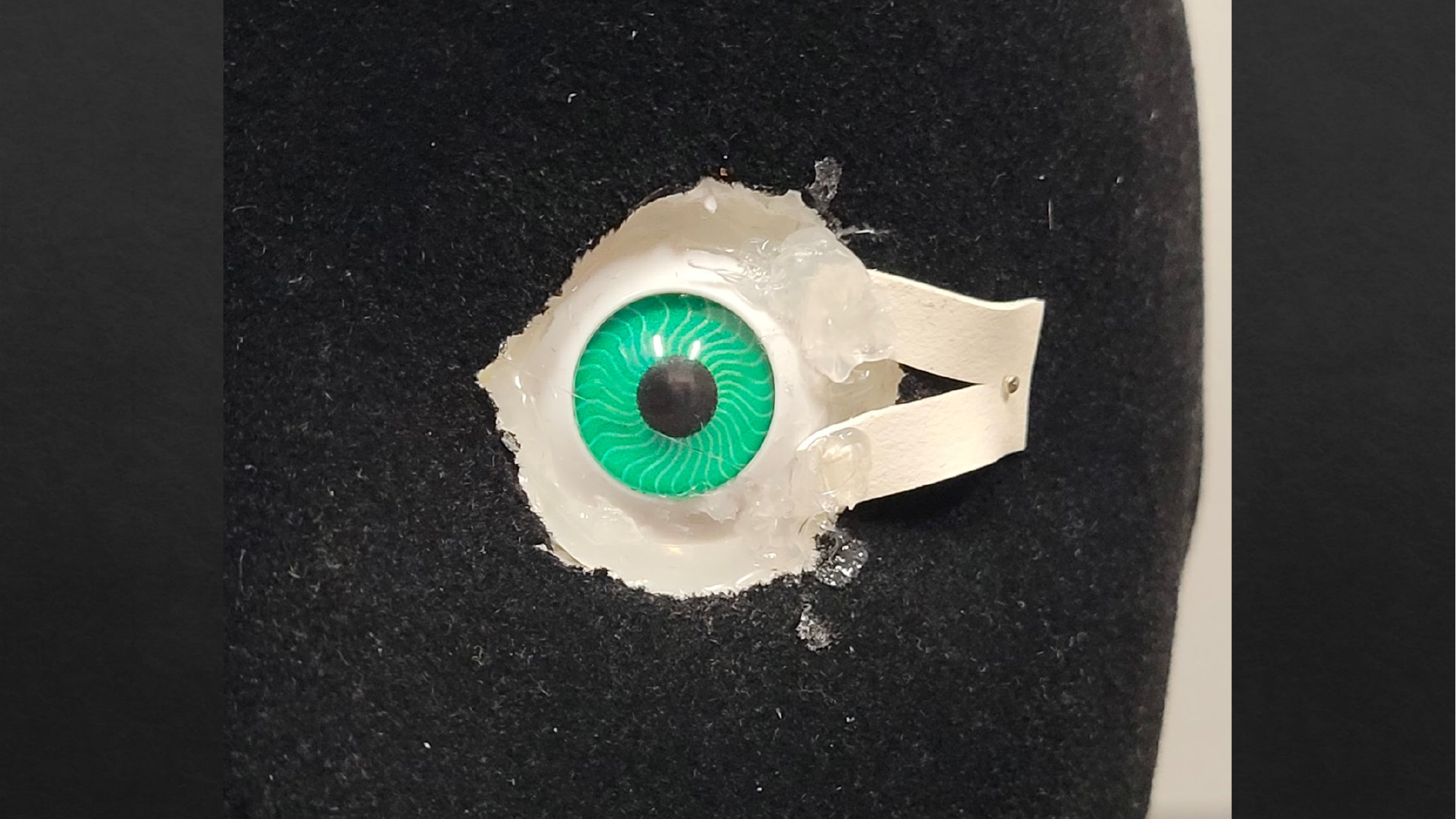

## Slide 20
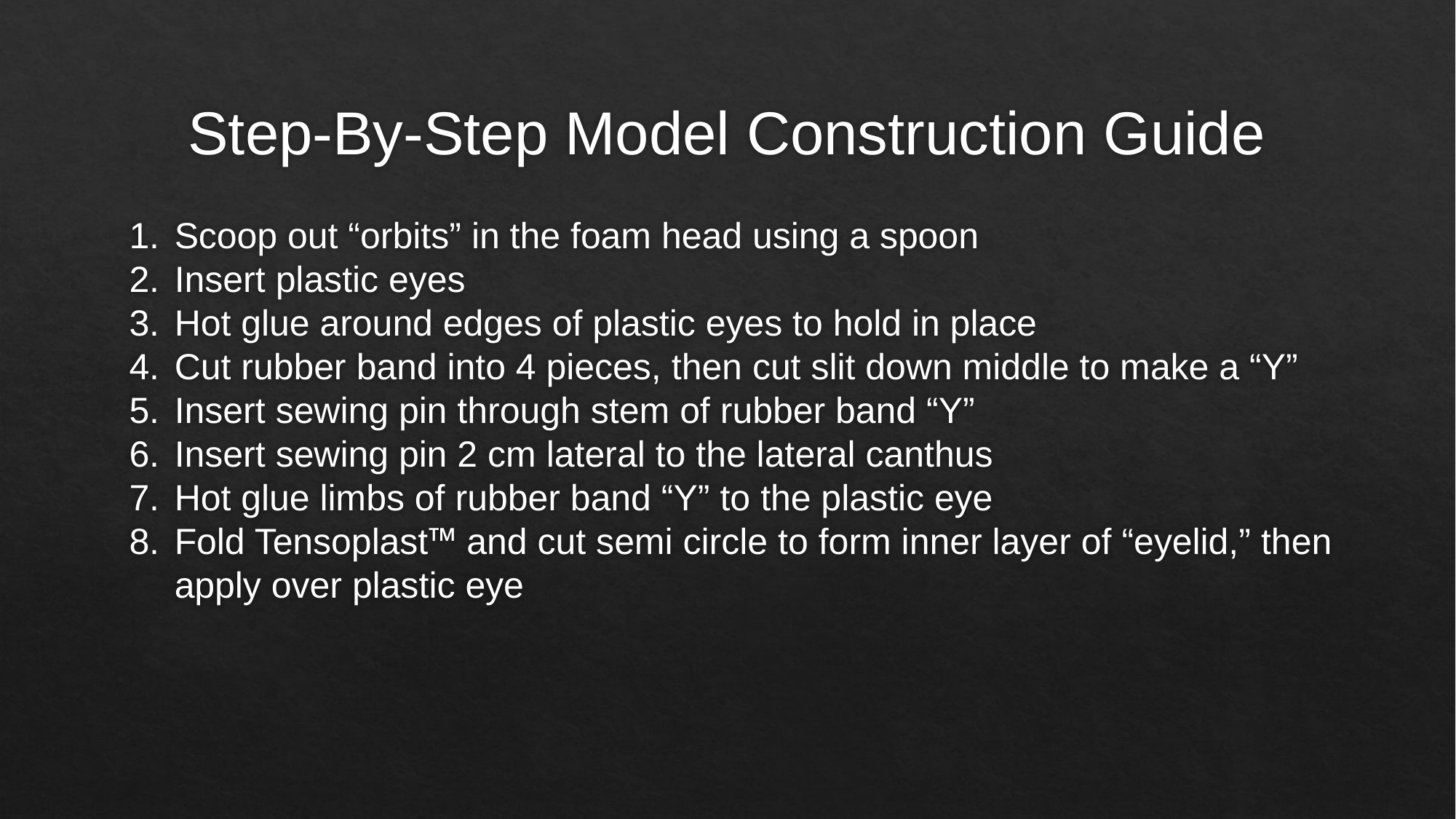

# Step-By-Step Model Construction Guide
Scoop out “orbits” in the foam head using a spoon
Insert plastic eyes
Hot glue around edges of plastic eyes to hold in place
Cut rubber band into 4 pieces, then cut slit down middle to make a “Y”
Insert sewing pin through stem of rubber band “Y”
Insert sewing pin 2 cm lateral to the lateral canthus
Hot glue limbs of rubber band “Y” to the plastic eye
Fold Tensoplast™ and cut semi circle to form inner layer of “eyelid,” then apply over plastic eye

## Slide 21
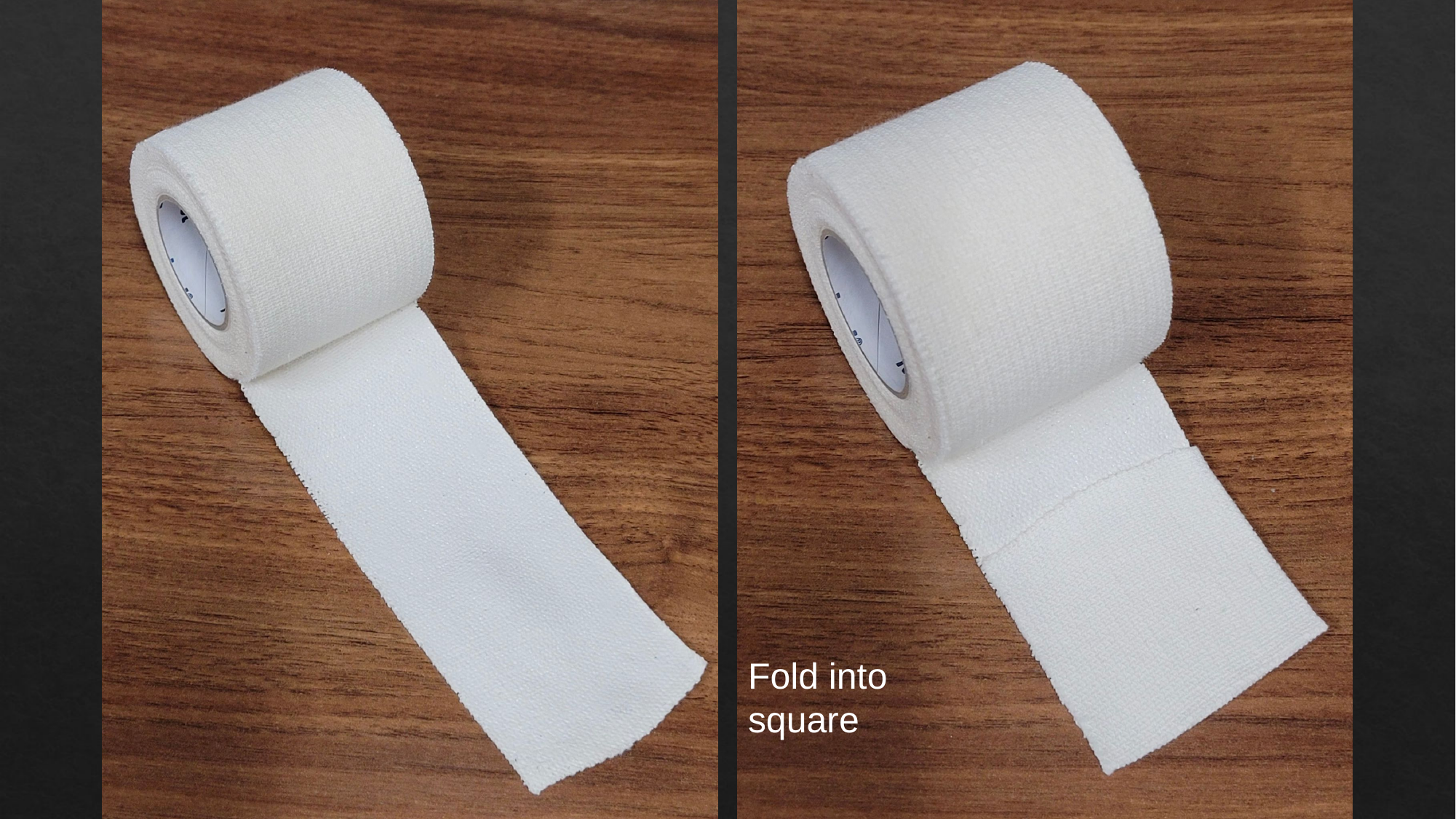

Fold into
square

## Slide 22
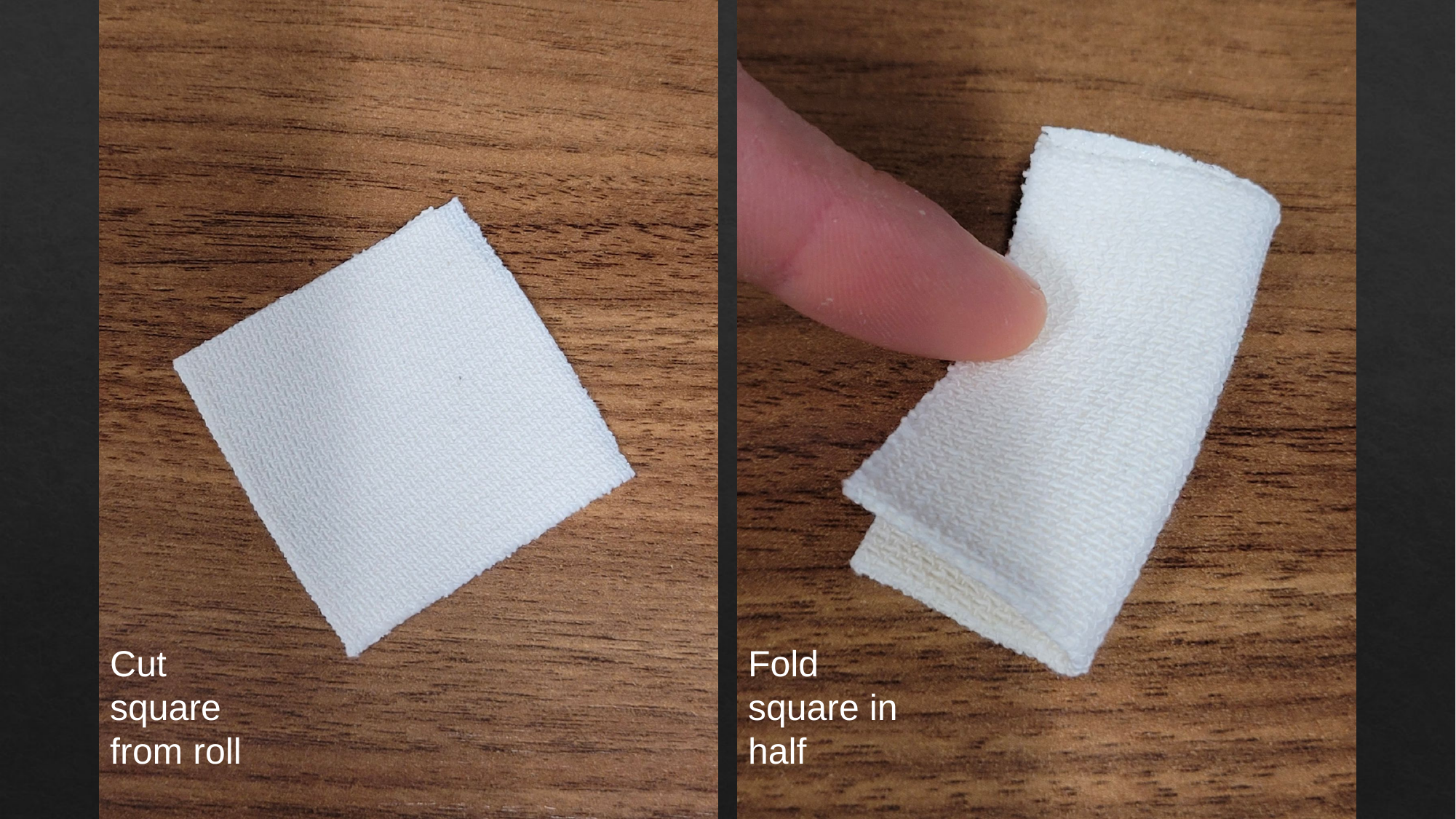

Cut
square from roll
Fold
square in half

## Slide 23
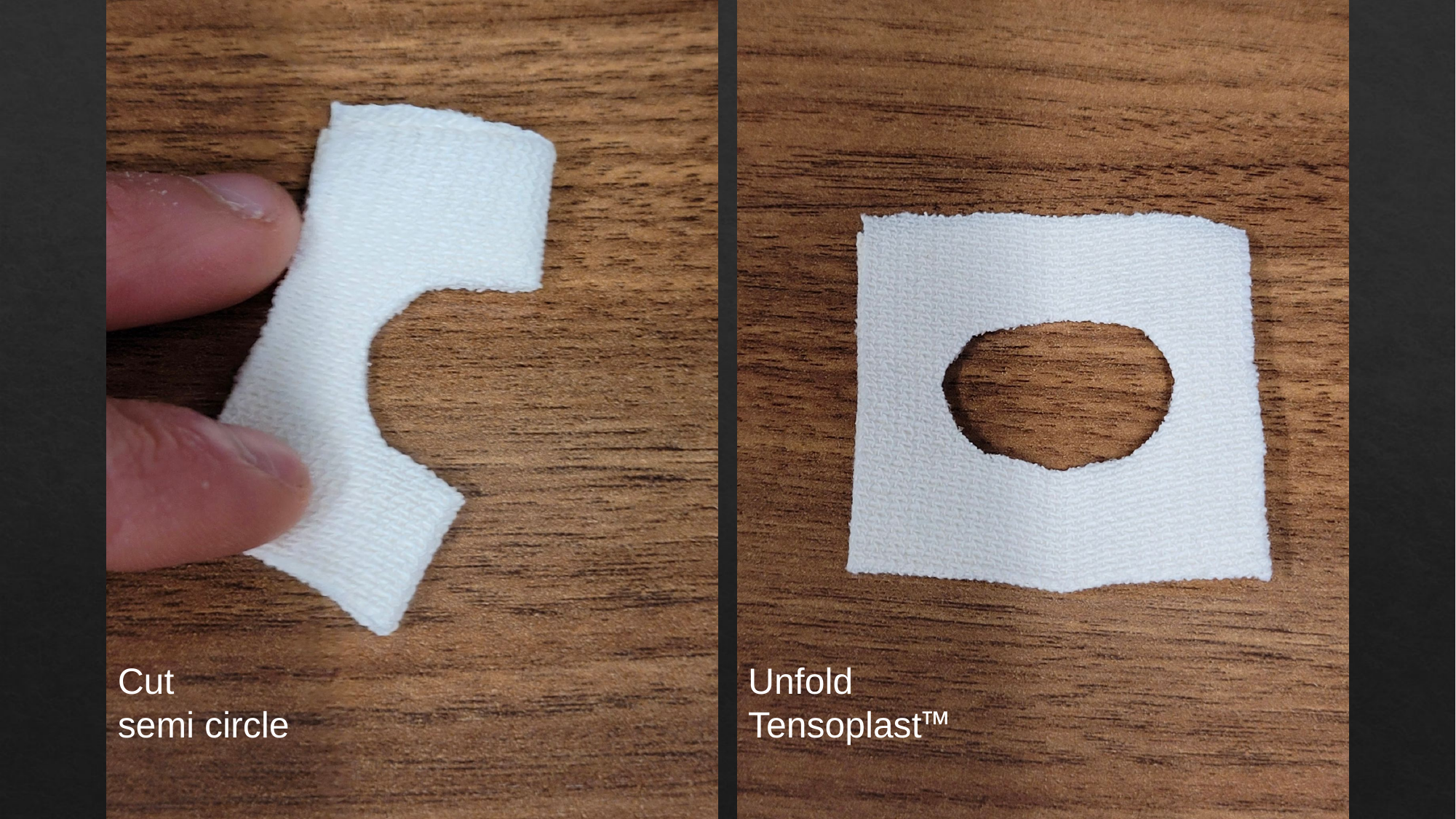

Cut
semi circle
Unfold
Tensoplast™

## Slide 24
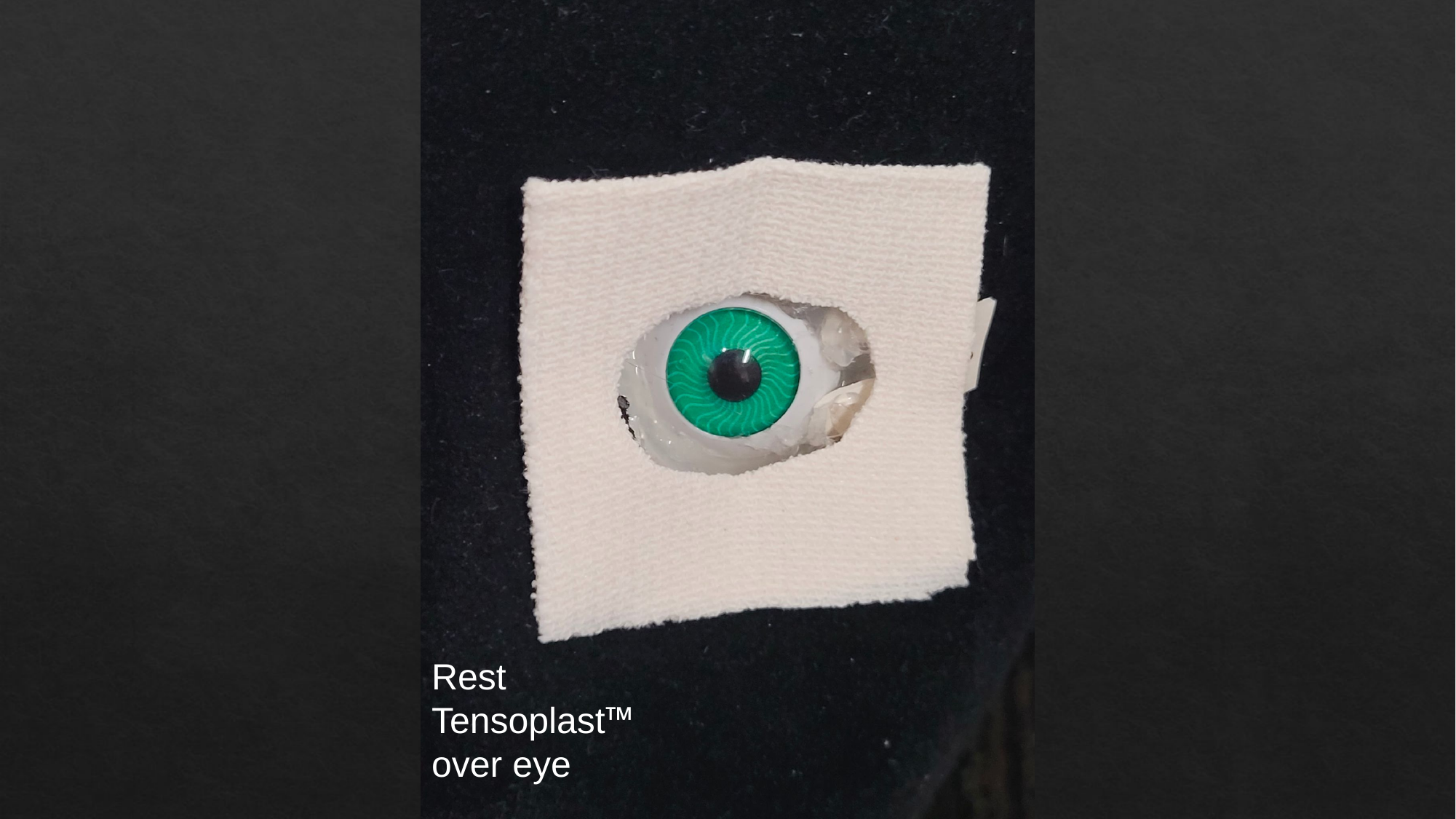

Rest
Tensoplast™
over eye

## Slide 25
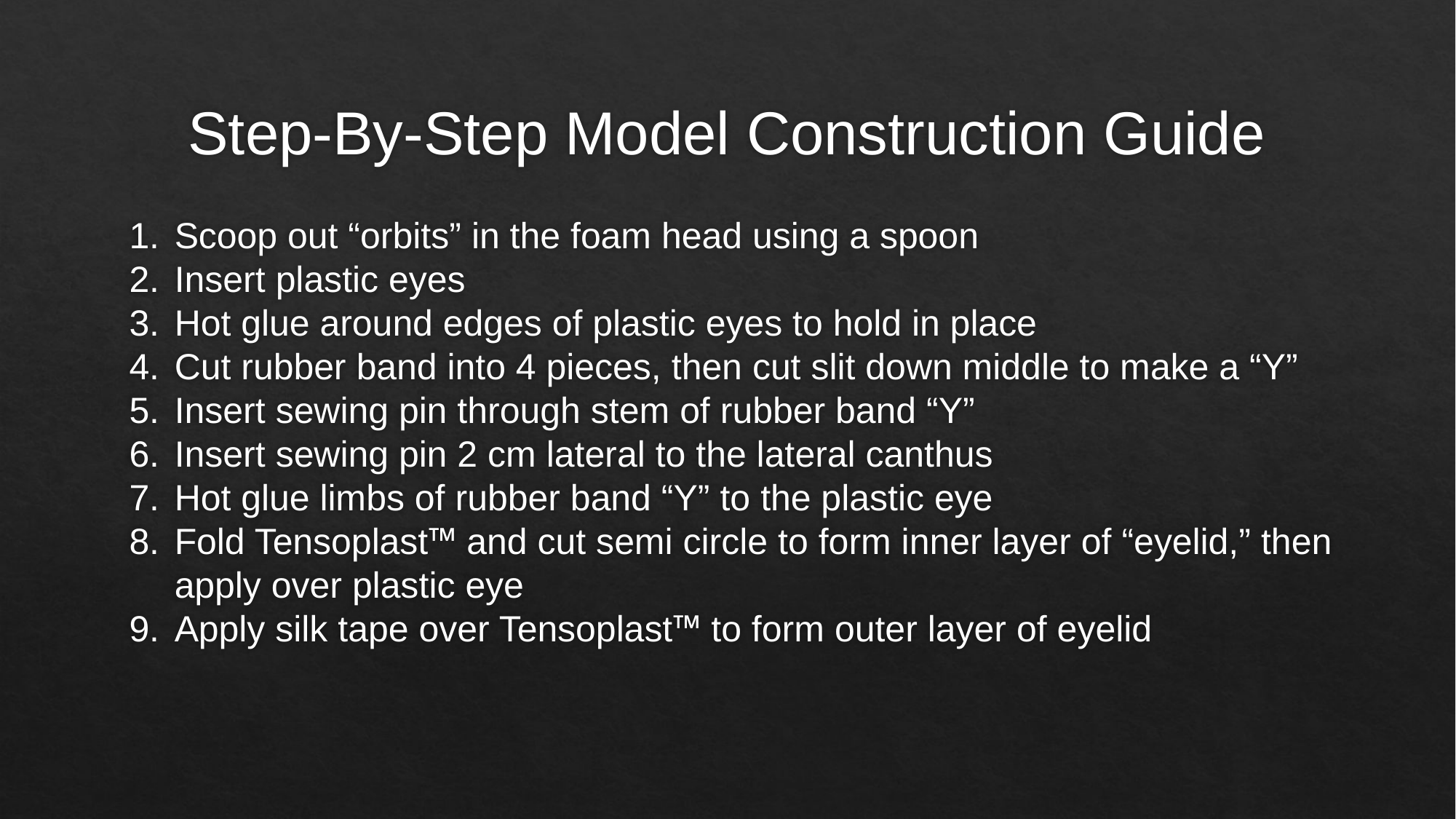

# Step-By-Step Model Construction Guide
Scoop out “orbits” in the foam head using a spoon
Insert plastic eyes
Hot glue around edges of plastic eyes to hold in place
Cut rubber band into 4 pieces, then cut slit down middle to make a “Y”
Insert sewing pin through stem of rubber band “Y”
Insert sewing pin 2 cm lateral to the lateral canthus
Hot glue limbs of rubber band “Y” to the plastic eye
Fold Tensoplast™ and cut semi circle to form inner layer of “eyelid,” then apply over plastic eye
Apply silk tape over Tensoplast™ to form outer layer of eyelid

## Slide 26
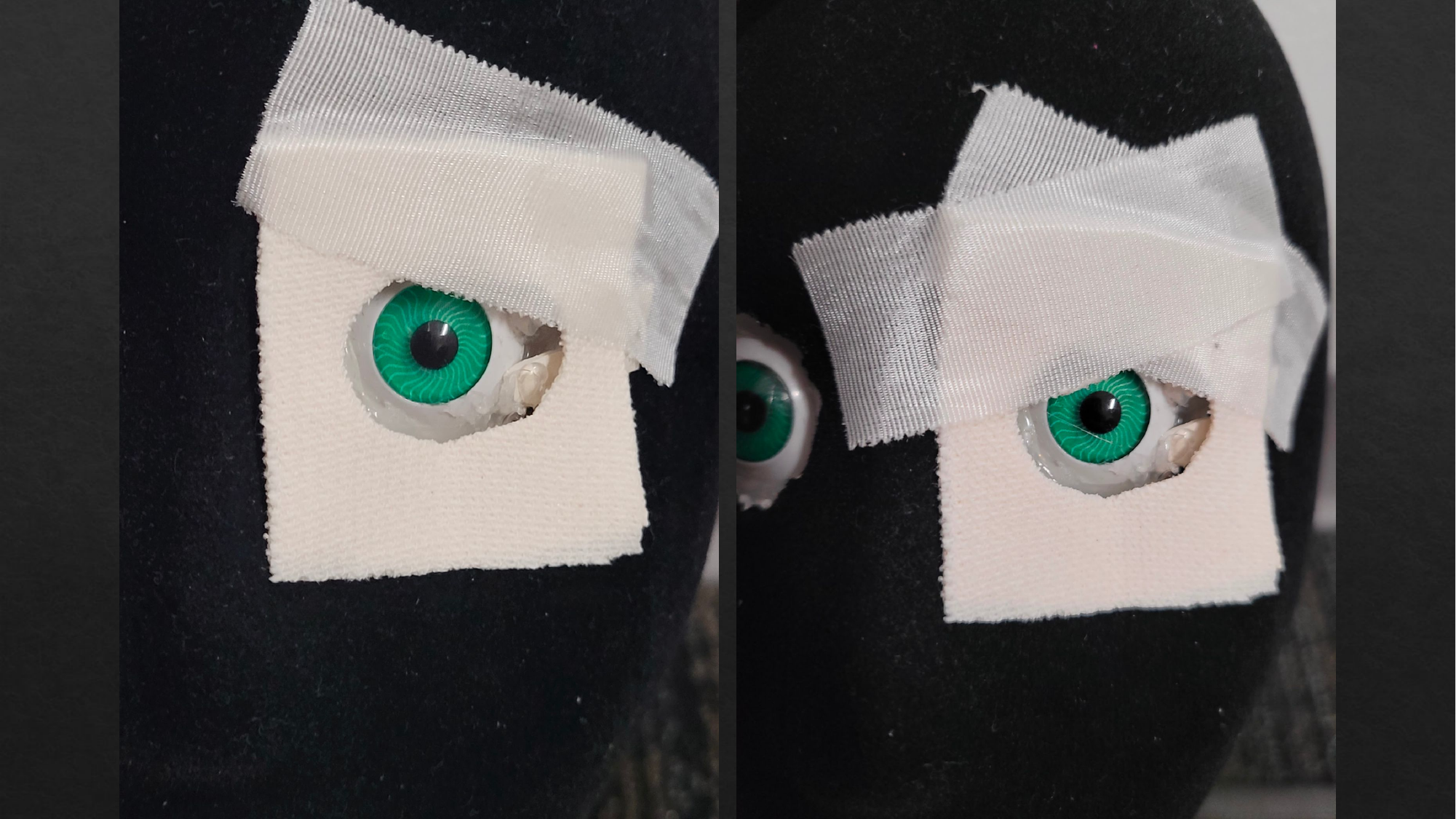

## Slide 27
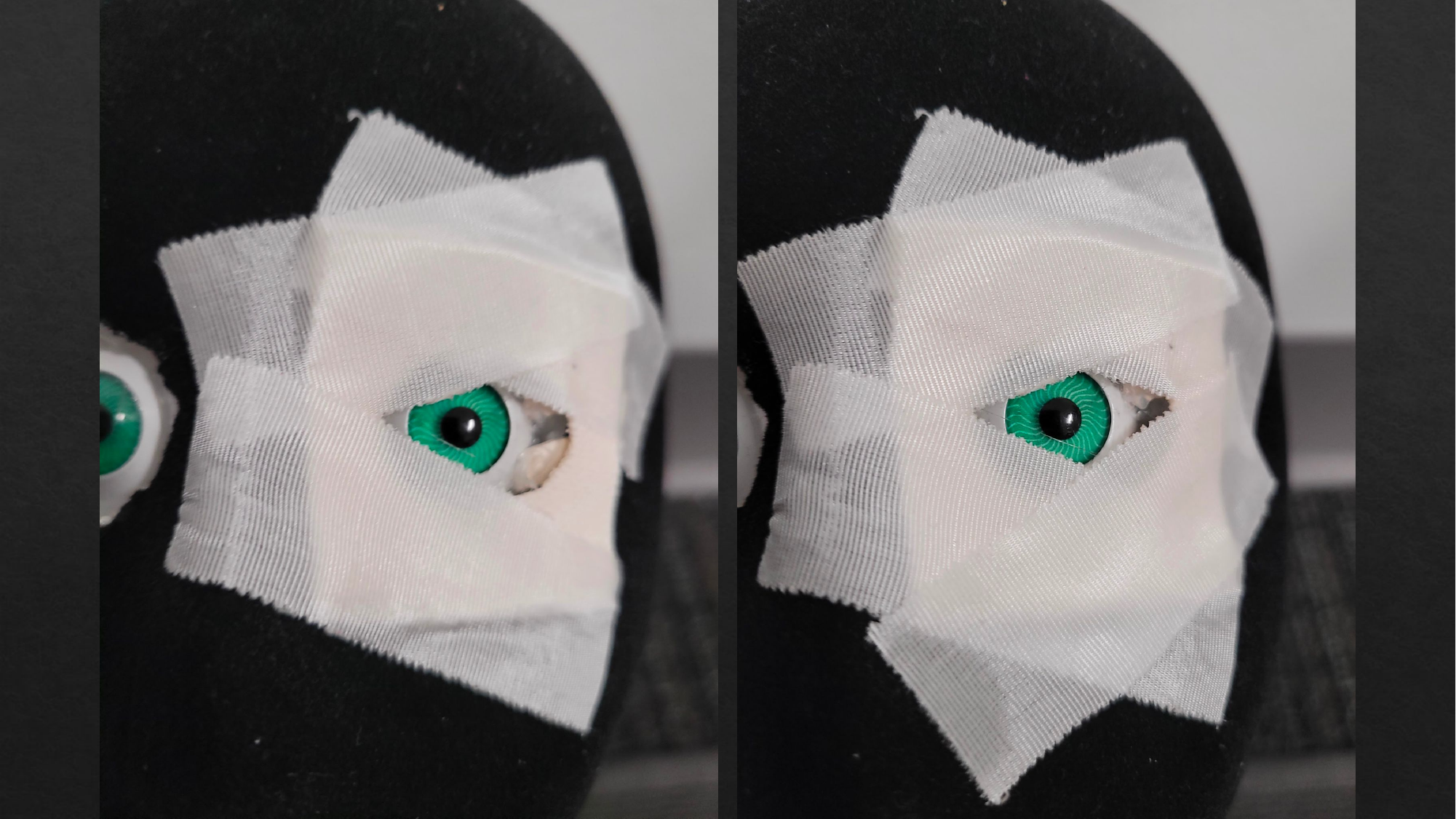

## Slide 28
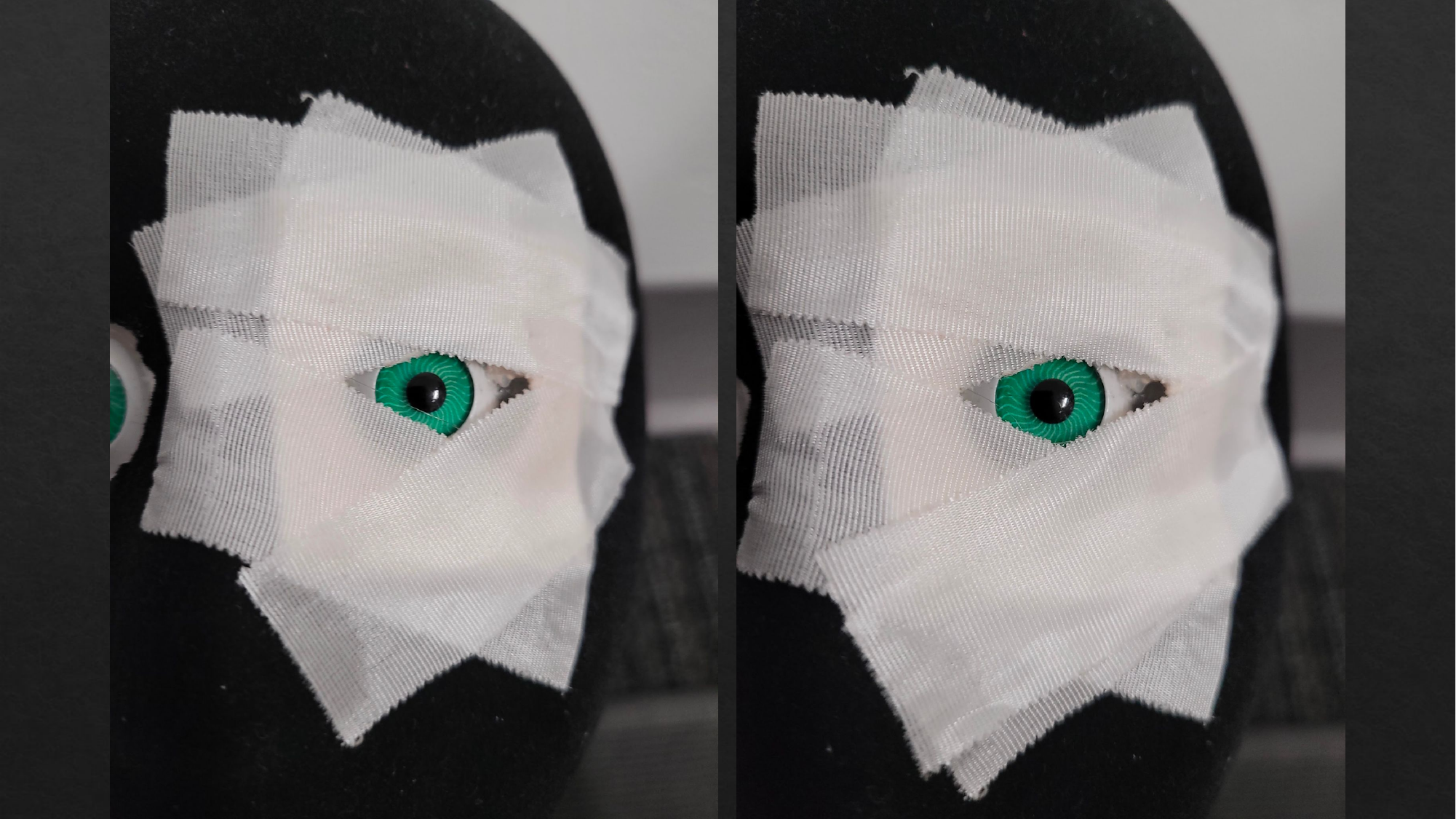

## Slide 29
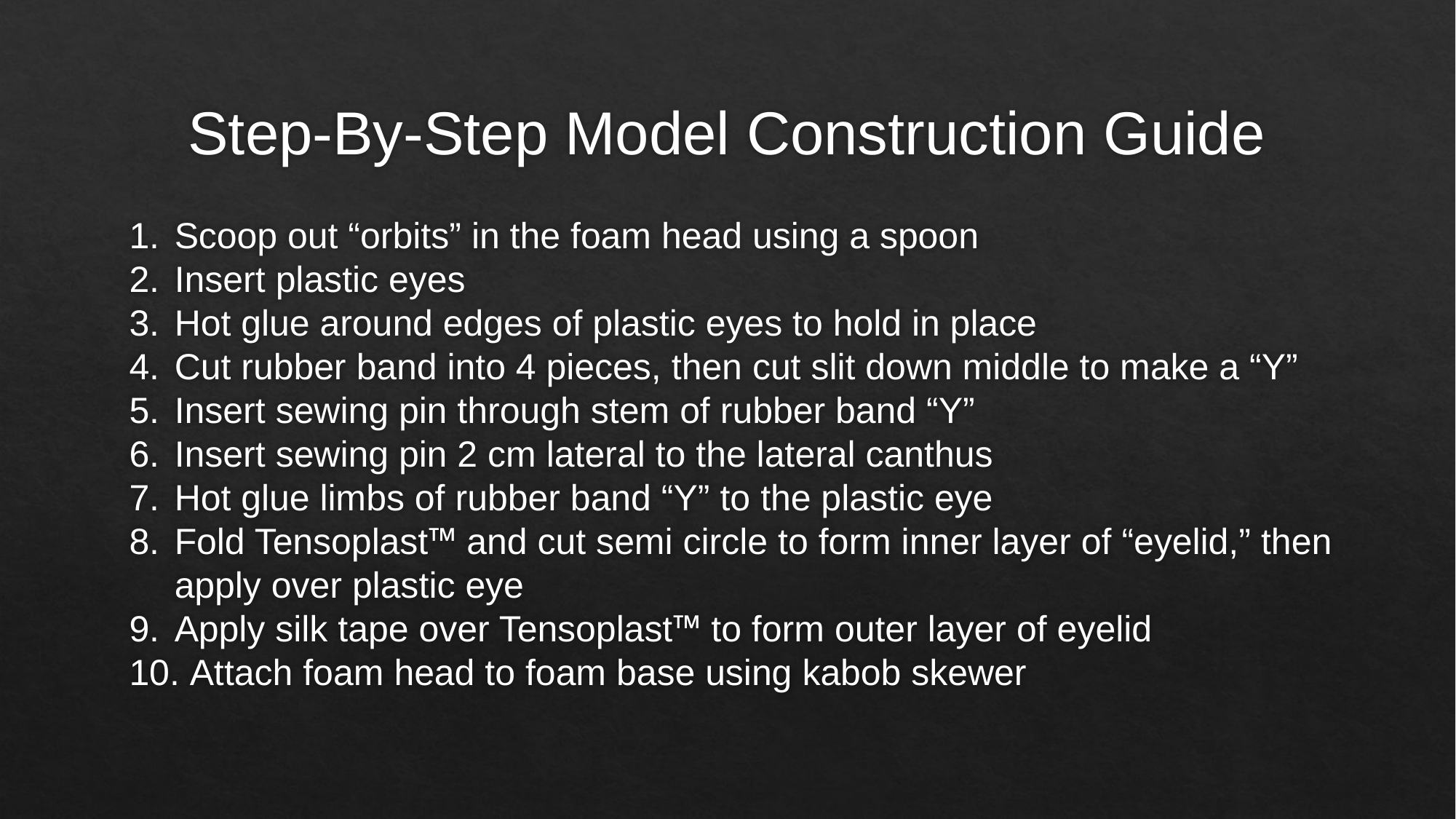

# Step-By-Step Model Construction Guide
Scoop out “orbits” in the foam head using a spoon
Insert plastic eyes
Hot glue around edges of plastic eyes to hold in place
Cut rubber band into 4 pieces, then cut slit down middle to make a “Y”
Insert sewing pin through stem of rubber band “Y”
Insert sewing pin 2 cm lateral to the lateral canthus
Hot glue limbs of rubber band “Y” to the plastic eye
Fold Tensoplast™ and cut semi circle to form inner layer of “eyelid,” then apply over plastic eye
Apply silk tape over Tensoplast™ to form outer layer of eyelid
 Attach foam head to foam base using kabob skewer

## Slide 30
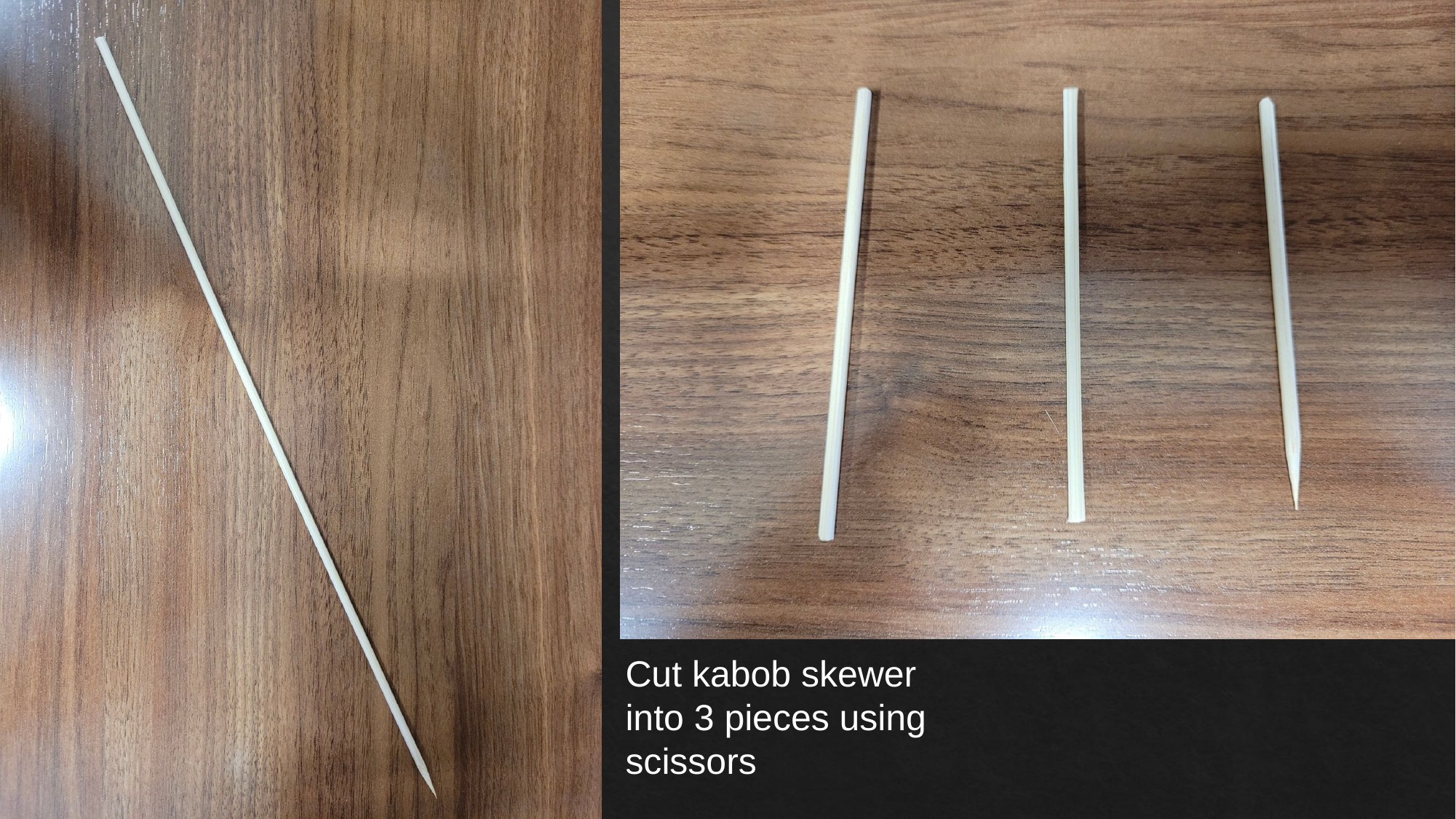

Cut kabob skewer into 3 pieces using scissors

## Slide 31
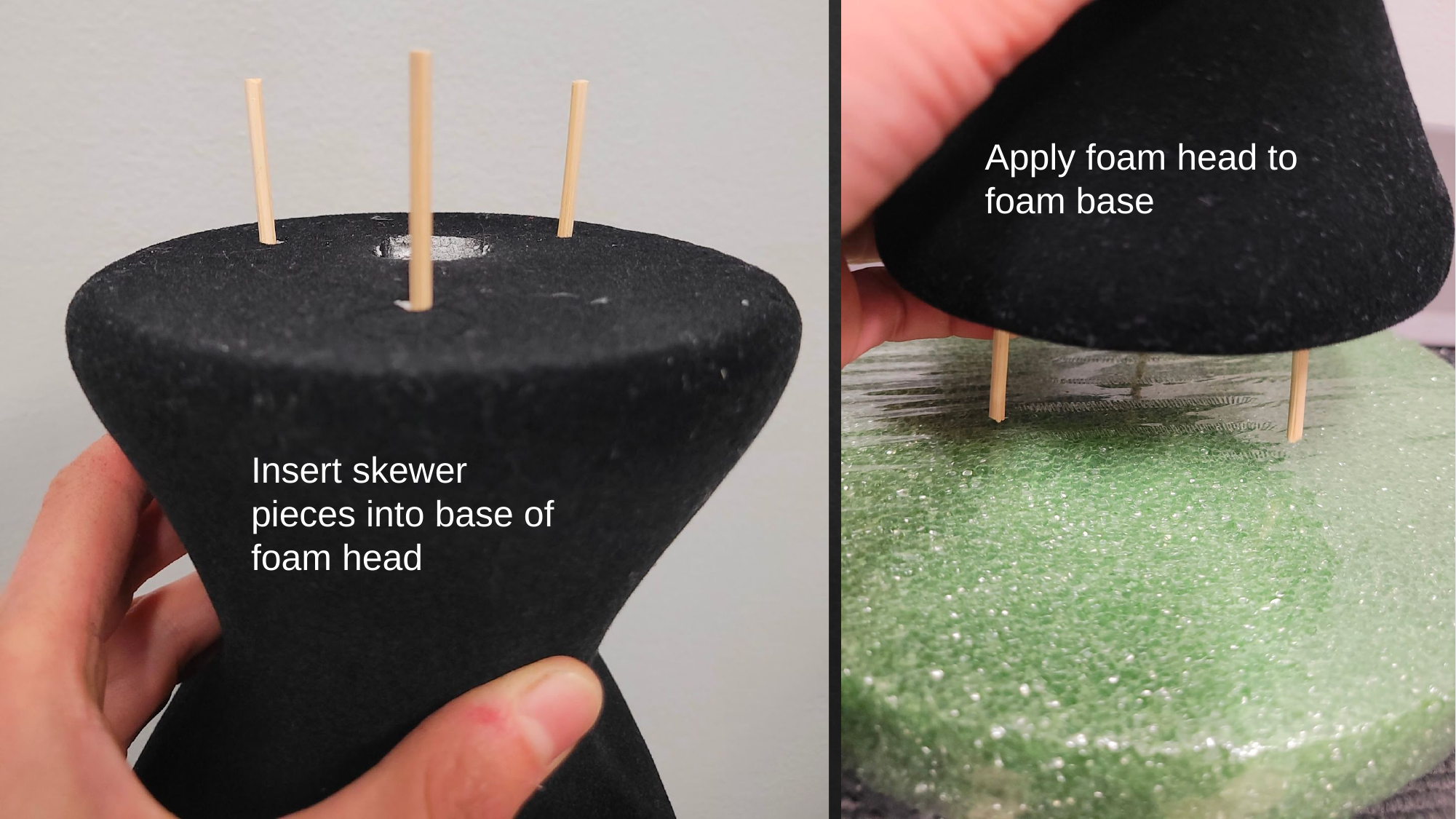

Apply foam head to foam base
Insert skewer pieces into base of foam head

## Slide 32
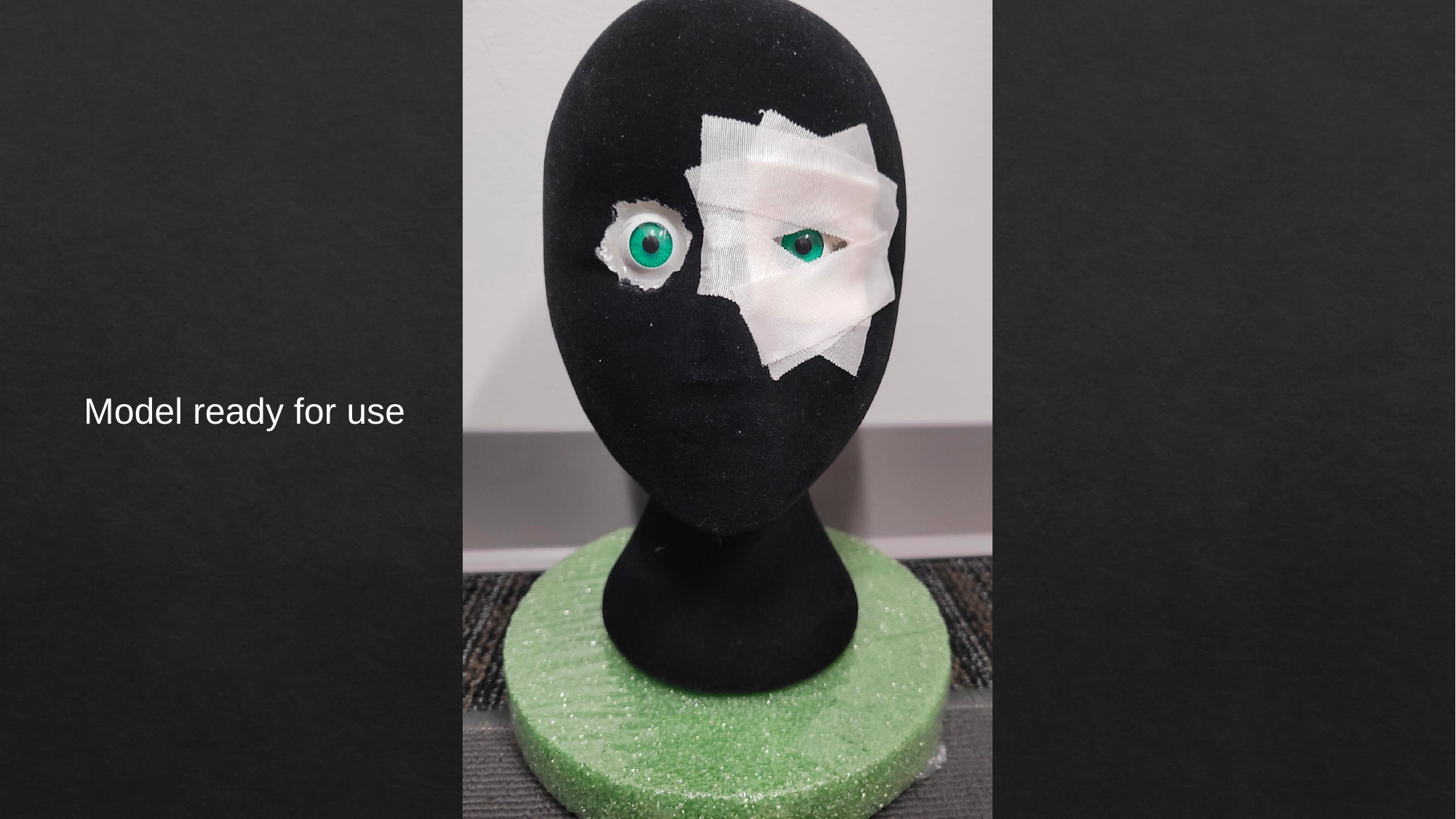

Model ready for use

## Slide 33
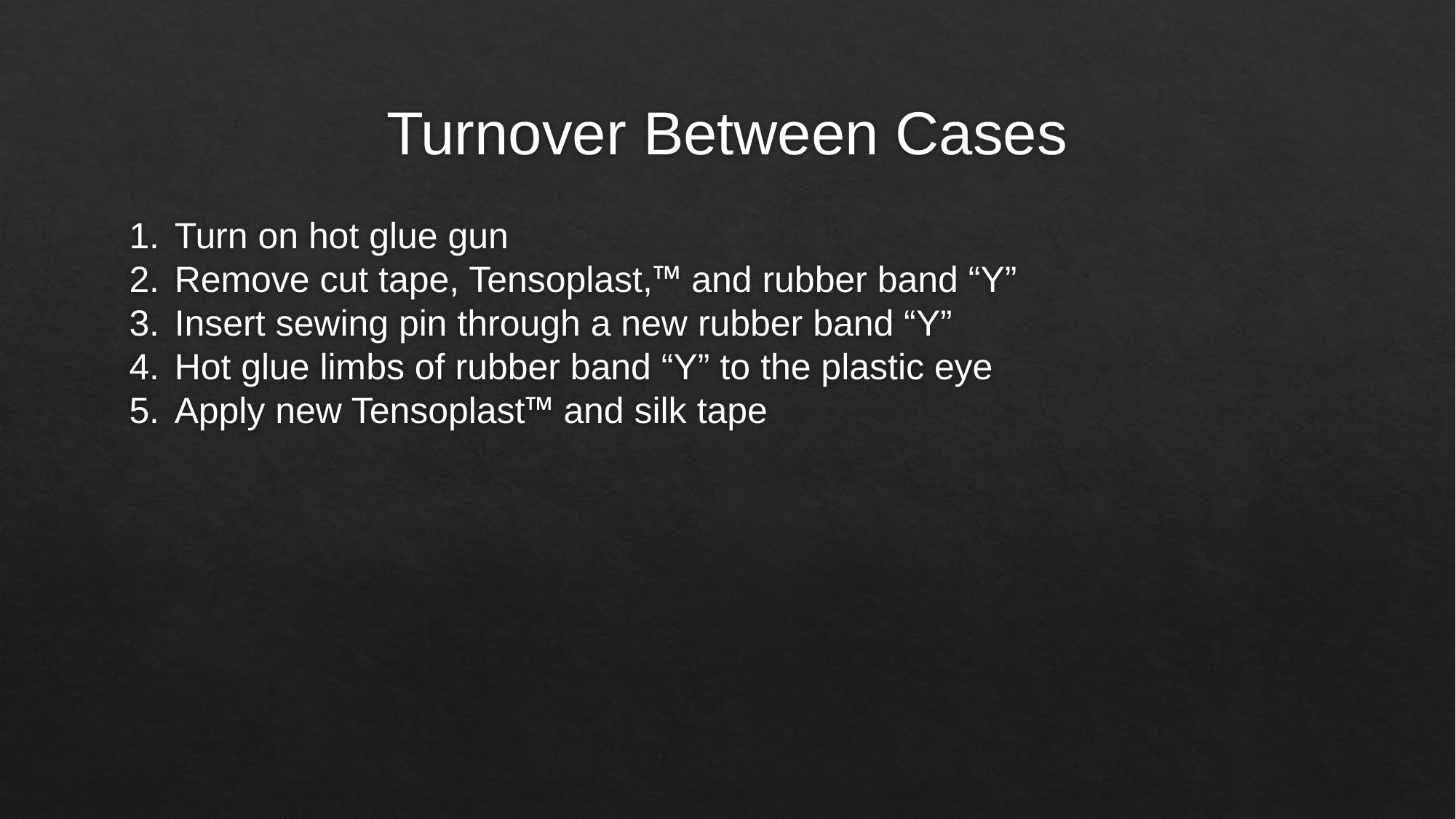

# Turnover Between Cases
Turn on hot glue gun
Remove cut tape, Tensoplast,™ and rubber band “Y”
Insert sewing pin through a new rubber band “Y”
Hot glue limbs of rubber band “Y” to the plastic eye
Apply new Tensoplast™ and silk tape

## Slide 34
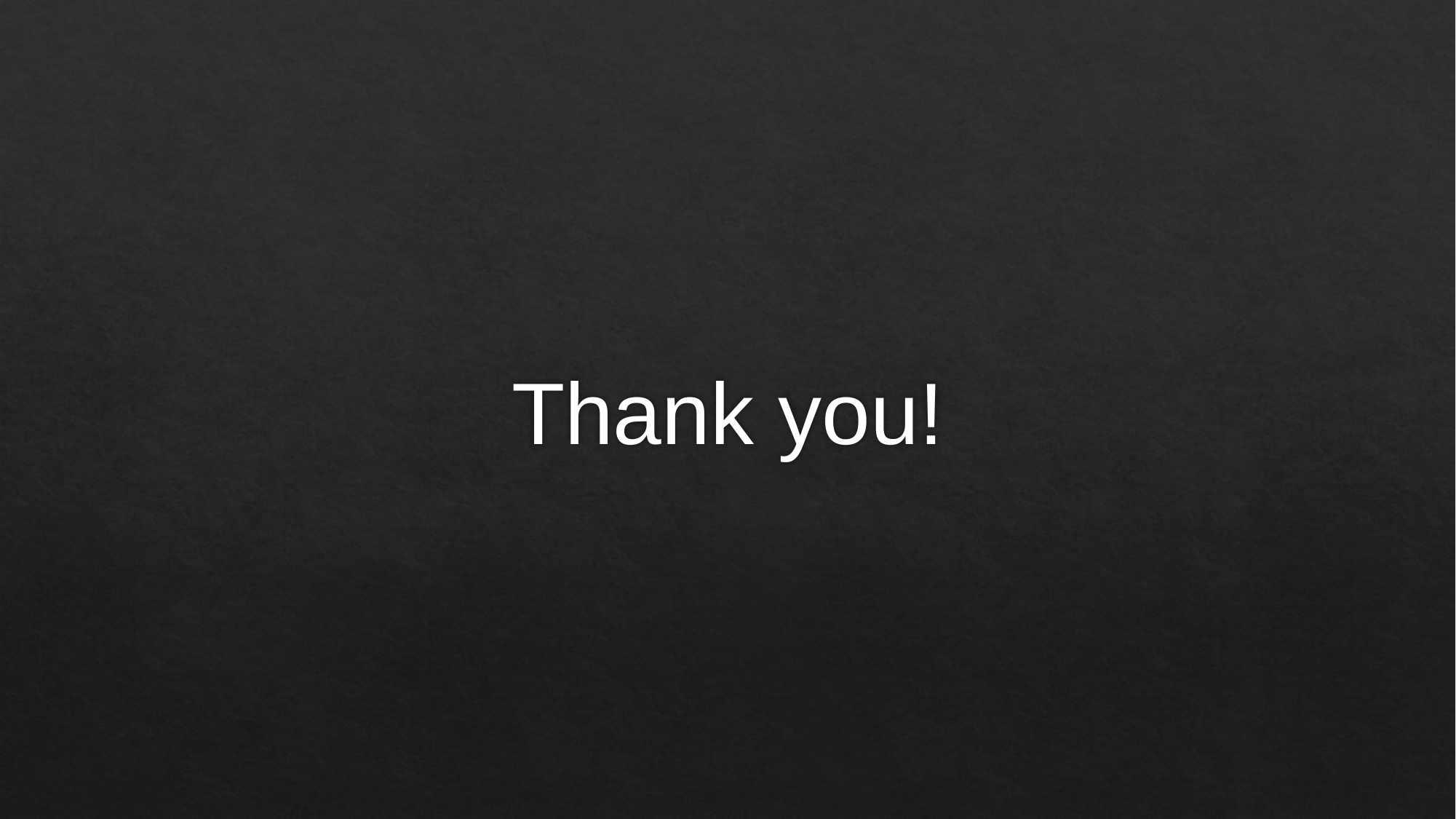

# Thank you!
