# Supplementary material for: A Case of Painful Visual Loss - Managing Orbital Compartment Syndrome in the Emergency Department
Source: J Educ Teach Emerg Med. 2024 Oct 31;9(4):S1–S50. doi: 10.21980/J8N35D (PMC11537727; doi:10.21980/J8N35D)
Supplement: Supplementary file 2 — Please see associated PowerPoint file [file 9-4-S1-Appendix_B.pptx]

## Slide 1
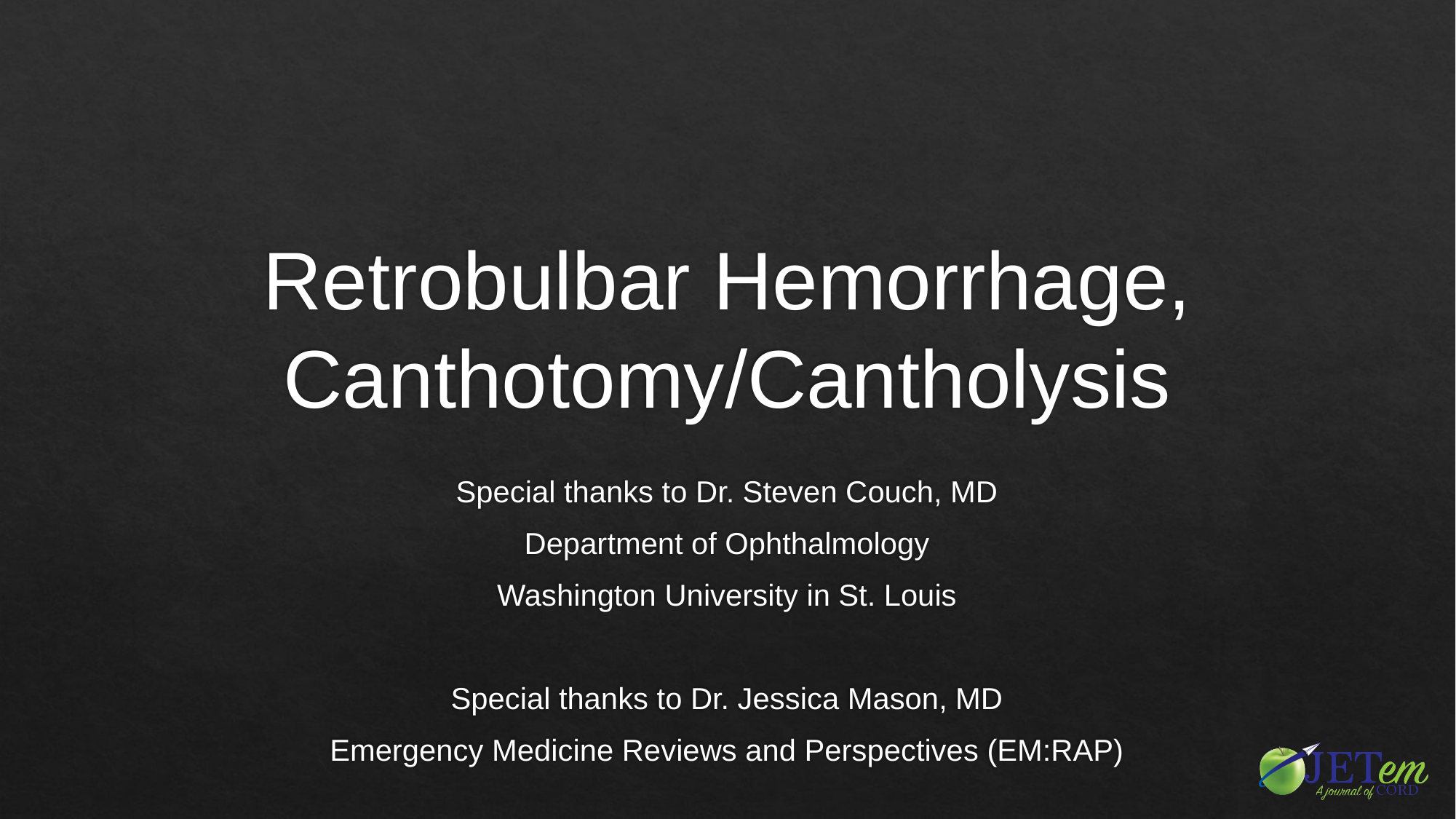

# Retrobulbar Hemorrhage, Canthotomy/Cantholysis
Special thanks to Dr. Steven Couch, MD
Department of Ophthalmology
Washington University in St. Louis
Special thanks to Dr. Jessica Mason, MD
Emergency Medicine Reviews and Perspectives (EM:RAP)

## Slide 2
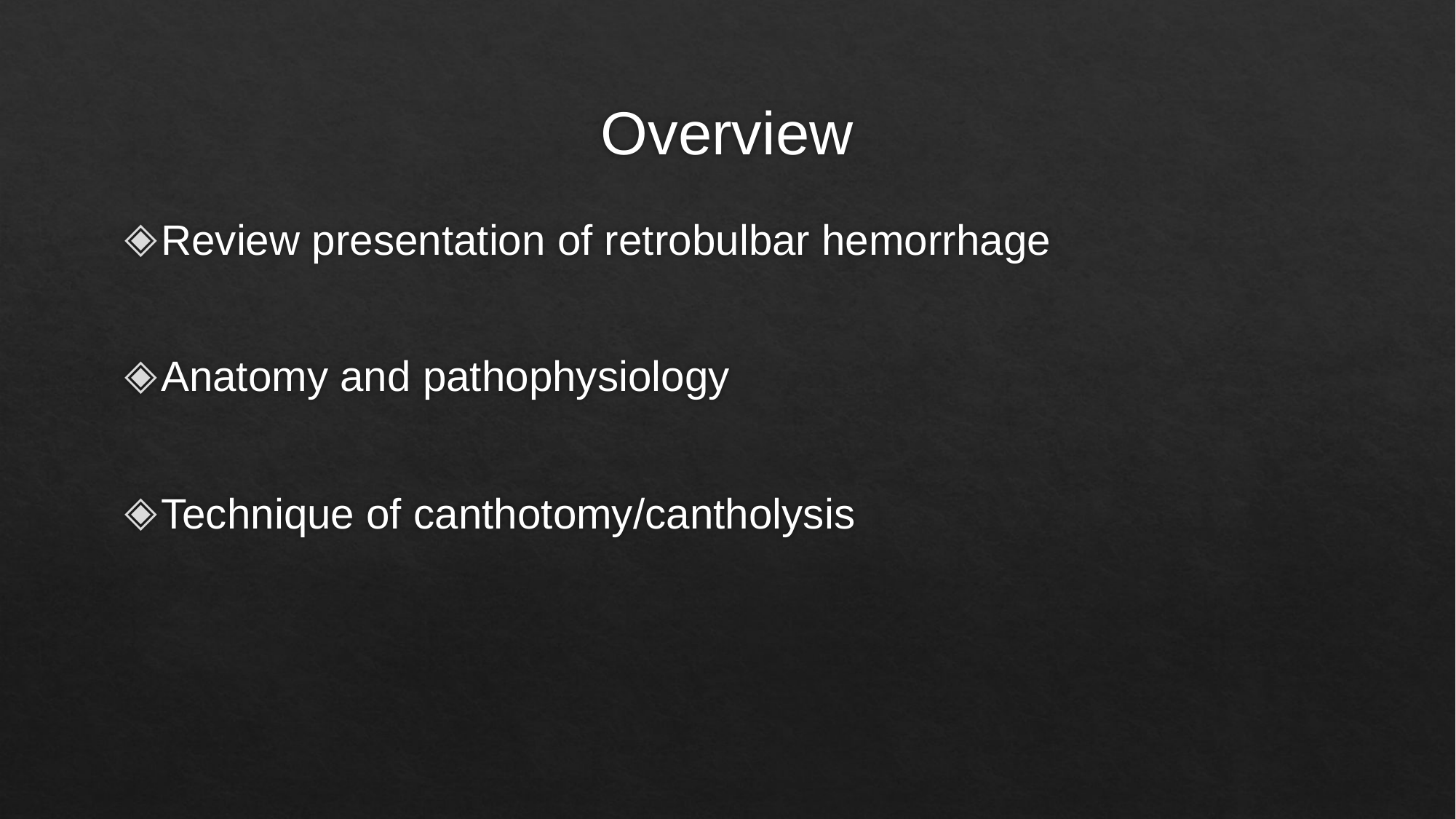

# Overview
Review presentation of retrobulbar hemorrhage
Anatomy and pathophysiology
Technique of canthotomy/cantholysis

## Slide 3
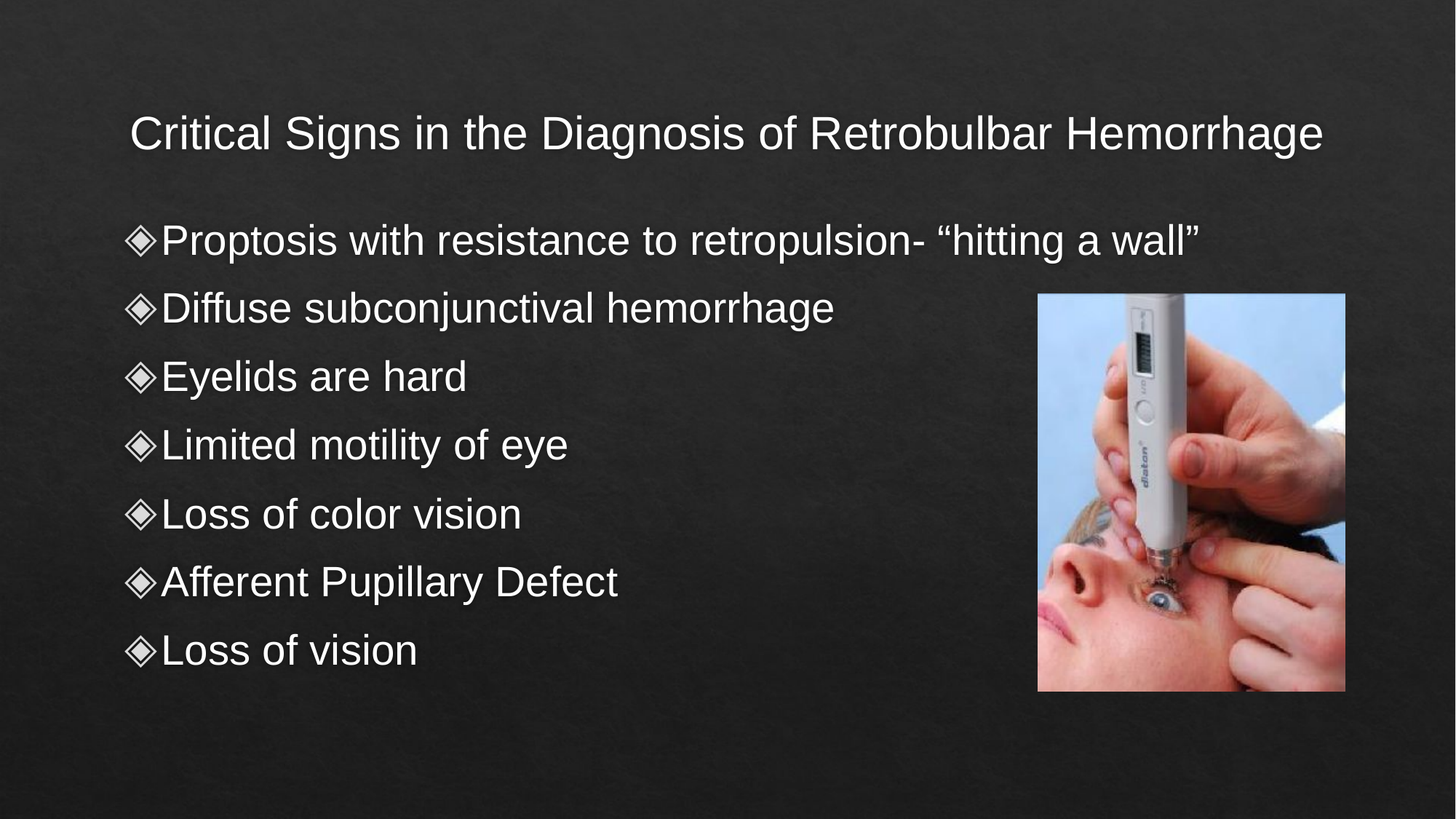

# Critical Signs in the Diagnosis of Retrobulbar Hemorrhage
Proptosis with resistance to retropulsion- “hitting a wall”
Diffuse subconjunctival hemorrhage
Eyelids are hard
Limited motility of eye
Loss of color vision
Afferent Pupillary Defect
Loss of vision

## Slide 4
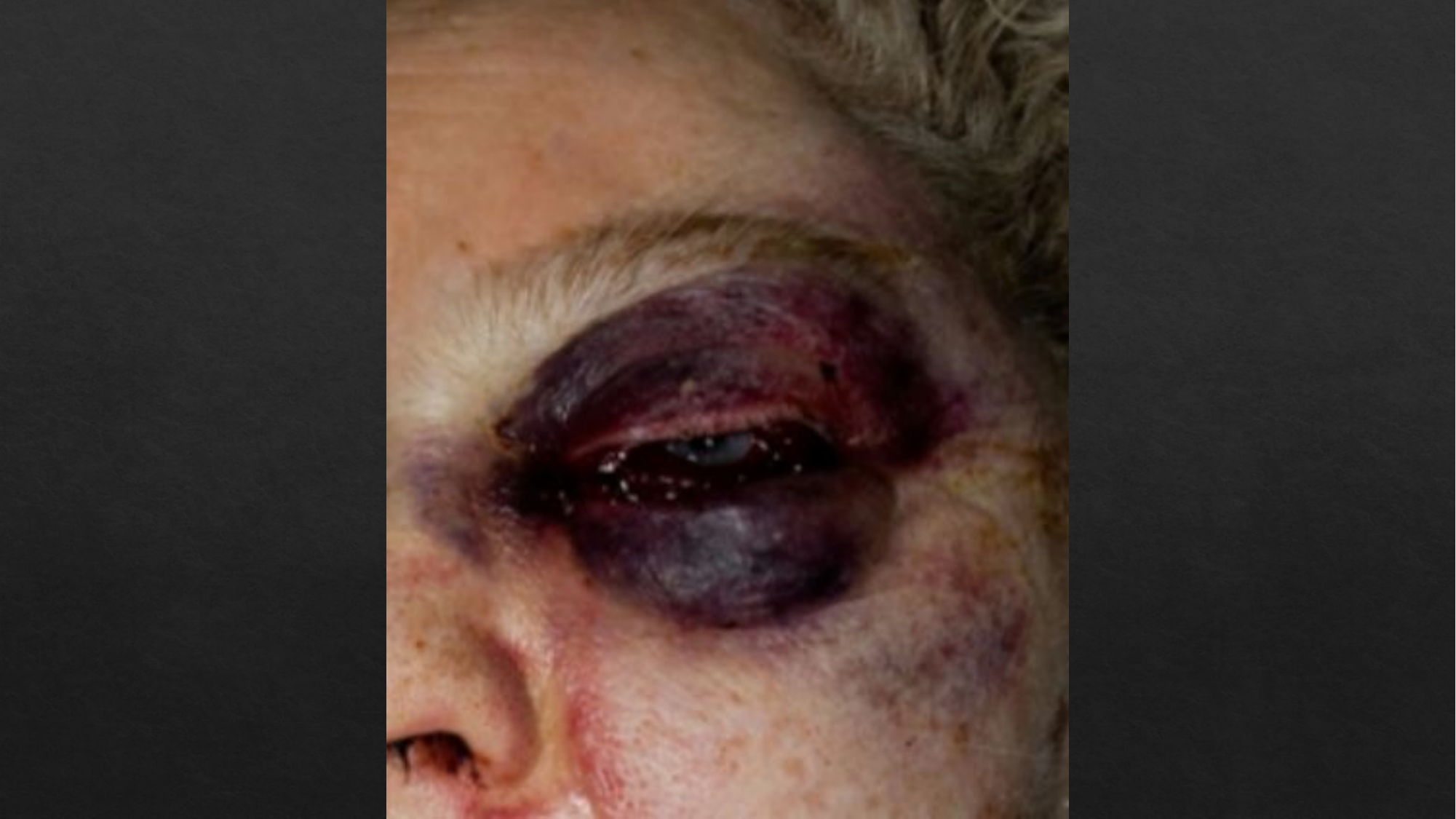

## Slide 5
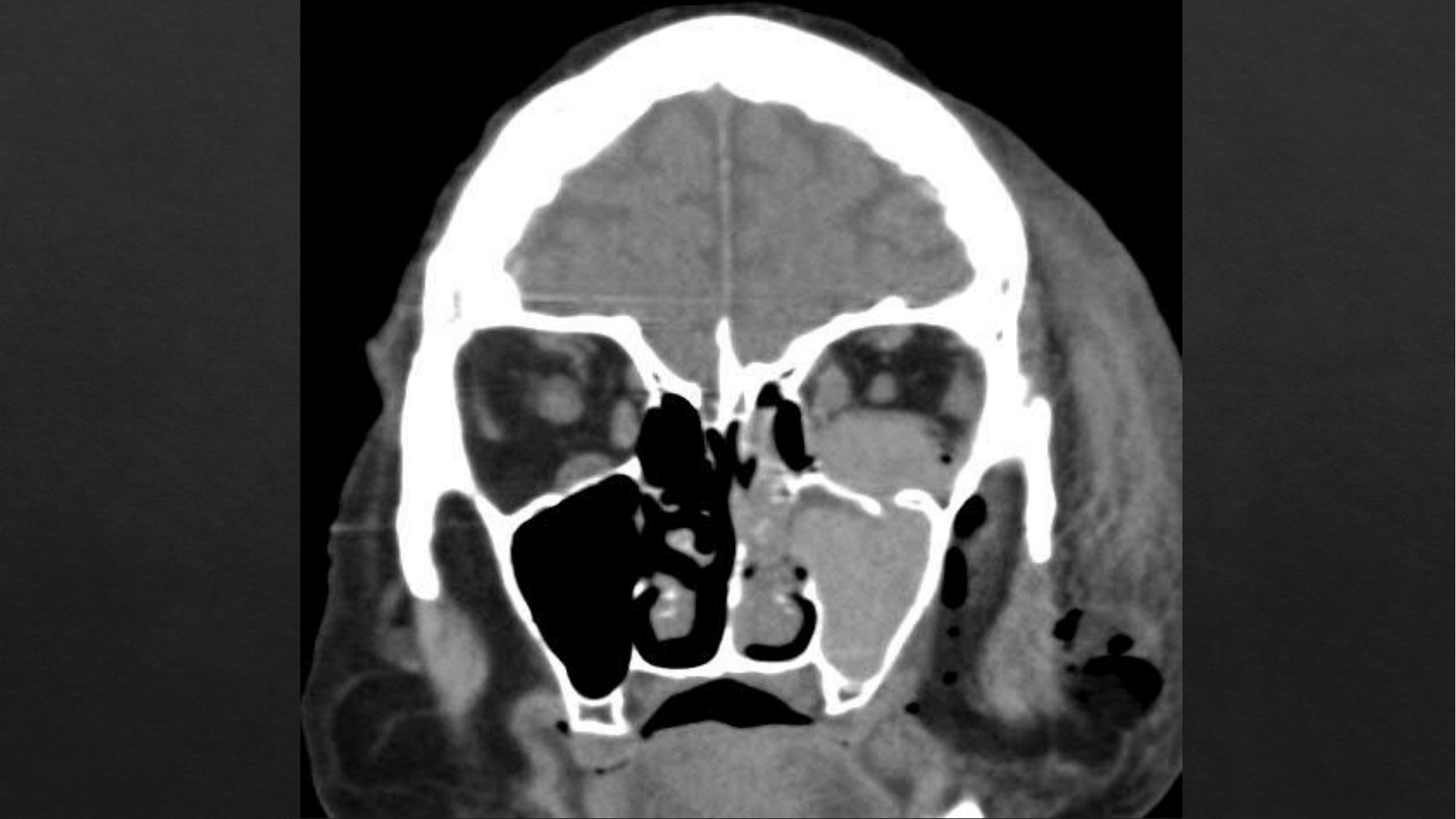

## Slide 6
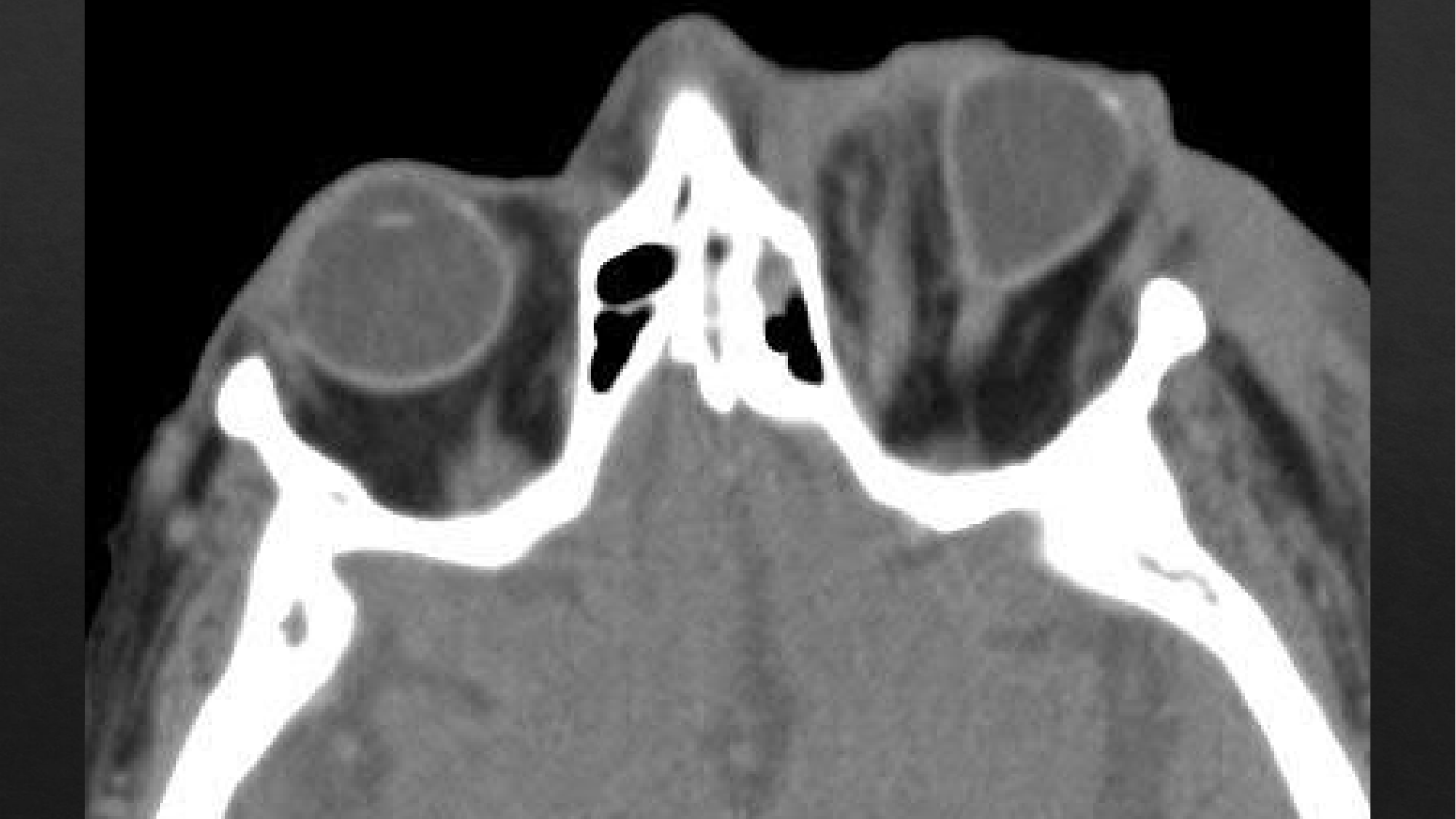

## Slide 7
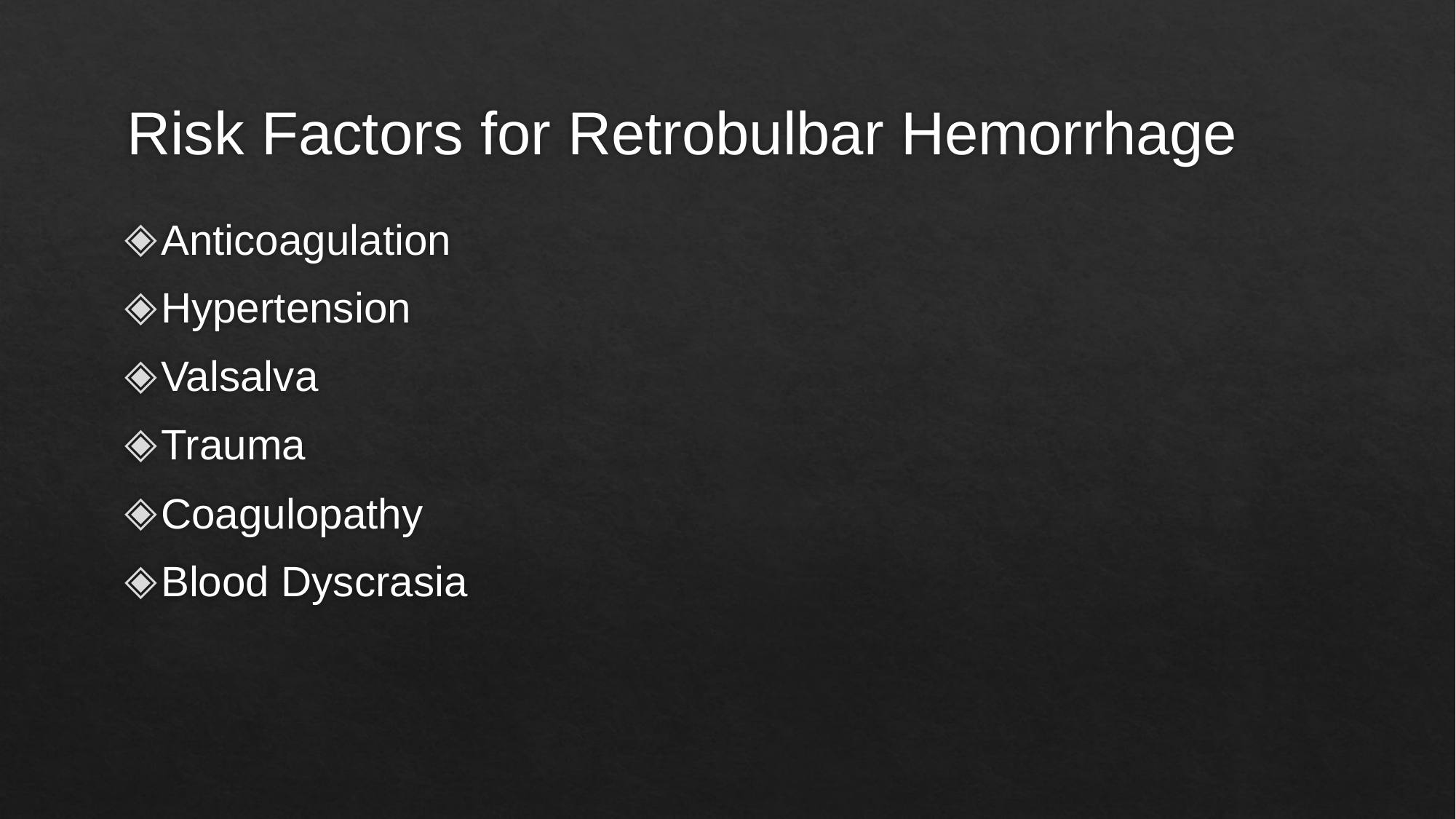

# Risk Factors for Retrobulbar Hemorrhage
Anticoagulation
Hypertension
Valsalva
Trauma
Coagulopathy
Blood Dyscrasia

## Slide 8
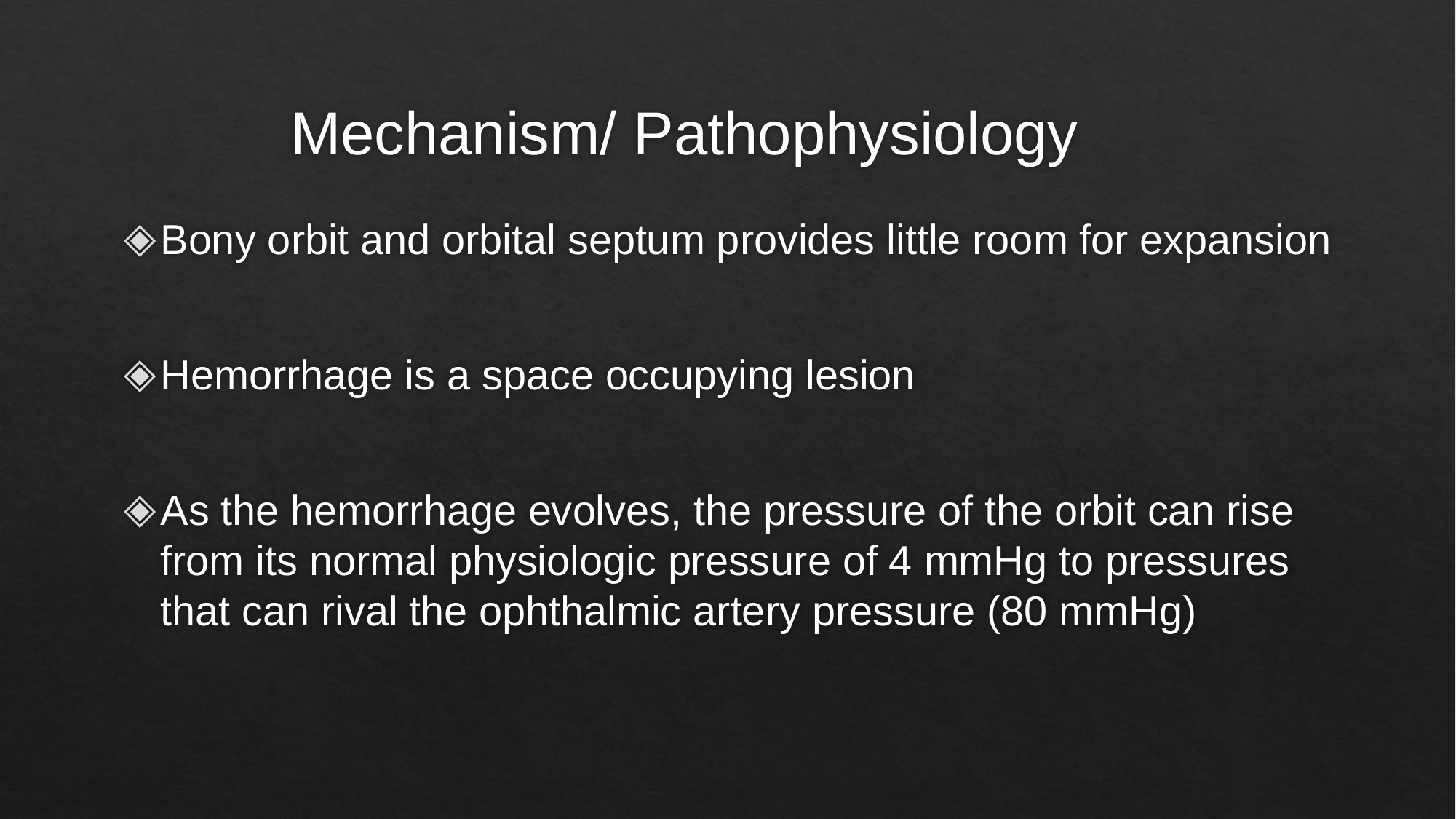

# Mechanism/ Pathophysiology
Bony orbit and orbital septum provides little room for expansion
Hemorrhage is a space occupying lesion
As the hemorrhage evolves, the pressure of the orbit can rise from its normal physiologic pressure of 4 mmHg to pressures that can rival the ophthalmic artery pressure (80 mmHg)

## Slide 9
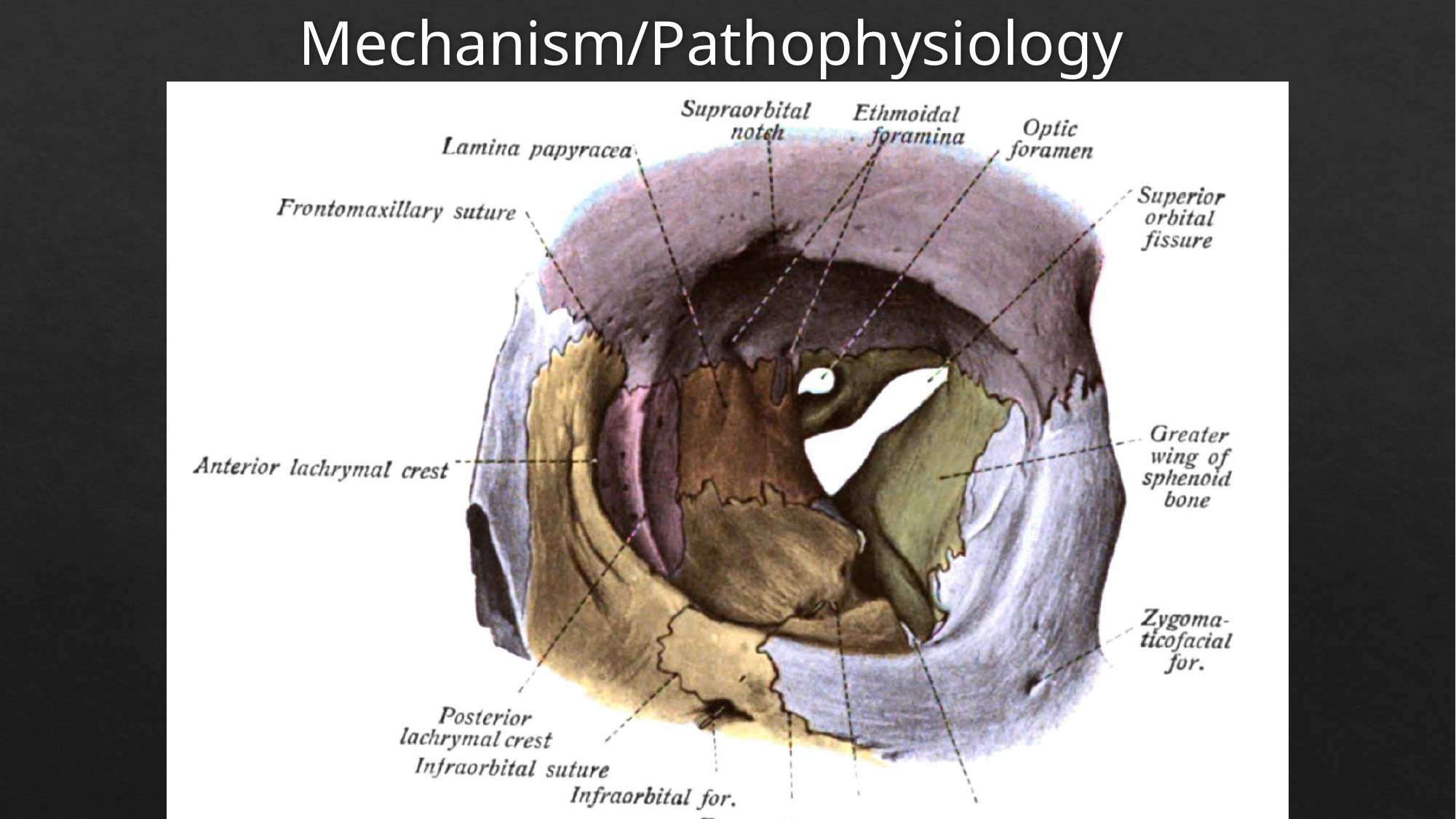

# Mechanism/Pathophysiology

## Slide 10
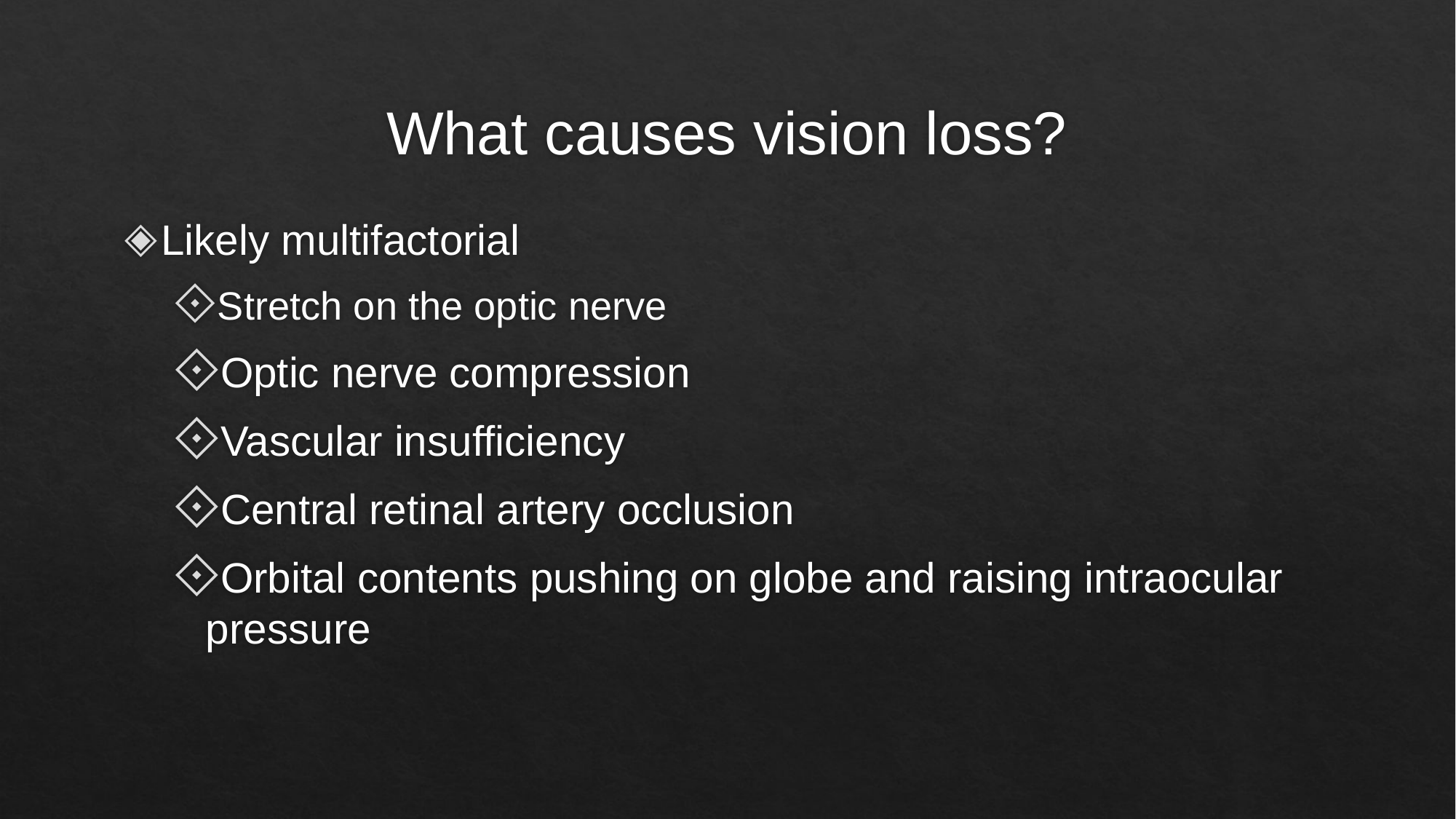

# What causes vision loss?
Likely multifactorial
Stretch on the optic nerve
Optic nerve compression
Vascular insufficiency
Central retinal artery occlusion
Orbital contents pushing on globe and raising intraocular pressure

## Slide 11
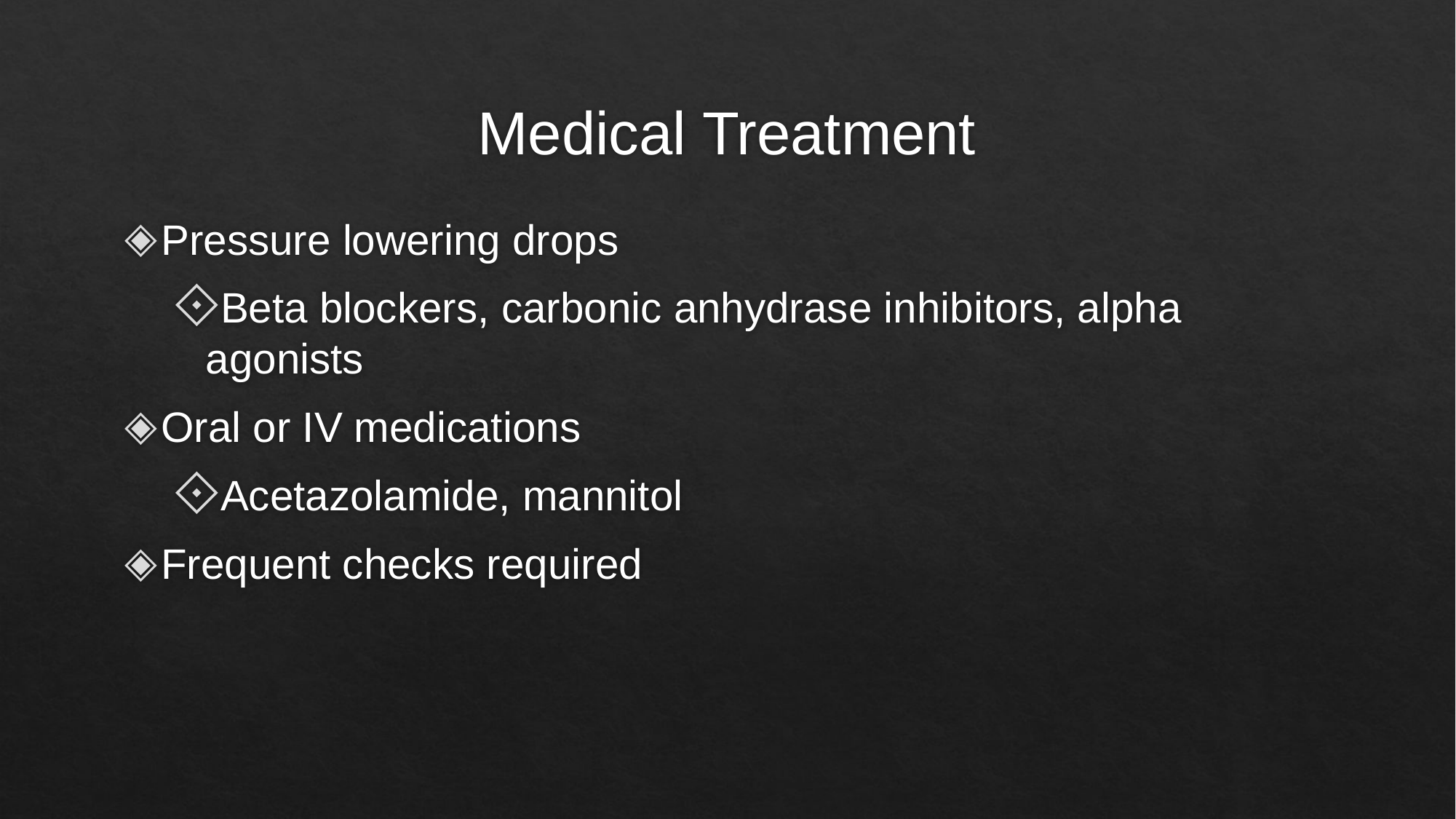

# Medical Treatment
Pressure lowering drops
Beta blockers, carbonic anhydrase inhibitors, alpha agonists
Oral or IV medications
Acetazolamide, mannitol
Frequent checks required

## Slide 12
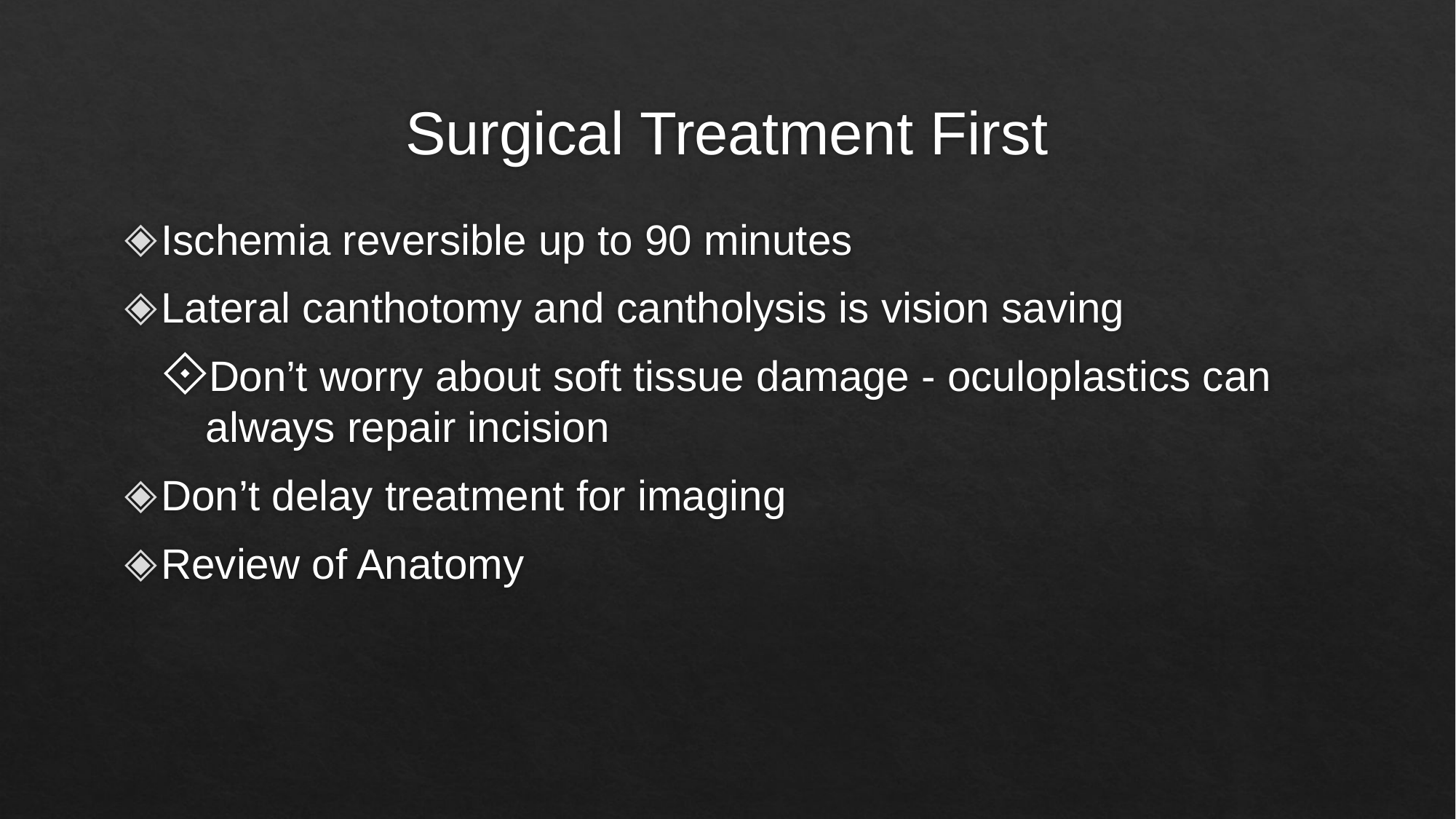

# Surgical Treatment First
Ischemia reversible up to 90 minutes
Lateral canthotomy and cantholysis is vision saving
Don’t worry about soft tissue damage - oculoplastics can always repair incision
Don’t delay treatment for imaging
Review of Anatomy

## Slide 13
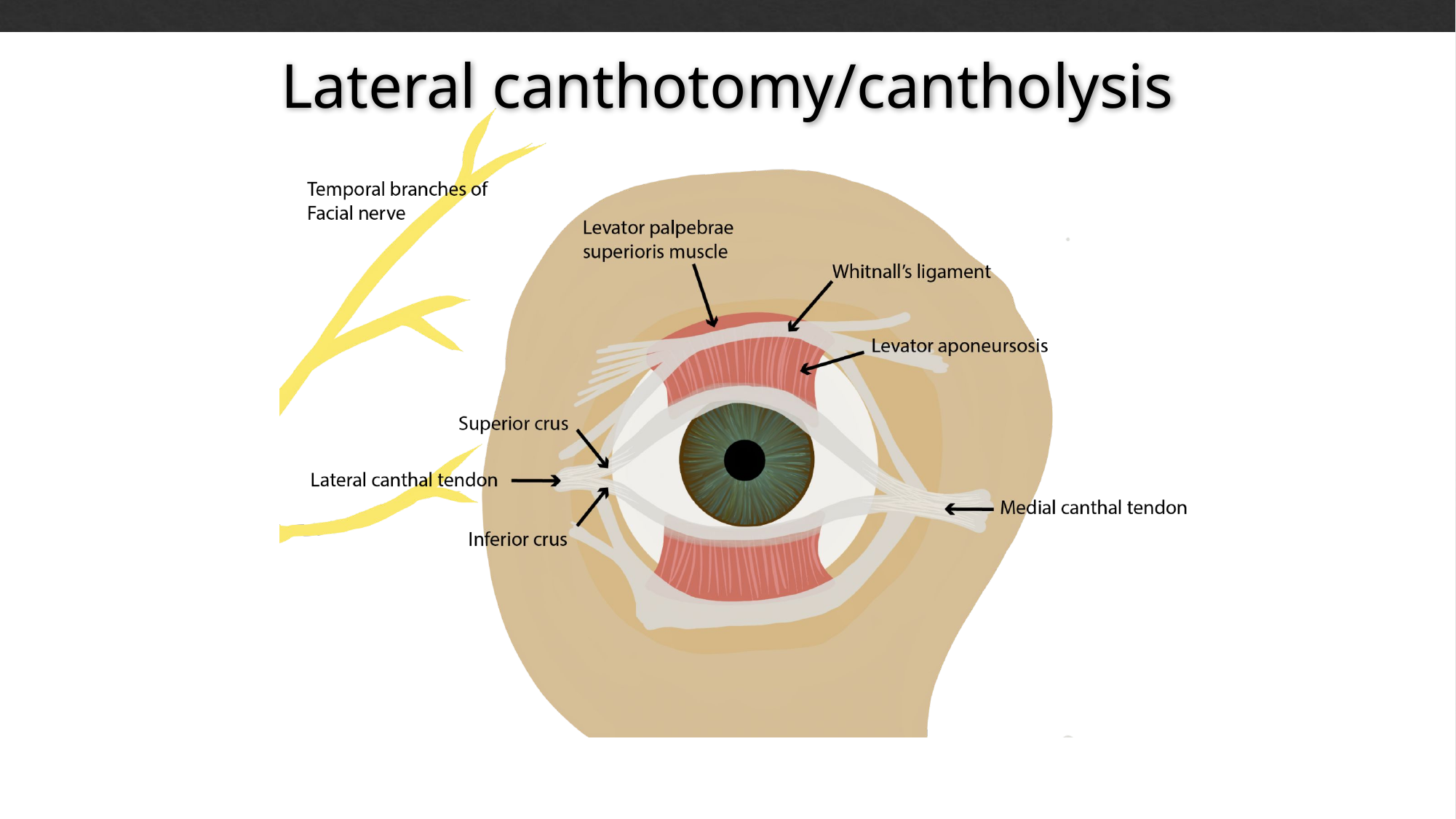

# Lateral canthotomy/cantholysis

## Slide 14
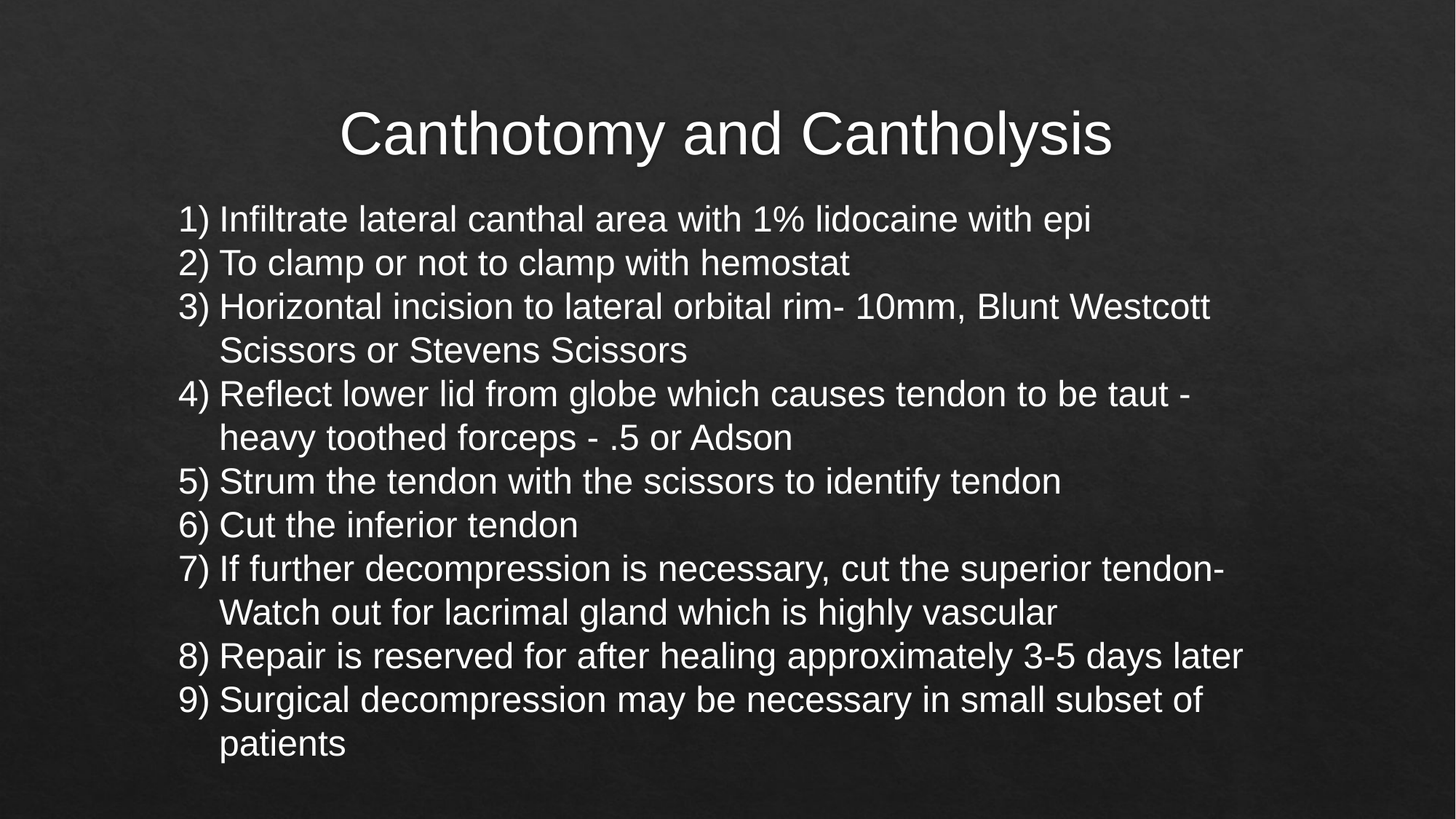

# Canthotomy and Cantholysis
Infiltrate lateral canthal area with 1% lidocaine with epi
To clamp or not to clamp with hemostat
Horizontal incision to lateral orbital rim- 10mm, Blunt Westcott Scissors or Stevens Scissors
Reflect lower lid from globe which causes tendon to be taut - heavy toothed forceps - .5 or Adson
Strum the tendon with the scissors to identify tendon
Cut the inferior tendon
If further decompression is necessary, cut the superior tendon- Watch out for lacrimal gland which is highly vascular
Repair is reserved for after healing approximately 3-5 days later
Surgical decompression may be necessary in small subset of patients

## Slide 15
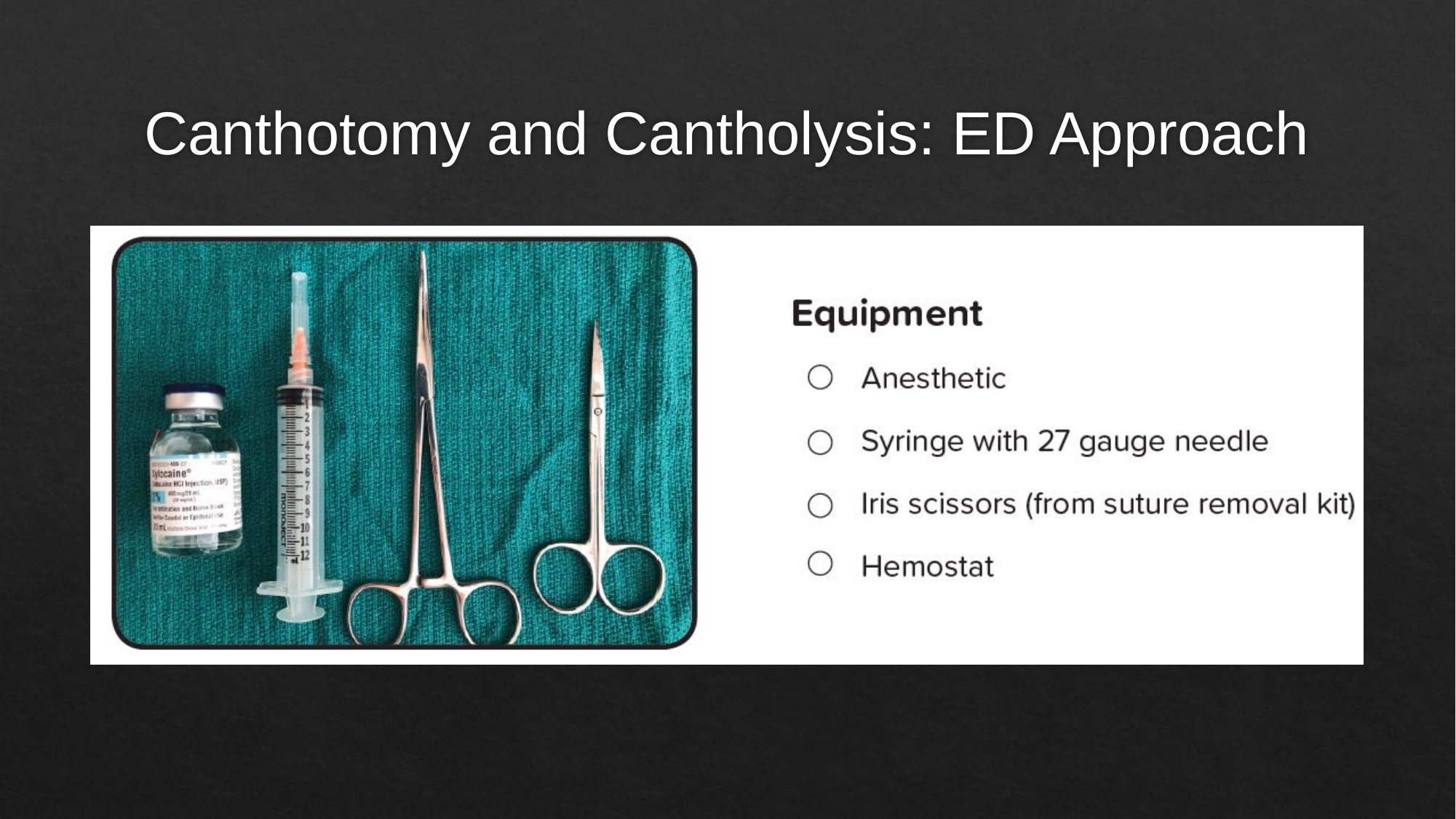

# Canthotomy and Cantholysis: ED Approach

## Slide 16
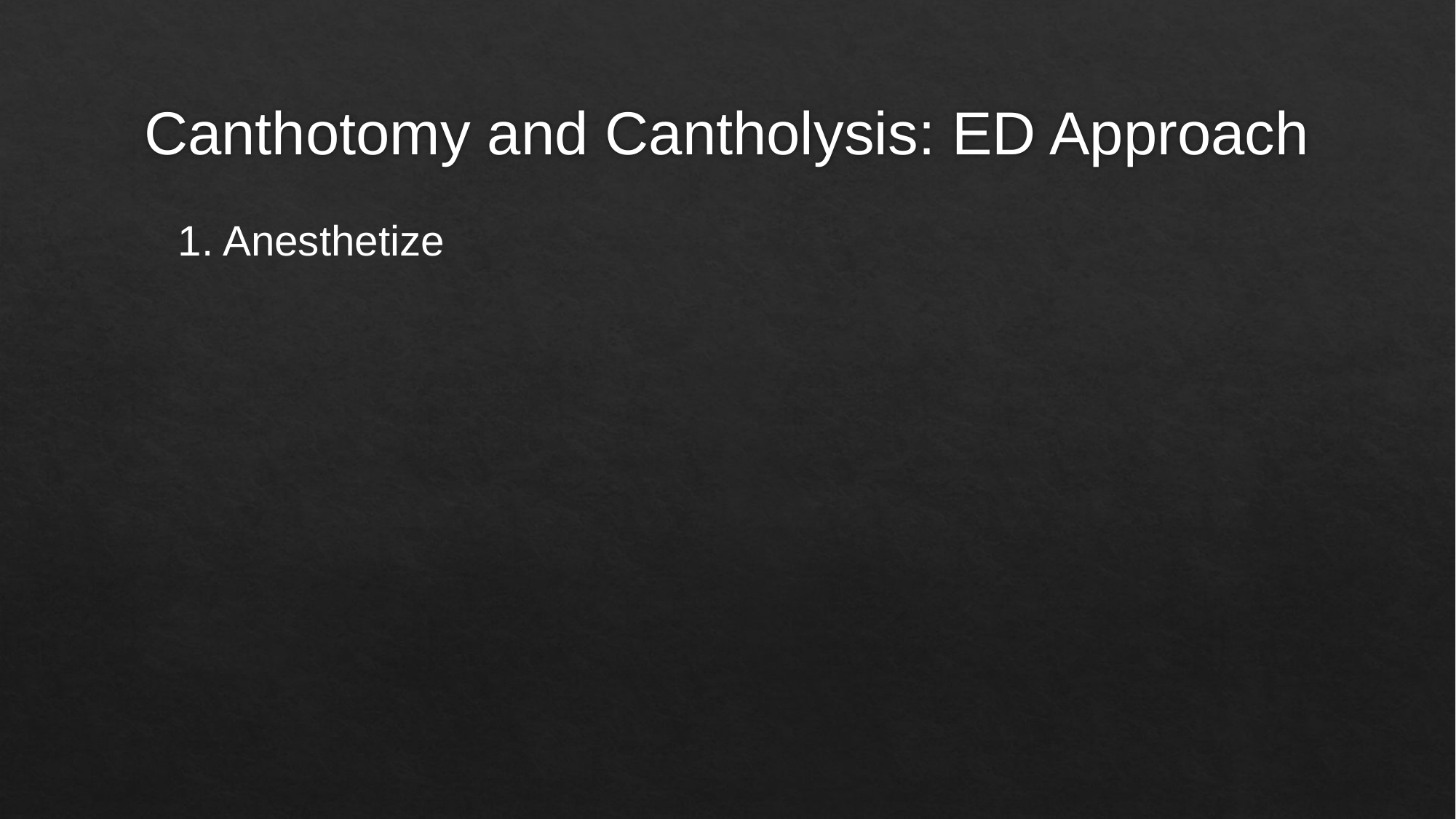

# Canthotomy and Cantholysis: ED Approach
1. Anesthetize

## Slide 17
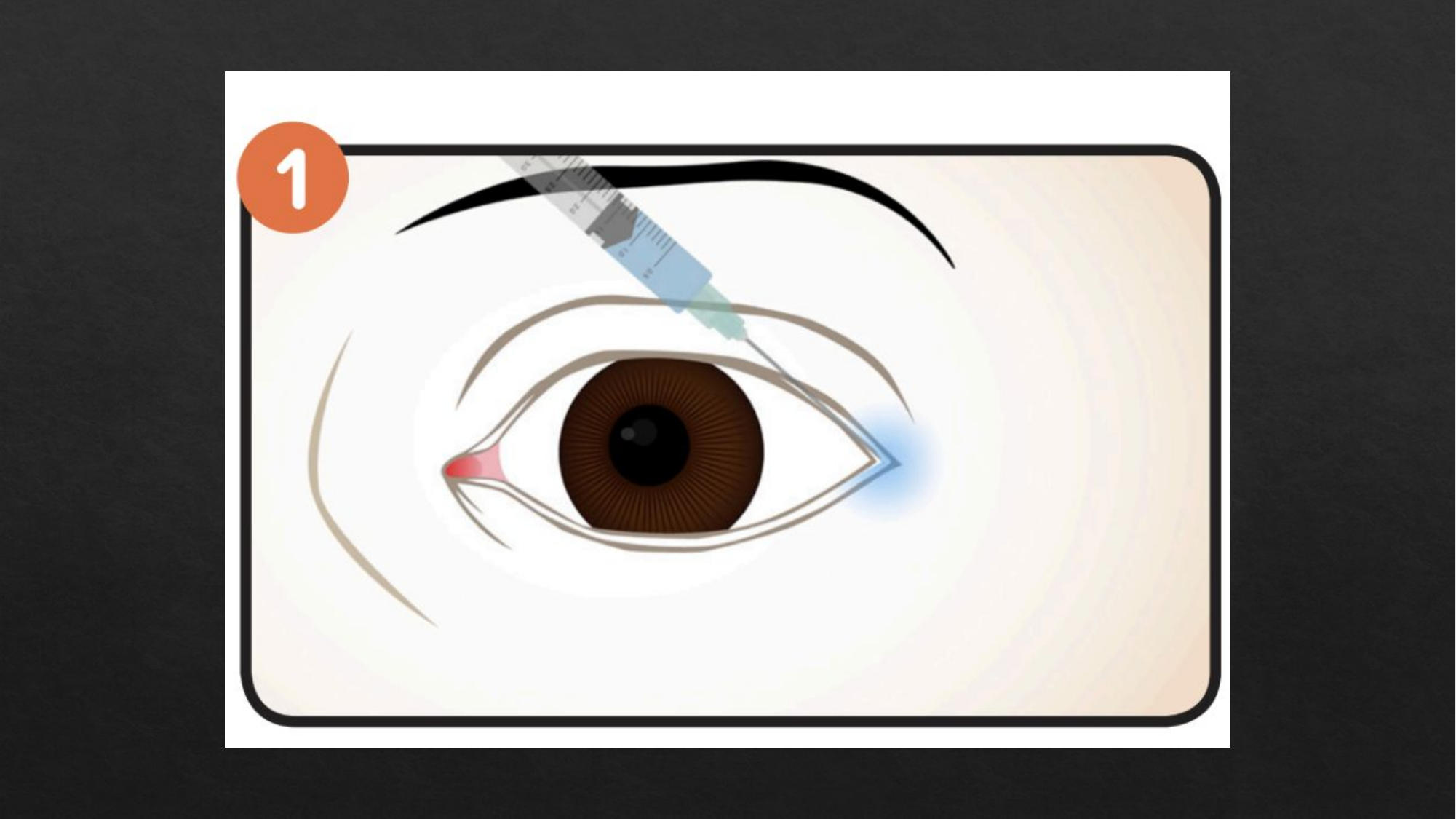

## Slide 18
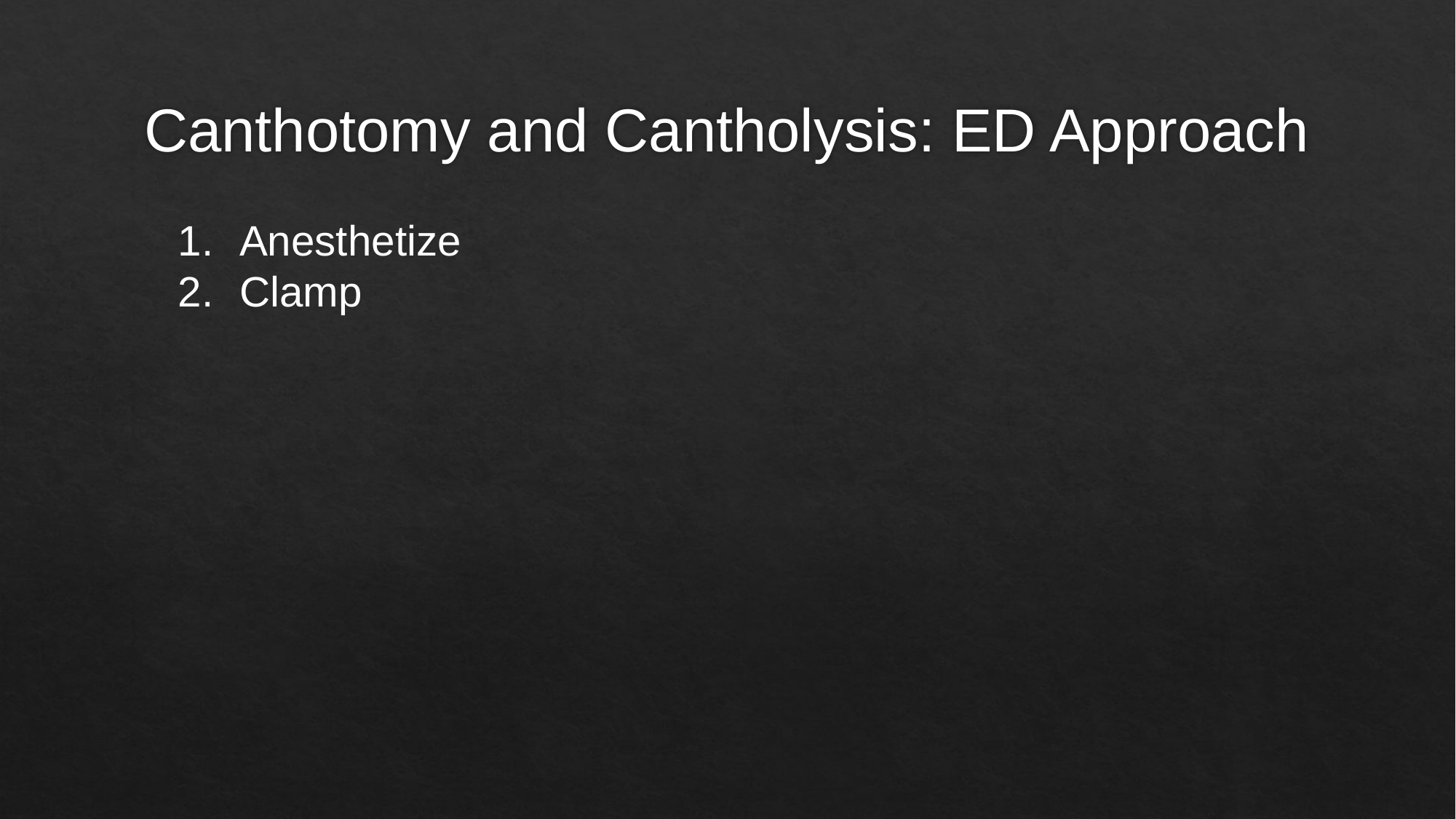

# Canthotomy and Cantholysis: ED Approach
Anesthetize
Clamp

## Slide 19
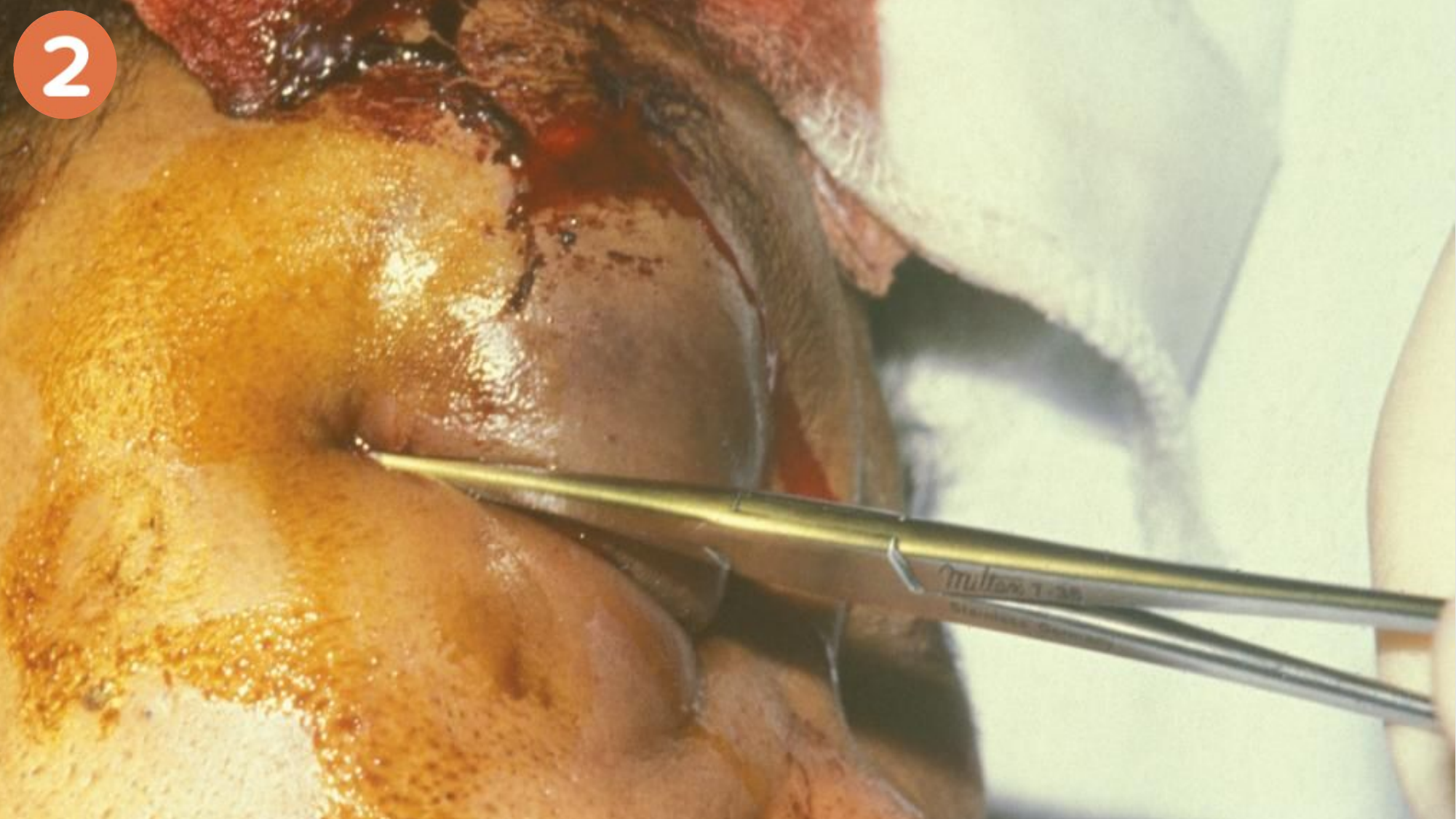

## Slide 20
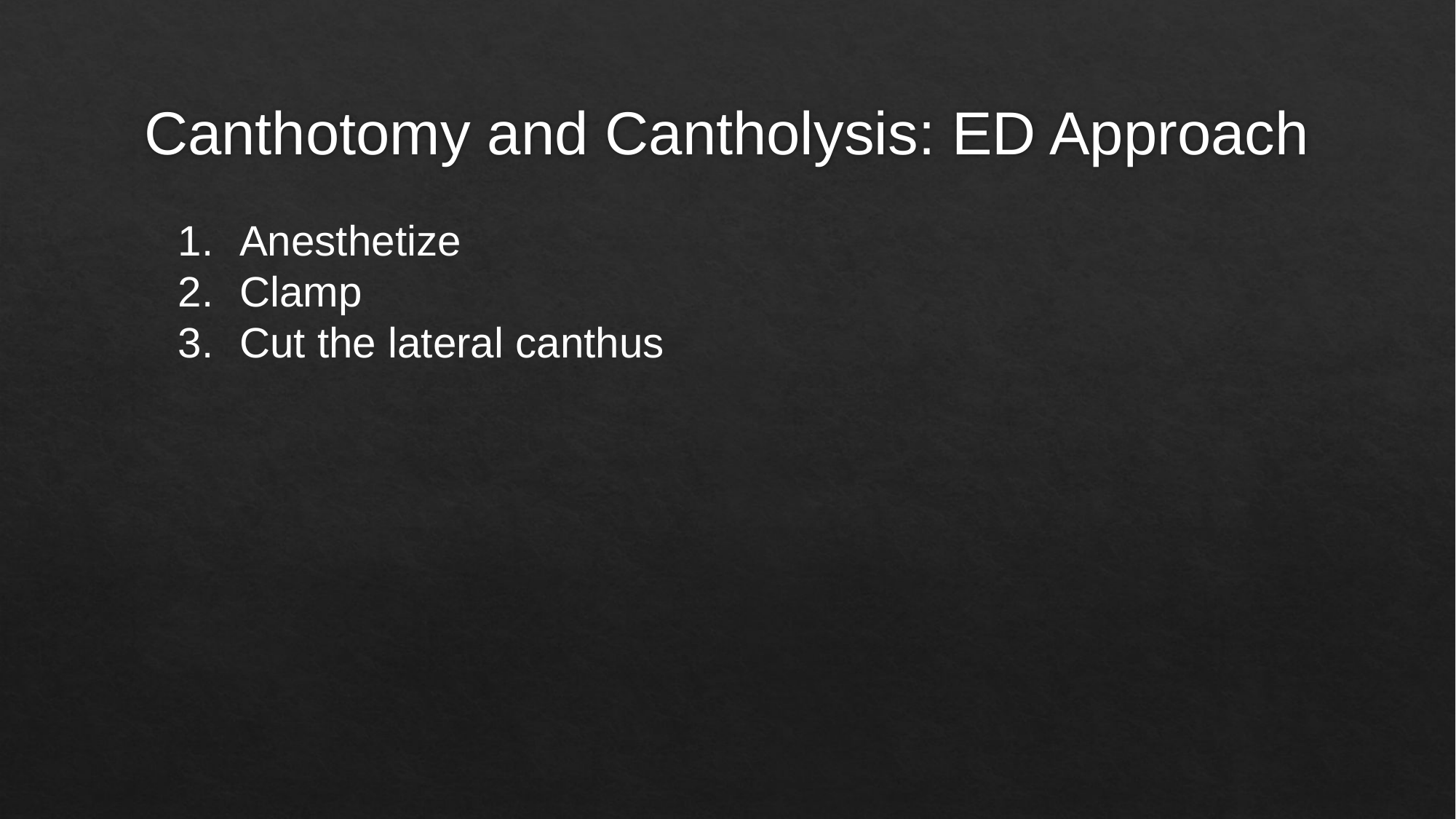

# Canthotomy and Cantholysis: ED Approach
Anesthetize
Clamp
Cut the lateral canthus

## Slide 21
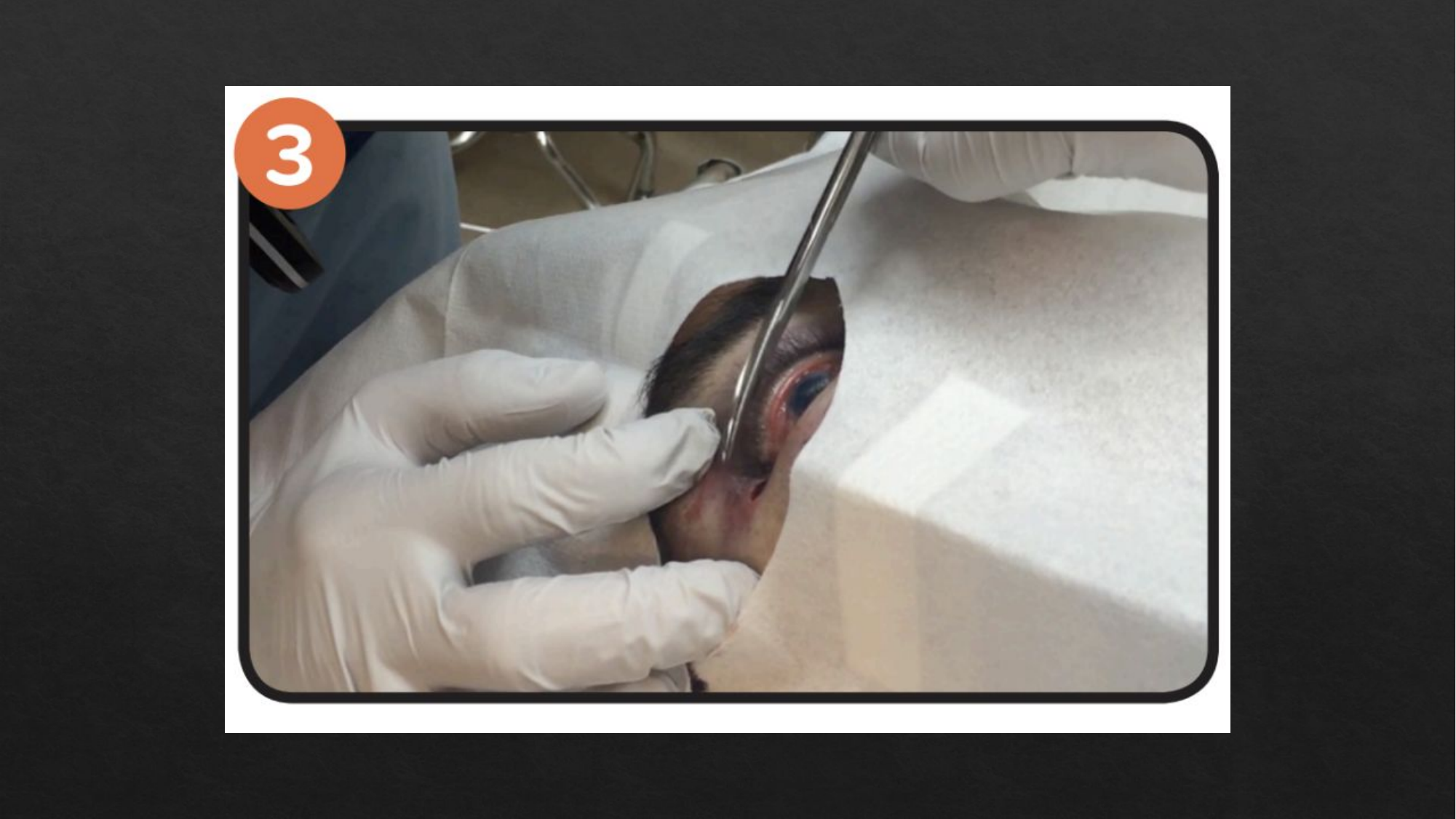

## Slide 22
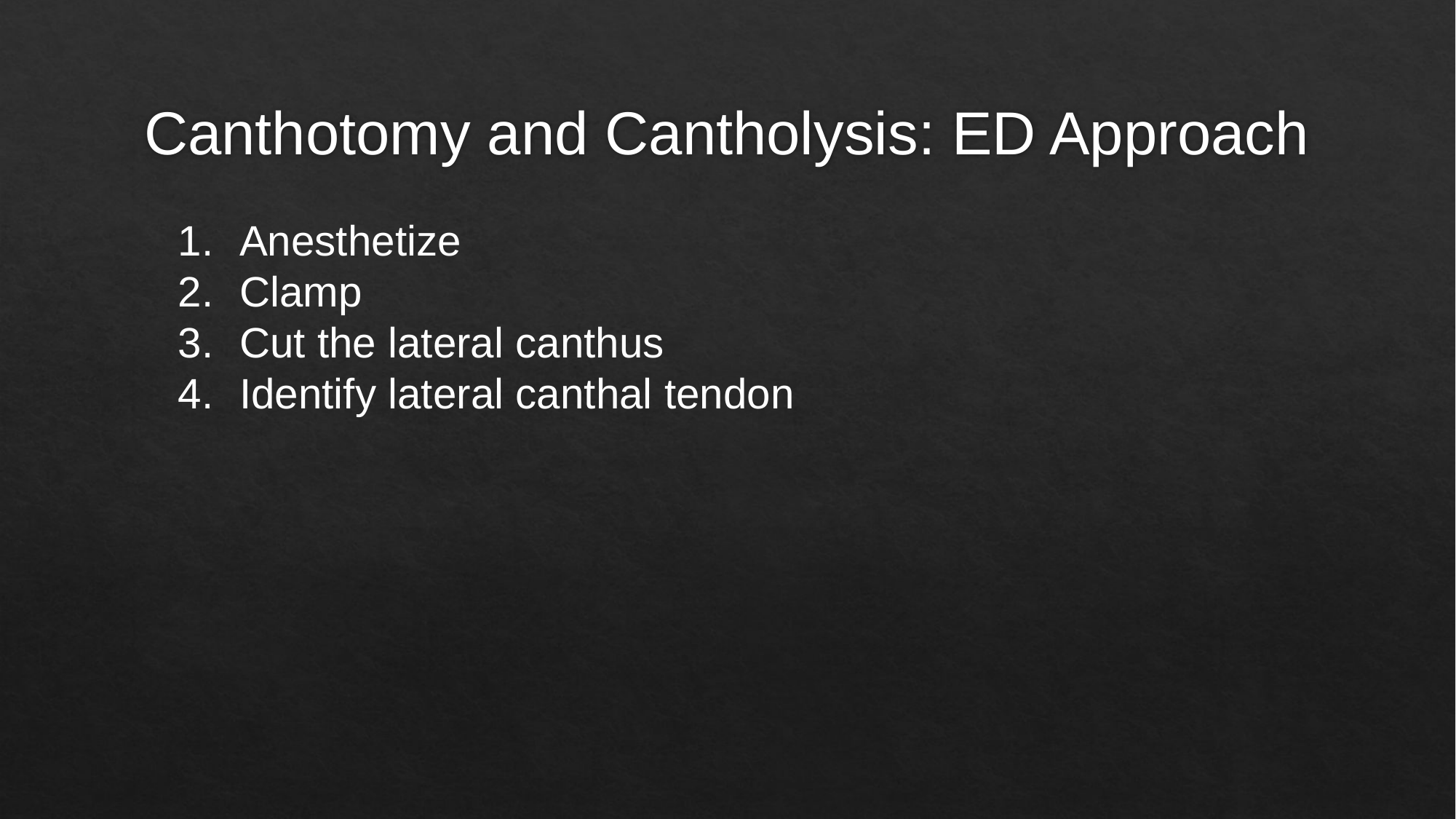

# Canthotomy and Cantholysis: ED Approach
Anesthetize
Clamp
Cut the lateral canthus
Identify lateral canthal tendon

## Slide 23
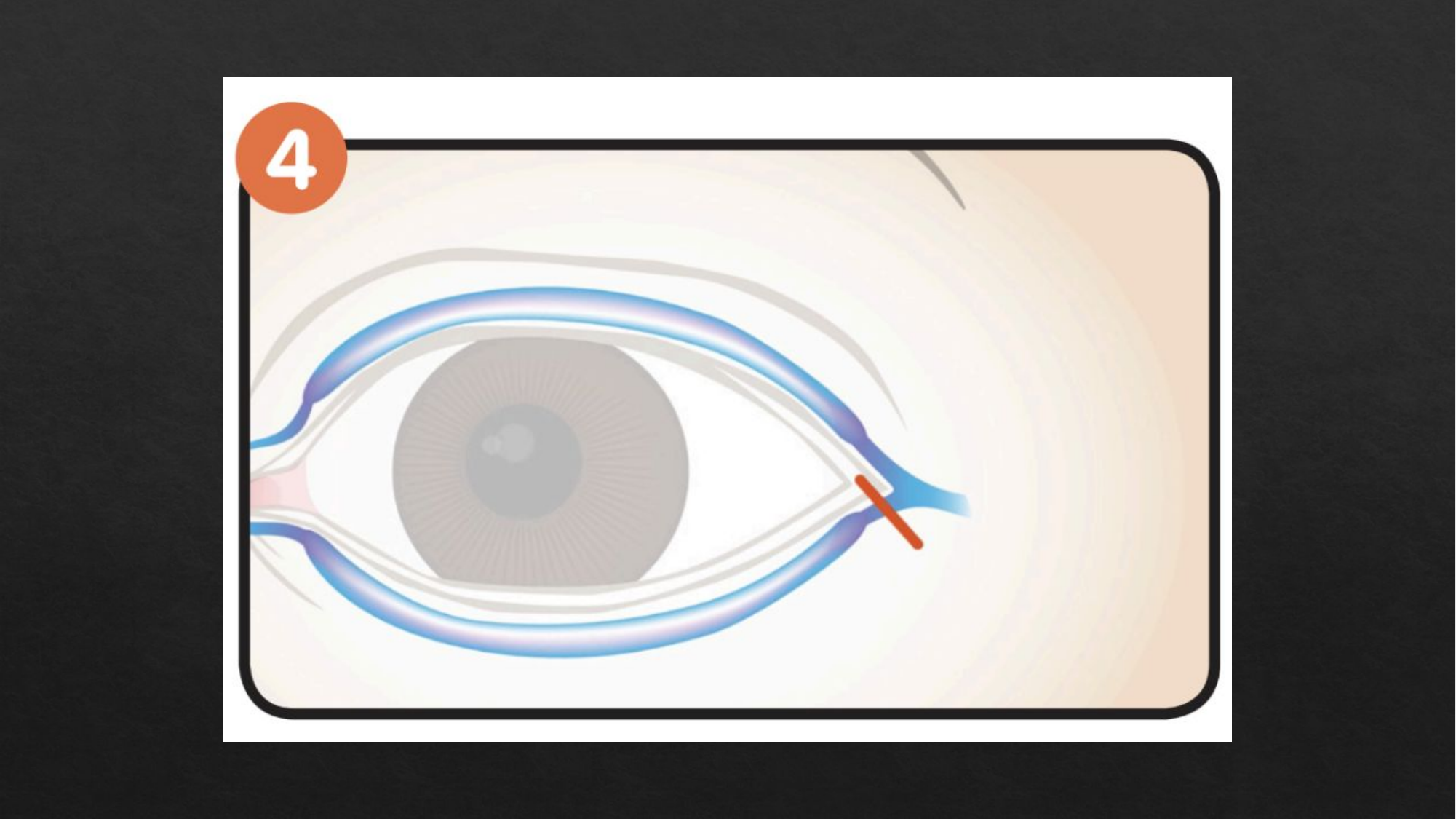

## Slide 24
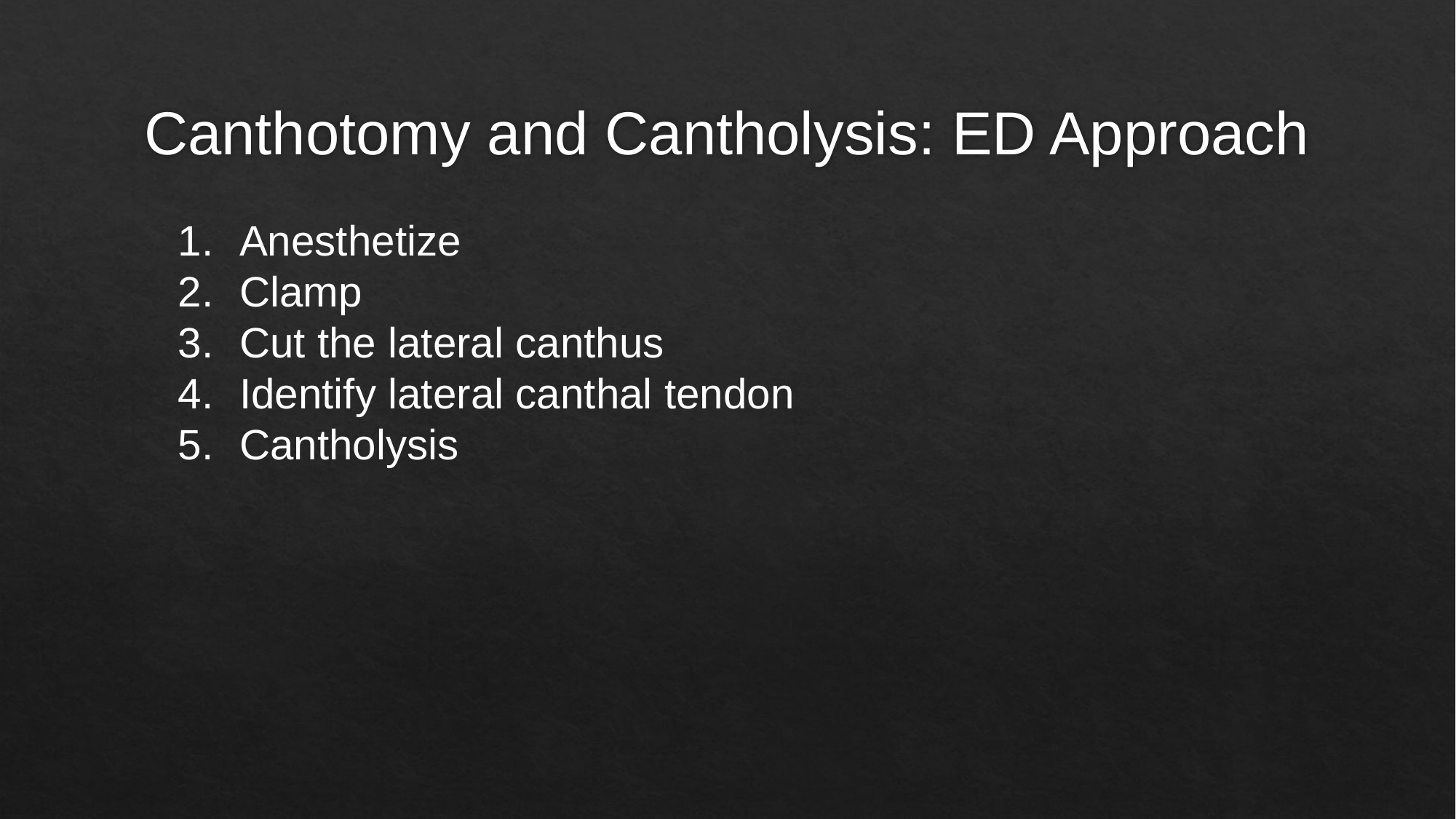

# Canthotomy and Cantholysis: ED Approach
Anesthetize
Clamp
Cut the lateral canthus
Identify lateral canthal tendon
Cantholysis

## Slide 25
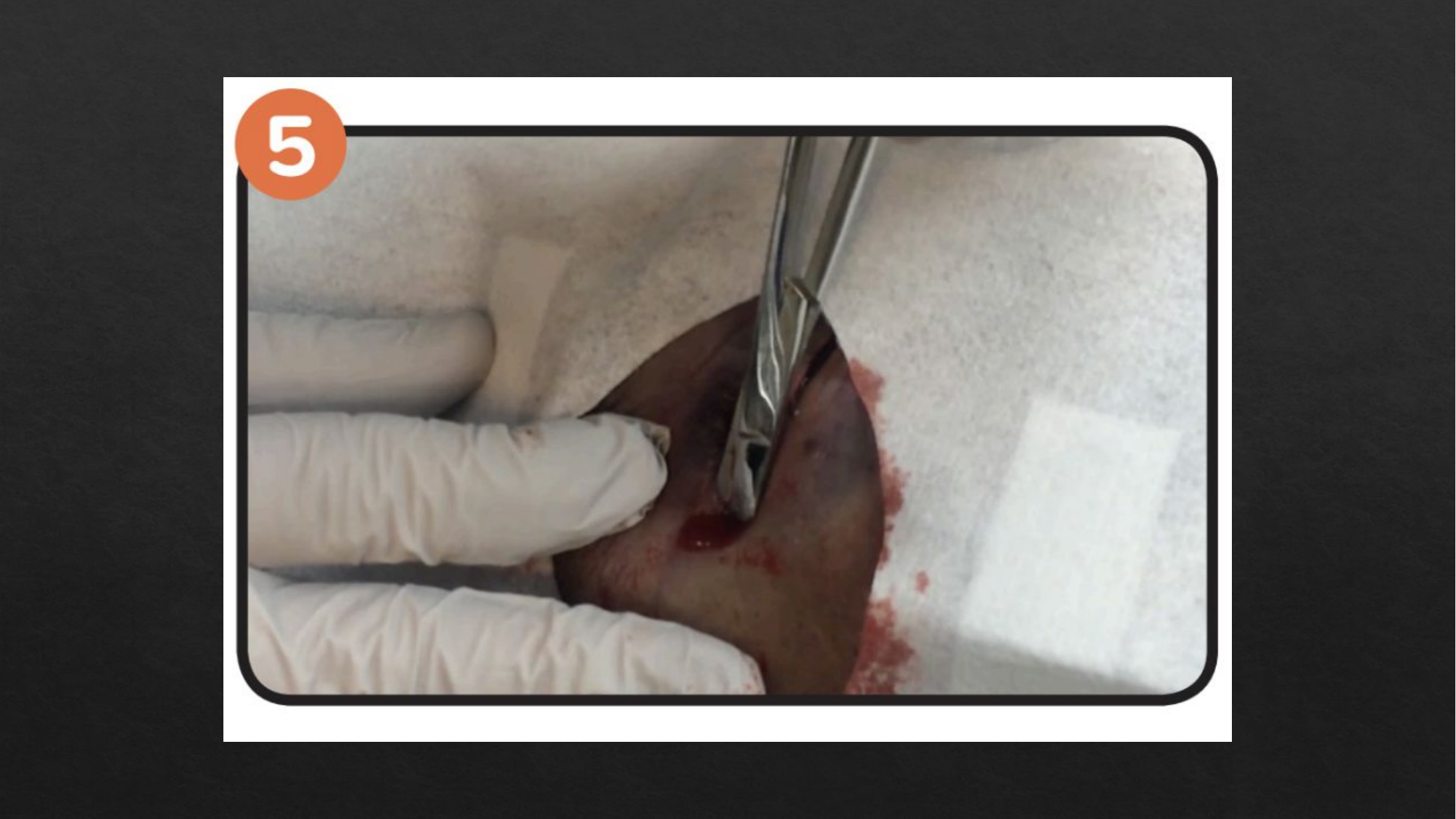

## Slide 26
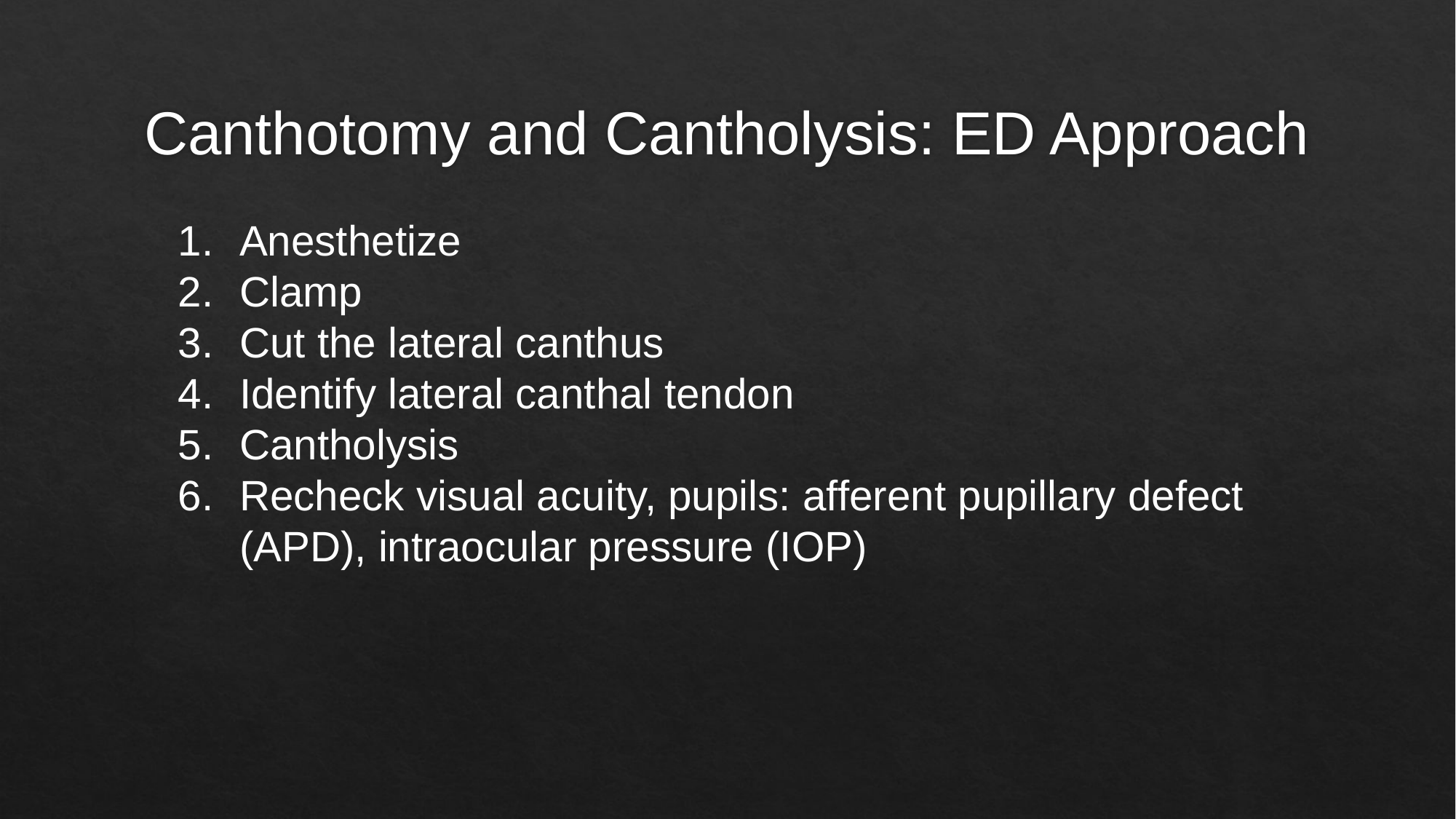

# Canthotomy and Cantholysis: ED Approach
Anesthetize
Clamp
Cut the lateral canthus
Identify lateral canthal tendon
Cantholysis
Recheck visual acuity, pupils: afferent pupillary defect (APD), intraocular pressure (IOP)

## Slide 27
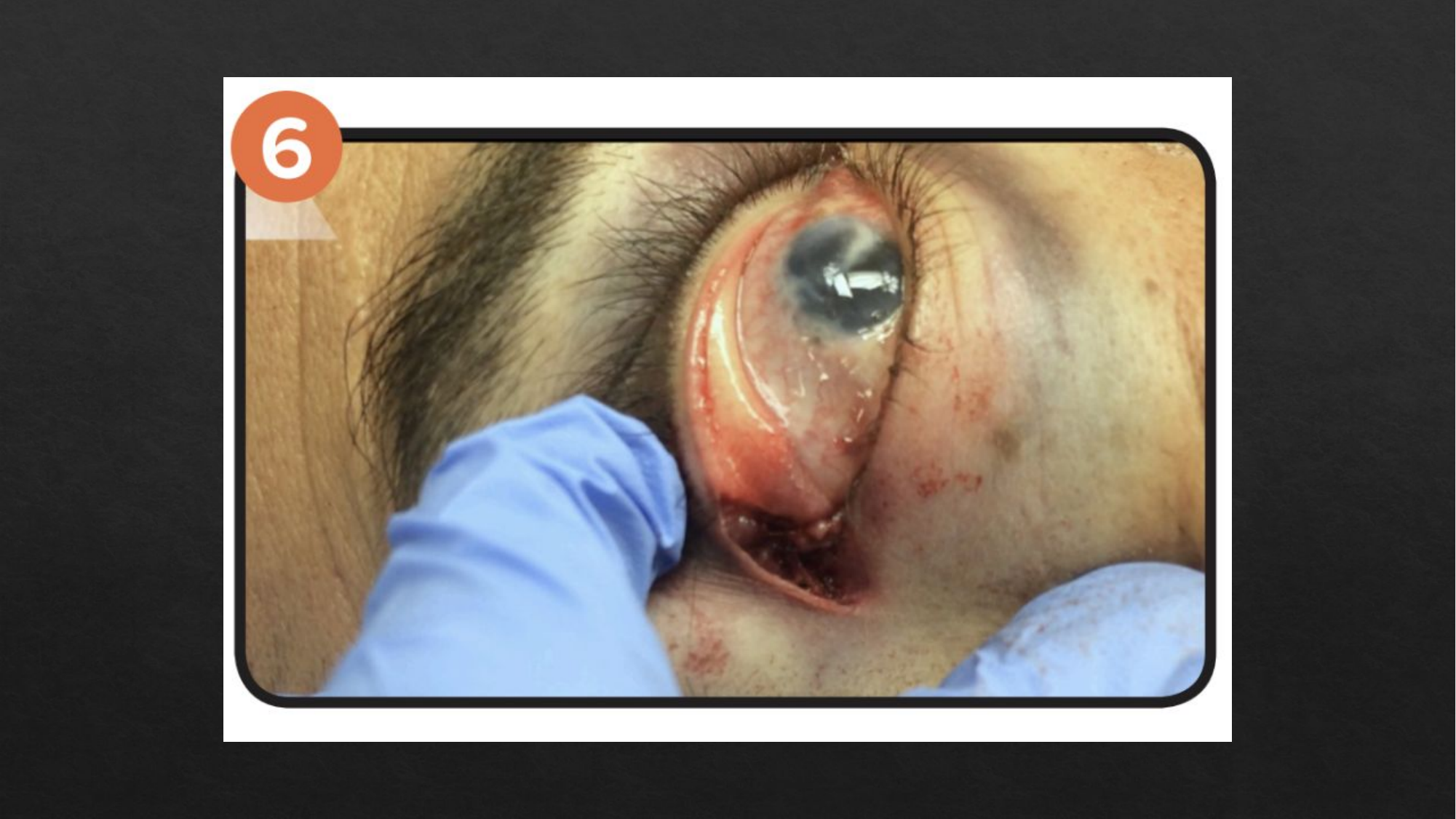

## Slide 28
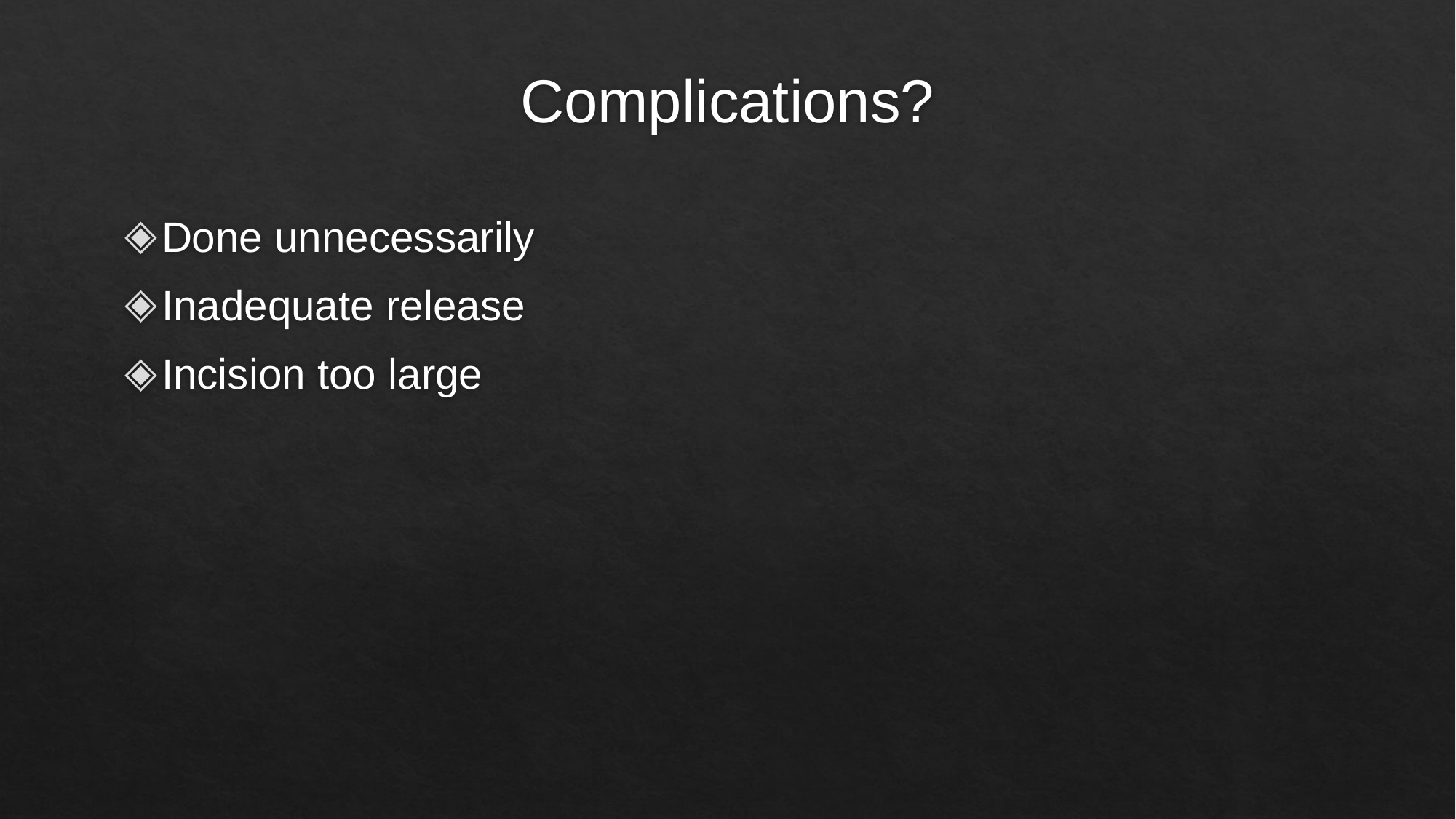

# Complications?
Done unnecessarily
Inadequate release
Incision too large

## Slide 29
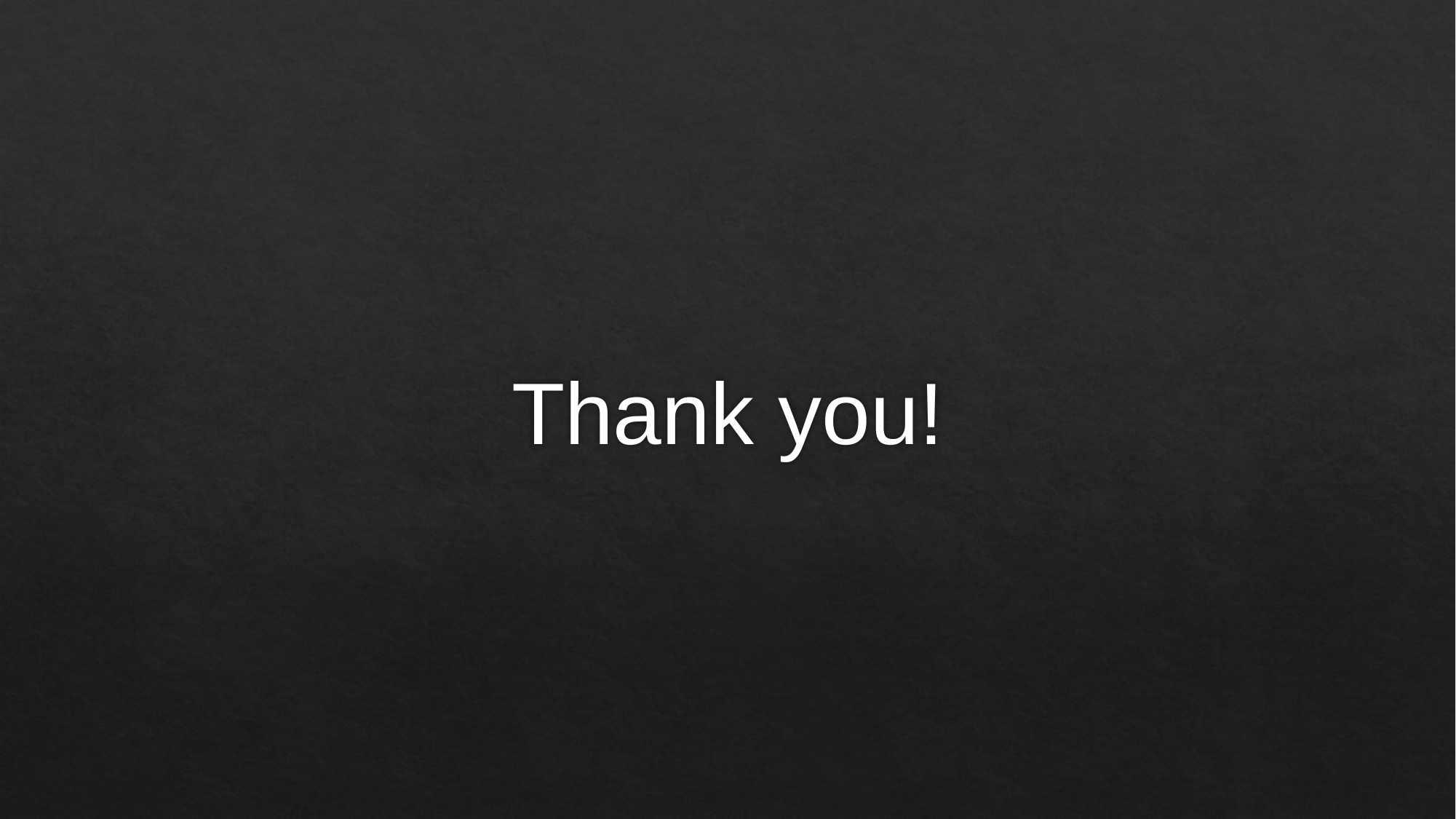

# Thank you!
