# Supplementary figures and images for: A Case of Painful Visual Loss - Managing Orbital Compartment Syndrome in the Emergency Department
Source: J Educ Teach Emerg Med. 2024 Oct 31;9(4):S1–S50. doi: 10.21980/J8N35D (PMC11537727; doi:10.21980/J8N35D)

## Slide 1
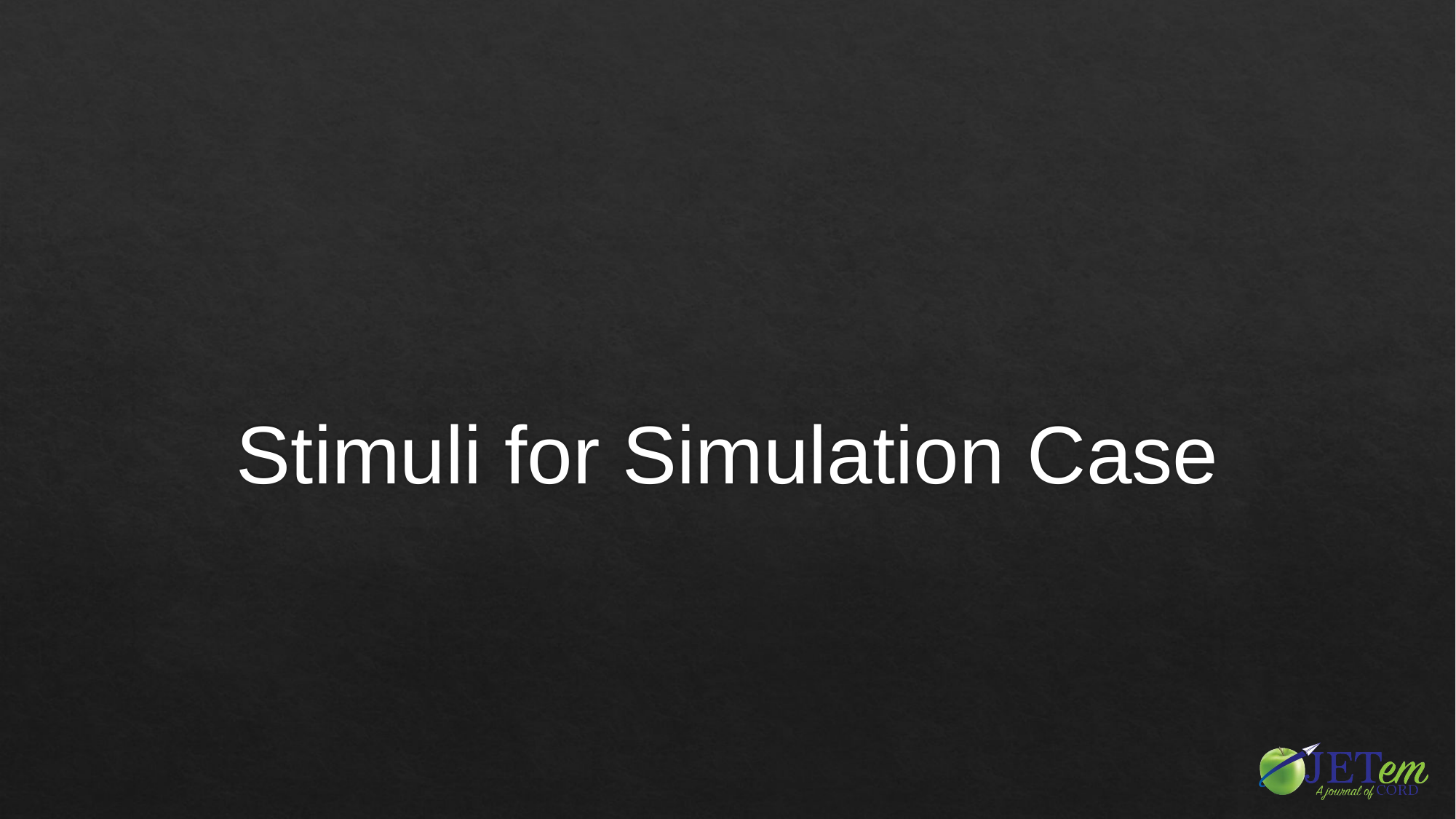

# Stimuli for Simulation Case

## Slide 2
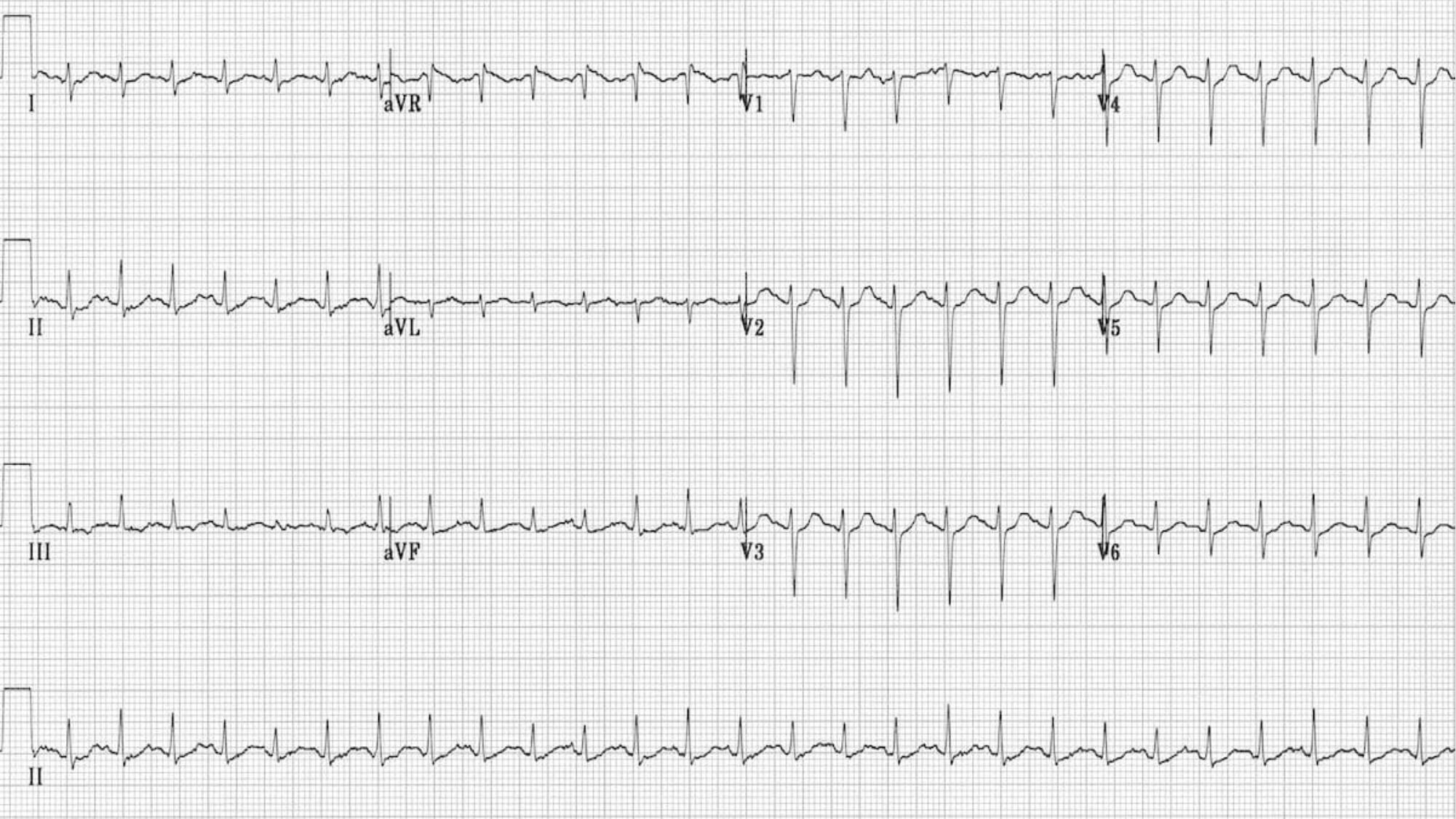

## Slide 3
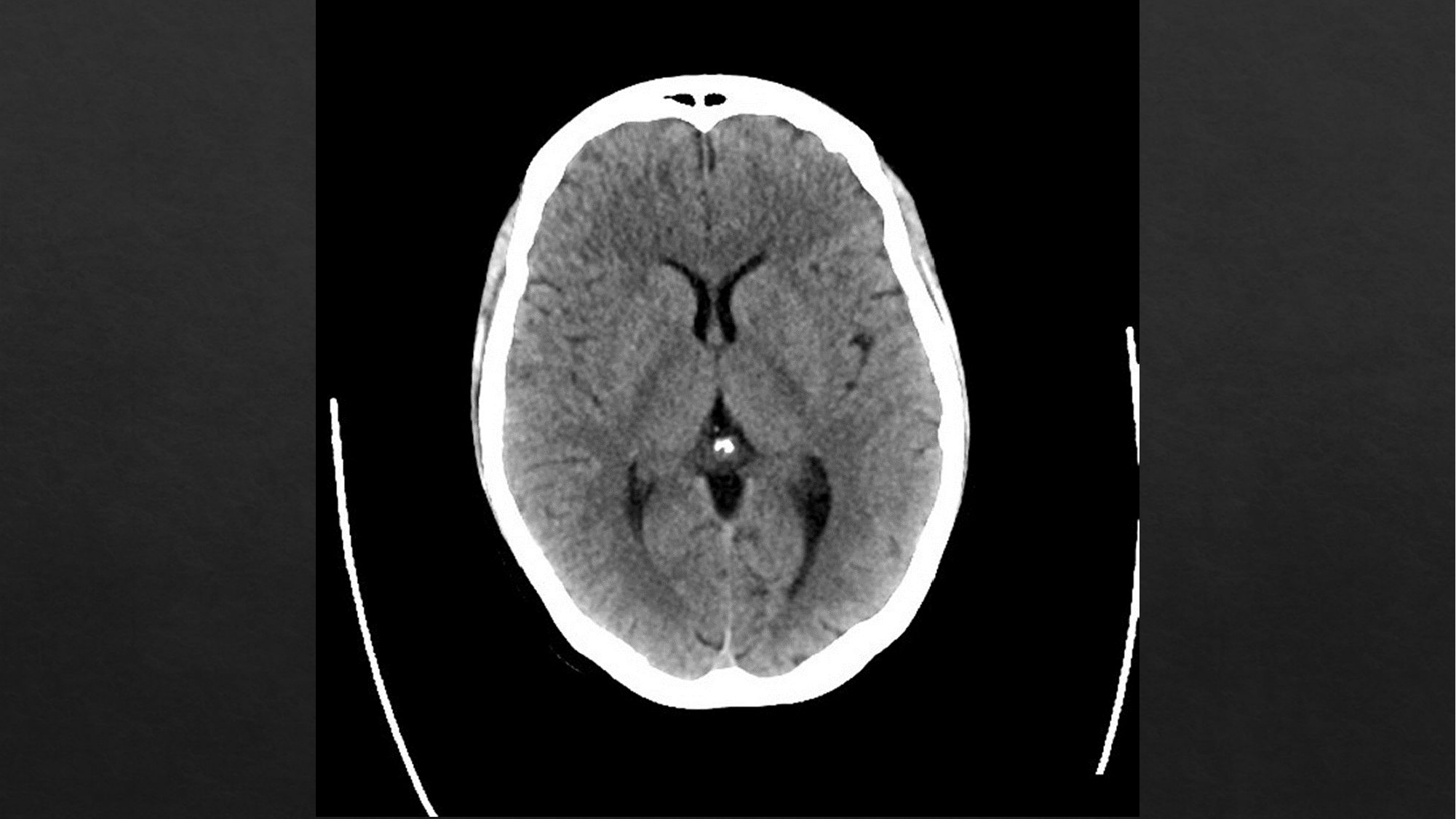

## Slide 4
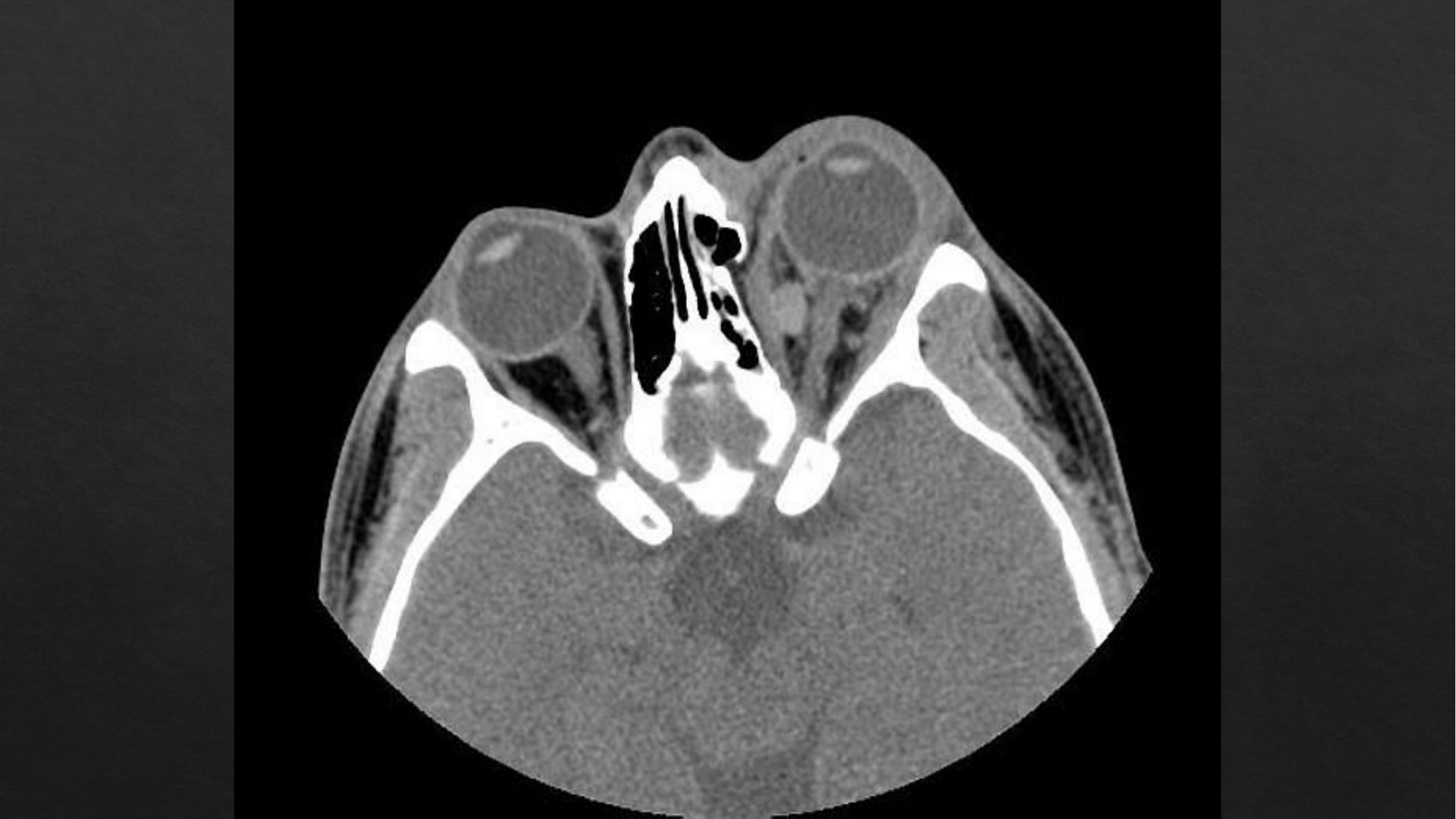

## Slide 5
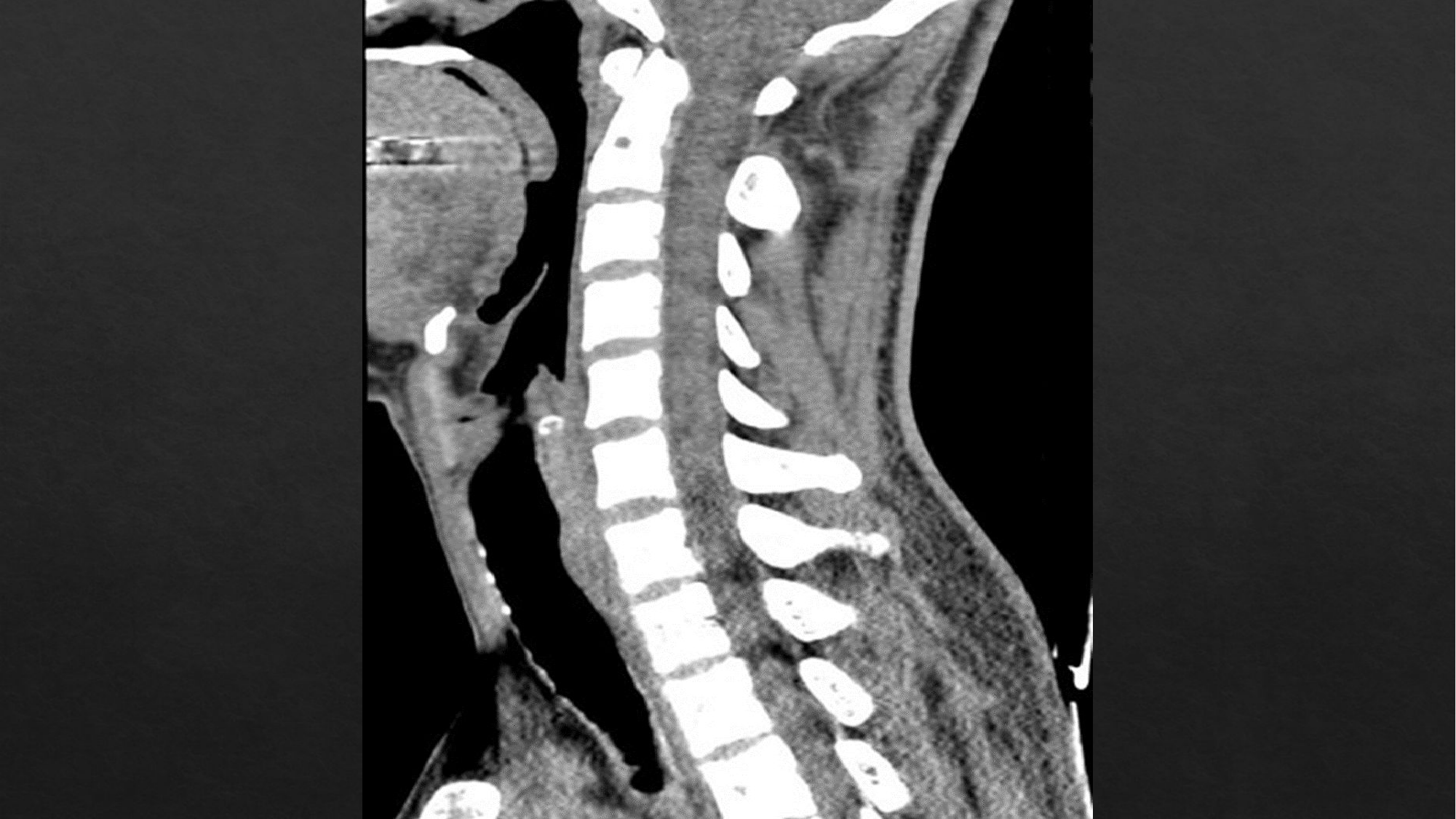

## Slide 6
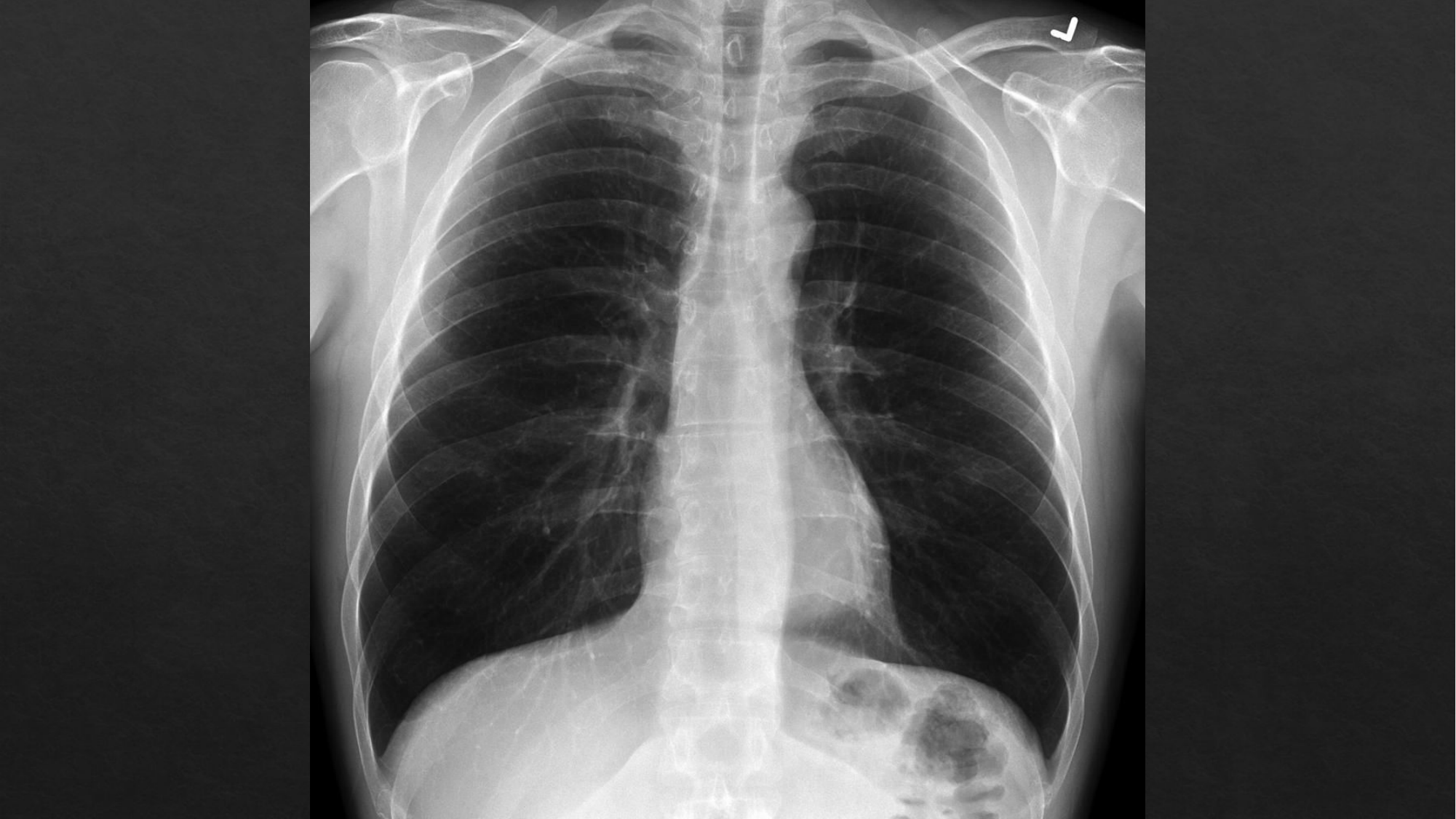

Supplement: Supplementary file 3 — Please see associated PowerPoint file [file 9-4-S1-Appendix_F.pptx]
